# An analysis of deficiencies in the data of interventional drug trials registered with Clinical Trials Registry - India

**DOI:** 10.1186/s13063-019-3592-0

**Published:** 2019-08-28

**Authors:** Mounika Pillamarapu, Abhilash Mohan, Gayatri Saberwal

**Affiliations:** Institute of Bioinformatics and Applied Biotechnology, Biotech Park, Electronics City Phase 1, Bengaluru, Karnataka 560100 India

**Keywords:** Clinical trials, CTRI, Data integrity, Missing data, Error rates, India, ClinicalTrials.gov

## Abstract

**Background:**

Clinical Trials Registry - India (CTRI) was established in July 2007 and today hosts thousands of trials, a significant fraction of them registered in the last couple of years. We wished to undertake an up-to-date analysis of specific fields of the registered trials. In doing so we discovered problems with the quality of the data, which we describe in this paper.

**Methods:**

We downloaded CTRI records and reformatted the data into an SQLite database, which we then queried. We also accessed ClinicalTrials.gov records as needed.

**Results:**

We discovered various categories of problems with the data in the CTRI database, including (1) a lack of clarity in the classification of *Types of Study*, (2) internal inconsistencies, (3) incomplete or non-standard information, (4) missing data, (5) variations in names or classification, and (6) incomplete or incorrect details of ethics committees. For most of these problems, error rates have been calculated, over time. Most were found to be in single digits, although others were significantly higher. We suggest how data quality in future editions of CTRI could be improved, including (1) a more elaborate and structured way of classifying the *Type of Study*, (2) the use of logic rules to prevent internal inconsistencies, (3) less use of free text fields and greater use of drop-down menus, (4) more fields to be made compulsory, (5) the pre-registration of individuals’ and organizations’ names and their subsequent selection from drop-down menus while registering a trial, and (6) more information about each ethics committee, including (a) its address and (b) linking the name of the trial site to the relevant ethics committee. As we discuss problems with the data of specific fields, we also examine — where possible — the quality of the data in the corresponding fields in ClinicalTrials.gov, the largest clinical trial registry in the world.

**Conclusions:**

It is a scientific and ethical obligation to correctly record all information pertaining to each trial run in India. CTRI is a valuable database that has proved its worth in terms of improving the record of trials in the country. The suggestions made herein would improve it further.

**Electronic supplementary material:**

The online version of this article (10.1186/s13063-019-3592-0) contains supplementary material, which is available to authorized users.

## Background

Clinical trials are interventional or observational experiments on humans. As such, they must be of the highest quality, and to ensure this there must be records both of the plan for the trial and what actually took place. These records should be accessible to the public and ideally should be auditable. One of the first calls for a clinical trial registry was made in 1986 [1], and since the year 2000 several registries have been set up around the world. The major ones are ClinicalTrials.gov in the USA and EudraCT in Europe. Although not a registry itself, the International Clinical Trials Registry Platform of the World Health Organization (WHO) is a platform which is linked to 18 registries of individual countries or of regions such as Africa or the European Union. Such registries host details of planned, ongoing, completed, suspended, and terminated trials, and this data is freely accessible to the public.

The data hosted by these registries is of interest to several categories of people, such as (1) patients, who wish to access experimental treatments; (2) funders, who wish to identify gaps in the landscape of medical innovation; (3) researchers, who wish to know what categories of medical innovation have reached the stage of trials; (4) trialists, who wish to avoid duplicating ongoing trials; and (5) policy makers, who may wish to know whether or not the trials being run in the country are relevant to local health needs. All of these stakeholders need access to accurate information. Inaccurate or incomplete information can lead to the suboptimal use of trial-related information, and research waste.

India has been considered an attractive location to conduct trials due to (1) the large number of patients, (2) the fact that many of these patients have little access to healthcare and therefore have not received treatment for their condition, (3) the considerable ethnic diversity in the country, (4) English being widely spoken in hospitals, and (5) the often poor regulatory oversight of trials [2]. It is reported that there are about 2.5 million trial volunteers in the country [3].

The government set up Clinical Trials Registry - India (CTRI) in July 2007 so that trials taking place wholly or partially in the country could be registered. Registration was initially voluntary, and whereas it was preferred that trials be registered prospectively, retrospective registration was permitted. In June 2009 registration became mandatory [4], and from 1 April 2018 prospective registration became mandatory [5]. The number of registered trials has increased rapidly in recent years, with 29 in 2008 [2], 155 in 2009 [2], 6474 in 2015 [6], 8969 on 30 June 2017 [7], 12,673 on 4 April 2018 [7], and 19,830 on 25 June 2019 [7].

There have been a few published analyses of the data in the CTRI database [2, 4, 6, 8–16]. However, given that the number of registered trials increased significantly in 2018, we wished to undertake an up-to-date analysis of various fields of the registered trials. In doing so we discovered various problems with the quality of the data. In this paper we describe some of these problems and quantify most of them over time. We also examine — where possible — the quality of the data in the corresponding fields in ClinicalTrials.gov, the largest clinical trial registry in the world, with more than 300,000 records. Further, we suggest how the quality of data in future editions of CTRI could be improved. Finally, we note two concerns related to accessing data in the CTRI database.

It is a scientific and ethical obligation to correctly record all information pertaining to each trial that has been initiated in India. CTRI is a valuable database that has proved its worth in terms of improving the record of trials in the country. The suggestions made herein would improve it further.

## Methods

We accessed the CTRI records at http://ctri.nic.in/Clinicaltrials/advancesearchmain.php. We began this work on 4 April 2018, and at that time CTRI hosted 12,673 trials. Each record of a given trial is in the form of an HTML file in a standard format. The list of fields and their descriptions are available in a document entitled CTRI_Dataset_and_Description.pdf. This document is available at http://ctri.nic.in/Clinicaltrials/login.php where one clicks on “Trial Registration Data Set Download:[Pdf]”. We provide a sample CTRI record as Additional file 1.

We used an in-house script written in Python (Additional file 2, available at https://osf.io/uh7j4), which is used as a web-scrapping bot as well as a parser to reformat the data available in the HTML records into an SQLite database to make it suitable for analysis. The SQLite database is available as Additional file 3 at https://osf.io/uh7j4/, and the schema of the database is available in Additional file 4. The SQLite queries and Excel commands used to generate the data are provided in Additional file 5.

In most of the following sections, we quantify the error rates. In such cases, there is a subsection entitled “Quantification of problem over time”. In each, we examined the percentage of trials with errors over time, in four 3-year intervals. The time periods were 2007–2009, 2010–2012, 2013–2015, and 2016–2018 (with data for 2018 only up to 3 April). This was done for the Indian set of trials, and for the Multinational set if applicable.

AM wrote the scripts to download, clean up, and format the data into an SQLite database. MP wrote the SQL queries to search that database. AM checked the SQL queries and also the results of these queries. AM or GS manually cross-checked the output information and overall results.

In the following results and discussion, field names are italicized. Finally, we accessed ClinicalTrials.gov at https://clinicaltrials.gov/.

## Results

**Fig. 1:**
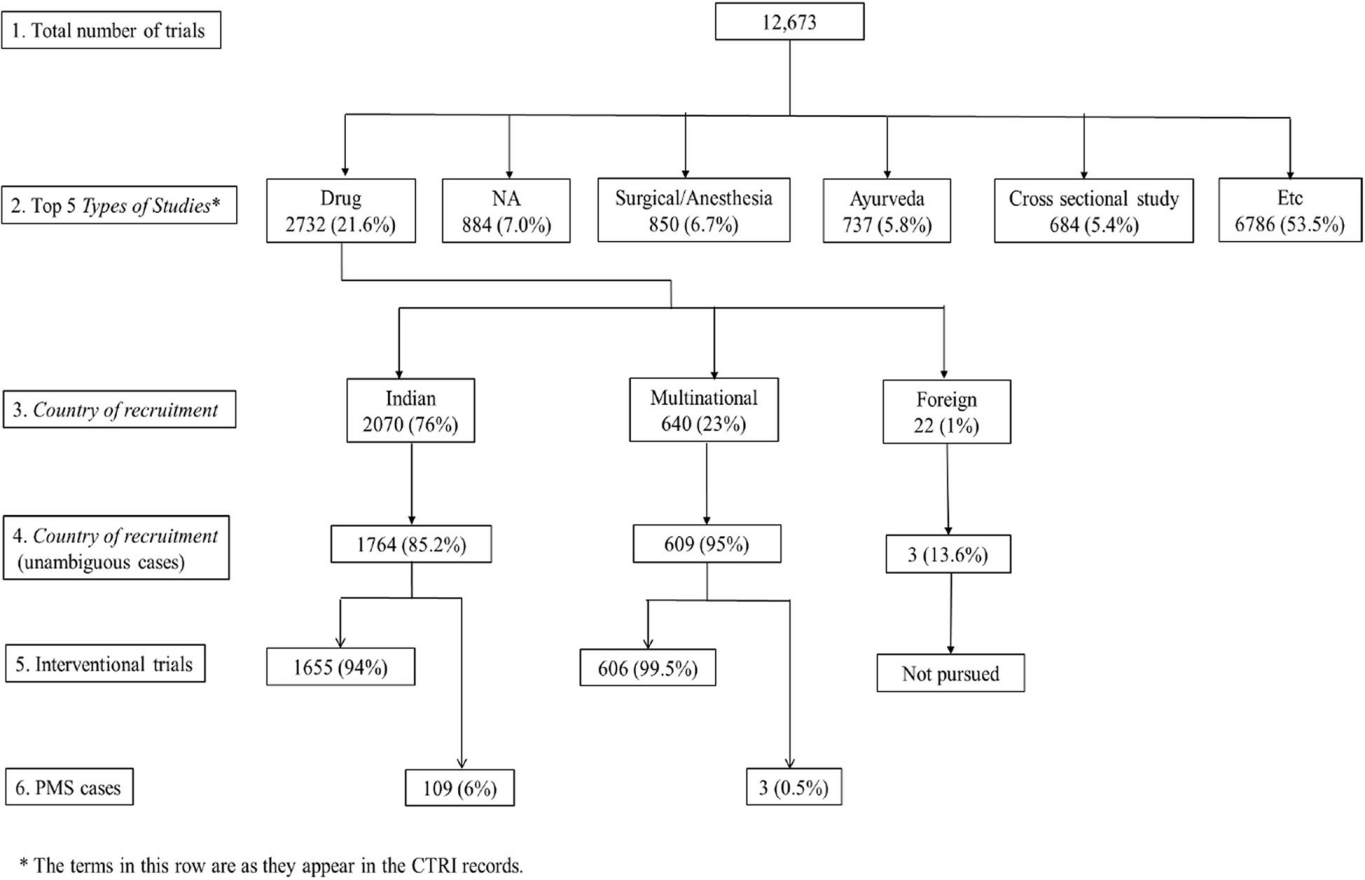
Data processing for the first few sections of results. (1) The 12,673 trials downloaded from CTRI; (2) the top 5 categories of *Type of Study* (first Results section in the paper); (3) the three categories of trials based on the *Countries of Recruitment* field, i.e., Indian, Multinational, and Foreign (second Results section in the paper); (4) the unambiguous cases of Indian, Multinational, and Foreign trials (second Results section); (5) the Interventional cases of Indian and Multinational trials (third Results section in the paper); and (6) the PMS cases of Indian and Multinational trials (fourth Results section in the paper)

**Fig. 2:**
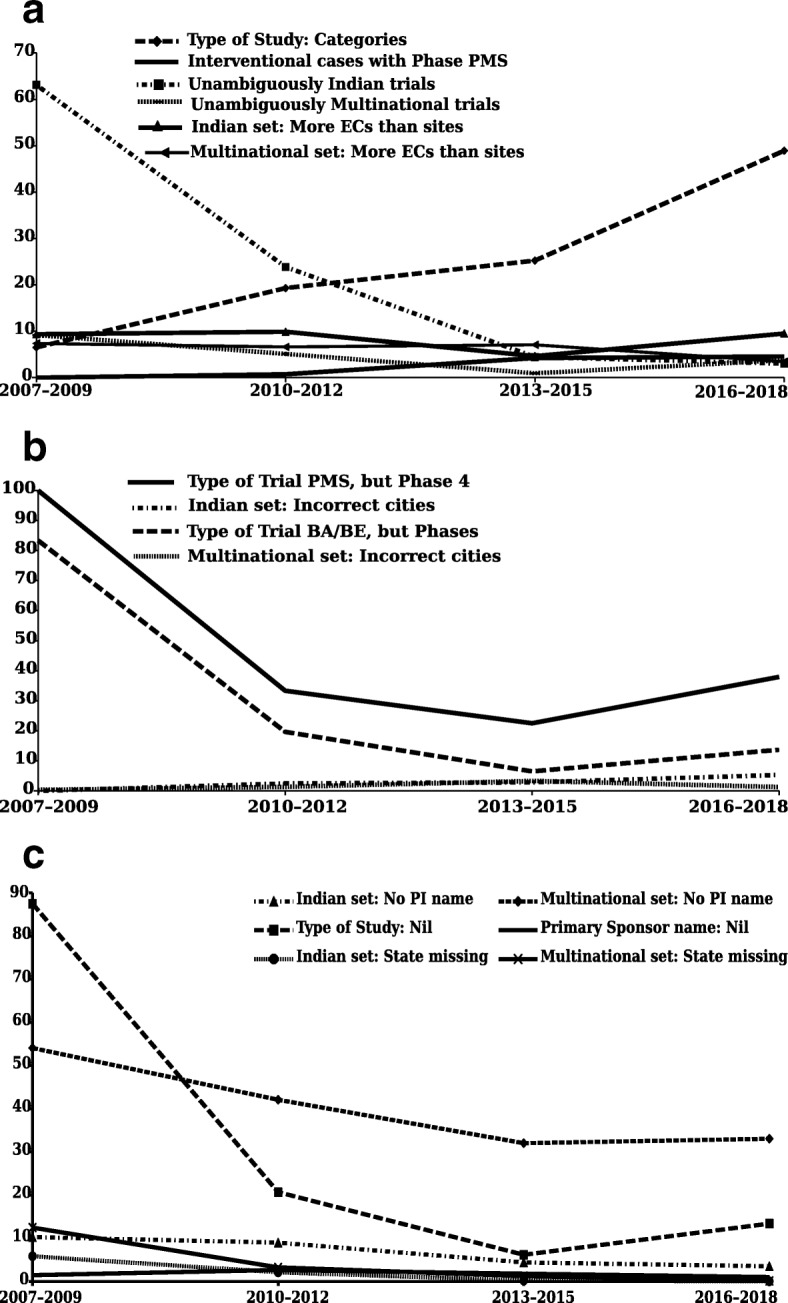
The percentage of trials with errors, in four 3-year time periods, for the several categories of errors. **a** Error rates (1) for *Type of Study*: Large number of categories (although this is not strictly an “error rate”, we have labeled it as such since all other problems reported here are error rates); (2) in determining the unambiguously Indian trials; (3) in determining the unambiguously Multinational trials; (4) for Indian trials: Interventional cases with Phase listed as PMS; (5) for Indian trials: More ethics committees than trial sites; and (6) for Multinational trials: More ethics committees than trial sites. **b** Error rates for the (1) redefined Indian trials: *Type of Trial* was PMS, but *Phase of Trial* was Phase 4; (2) redefined Indian trials: *Type of Trial* was BA/BE, but Phase of trial was 1, 1/2, 2, 2/3, 3, 3/4, or 4; (3) Indian trials: incorrect listing of cities; and (4) Multinational trials: incorrect listing of cities. **c** Error rates for the (1) Indian trials: PI names listed as nil; (2) Multinational trials: PI names listed as nil; (3) redefined Indian trials: *Type of Study* was NA, but Phase of trial was 1, 1/2, 2, 2/3, 3, 3/4, or 4; (4) Indian trials: Primary Sponsor name nil; (5) Indian trials: state of trial site missing; and (6) Multinational trials: state of trial site missing

We present our findings of problems with the CTRI database below. Data processing for the first few sections of results are presented in Fig. 1. For most of these problems, error rates have been calculated over time, and are presented in Fig. 2 and Additional file 6. Aside from the results, we identified two challenges in accessing the data. These are also described below.

### Type of Study

We first examined the *Type of Study* of the 12,673 trials. There were 1331 categories, which are listed, along with their frequencies in Additional file 5. The top five categories (Fig. 1) were (1) drugs (2732 or 22%), (2) Not Available (884, 7%), (3) Surgical/Anesthesia (850, 7%), (4) Ayurveda (737, 6%), which is a system of alternative medicine practiced in India, and (5) Cross Sectional Study (684, 5%).

Quantification of problem over time: In Fig. 2a and Additional files 5 and 6, we quantify the problem of too many categories of *Types of Study*. We examined the number of categories, with respect to the number of trials over time, in four 3-year intervals. The percentages were 6.5, 19.3, 25.2, and 48.9, respectively. As such, the number of categories increased more than sevenfold from time period one to four. Although this is not strictly an ‘error rate’, we have labeled it as such in Fig. 2a, since all other problems quantified in Fig. 2 are error rates.

In ClinicalTrials.gov, the equivalent field was *Intervention*. This had 11 categories: Behavioral, Biological, Combination Product, Device, Diagnostic Test, Dietary Supplement, Drug, Genetic, Other, Procedure, and Radiation. One category could be chosen multiple times, and more than one category could also be chosen. However, in downloaded data, multiple interventions were listed in a discrete and unambiguous manner. We give three examples of this, starting with the unique ID of the trial concerned: (1) NCT00736645 – Dietary Supplement: selenomethionine|Drug: finasteride|Other: placebo; (2) NCT01282515 – Drug: clobetasolpropionate|Drug: hexaminolevulinate; and (3) NCT00787969 – Biological: rituximab|Drug: cladribine|Drug: temsirolimus|Biological: Filgrastim|Biological: Pegfilgrastim.

In the following sections, we focused our attention on the largest category of trials, i.e., drugs, in all the sections except two, which are specified.

### Countries of Recruitment

We then investigated the *Countries of Recruitment* of the 2732 drug trials. There were 2070 (76%) trials conducted only in India (hereafter, Indian trials), 640 (23%) were conducted in India as well as in other countries (Multinational trials), and 22 (1%) were conducted only outside India (Foreign trials), as shown in Fig. 1.

We looked at the set of 22 Foreign trials more closely (Additional file 5). Although none of them listed India as a Country of Recruitment, in one case no country was listed. Further examination of this case showed that (1) *Recruitment Status of Trial (Global)* was “Not applicable” and *Recruitment Status of Trial (India)* was “Open to recruitment”; (2) *Date of First Enrollment (Global)* did not list a date but *Date of First Enrollment (India)* did; and (3) all 200 subjects were recruited from India. This appeared to be an Indian trial.

Of the remaining 21 Foreign trials, only three trials appeared to be truly foreign, since they had no recruitment from India, and other fields were also as expected for Foreign trials. Thus, for each of these three trials, (1) *Recruitment Status of Trial (Global)* was either “Completed” or “Not yet recruiting”, and *Recruitment Status of Trial (India)* was “Not applicable”; (2) a *Date of First Enrollment (Global)* was provided, whereas a *Date of First Enrollment (India)* was not; (3) *Total Sample Size* had a non-zero value, whereas *Sample Size from India* was nil.

For the remaining 18 trials, (1) in no case was *Recruitment Status of Trial (Global)* or *Recruitment Status of Trial (India)* listed as “Not applicable”; (2) all of them listed a date in *Date of First Enrollment (Global)*, and all but two listed one in *Date of First Enrollment (India)*; and (3) *Total Sample Size* ranged from 120 to 10,000 and the *Sample Size from India* ranged from 1 to 1000.

In order to ascertain whether, in fact, there were 2070 Indian trials and 640 Multinational trials, we proceeded to examine these three other pairs of fields for both those datasets as well. Details of each step of processing are available in Additional file 5. Only 1764 (85%) of the 2070 were unambiguously Indian trials, and only 609 (95%) of the 640 were unambiguously Multinational trials. Additional file 5 summarizes the processing of the Indian, Multinational, and Foreign trial data to determine the unambiguously correct cases in each of these three categories.

Quantification of problem over time: In Fig. 2a, Additional file 6 and Additional file 5, we quantify the problem of unambiguously identifying (1) the Indian trials, and (2) the Multinational trials. The percentages of trials with errors, over four time periods, were 63.1, 23.9, 4.4, and 3, respectively, for the Indian trials and 9.2, 5.1, 0.9, and 3.8, respectively, for the Multinational trials. As such, for the Indian trials, the error rates decreased 21-fold from time period one to four, and for the Multinational trials, they decreased 10-fold from time period one to three, but then increased again to 40% of the peak value.

Of these four fields, or pairs of fields, ClinicalTrials.gov only had *Country* (of recruitment). Therefore, for a global trial, there was no way to check the status of the trial in the USA or elsewhere.

### Relationship of Type of Trial and Phase of Trial

We went on to look at *Type of Trial*. The four options for this field were Observational, Interventional, PMS (that is, postmarketing surveillance), and BA/BE (Bioavailability/Bioequivalence). The 1764 Indian trials fell into either the Interventional (1655, or 94%) or PMS (109, or 6%) categories. Likewise, the 609 Multinational trials fell into either the Interventional (606, or 99.5%) or PMS (3, or 0.5%) categories. We proceeded to use the two sets of Interventional cases for all the analyses mentioned below, except where specified.

We first explored *Phase of Trial*. The options for this field were Phase 1, Phase 1/2, Phase 2, Phase 2/3, Phase 3, Phase 3/4, Phase 4, N/A, and PMS. For the Multinational set there were no cases of Phase listed as PMS, but for the Indian set there were 55 (3%) PMS cases (Additional file 5).

Quantification of problem over time: In Fig. 2a and Additional files 5 and 6, we quantify the problem of Interventional trials with *Phase* listed as PMS, for the Indian trials. The percentages of trials with errors, over four time periods, were 0, 0.7, 4.2, and 4.6, respectively. As such, the error rate increased from 0 to almost 5% over the four time periods.

In ClinicalTrials.gov, *Study type* had three options: Interventional studies, Observational studies (including Patient Registries), and Expanded Access studies. PMS was not an option, and therefore we could not compare this field in the two databases.

### Confusion between PMS and Phase 4 trials

Continuing from the preceding “Relationship of Type of Trial and Phase of Trial” section, we examined whether trials which listed PMS as the *Type of Trial* had Phase 4 as *Phase of Trial*, and identified such cases among the Indian, but not the Multinational trials.

Quantification of problem over time: In Fig. 2b and Additional files 5 and 6, we quantify the problem of the *Type of Trial* being PMS, but the *Phase* being Phase 4. This was done for a redefined set of Indian trials (wherein we started with the PMS trials rather than the Interventional trials), as detailed in Additional file 5. The percentages of trials with errors, over four time periods, were 100, 33.3, 22.5, and 37.9, respectively. As such, the error rates decreased more than fourfold, but then increased to 40% of the peak value.

As mentioned above, PMS was not an option in ClinicalTrials.gov, and therefore we could not compare this field in the two databases.

### Type of Trial: BA/BE versus Phases 1–4

Continuing with problems related to *Type of Trial*, we found that although BA/BE was a separate category, such trials were sometimes classified as having Phases 1, 1/2, 2, 2/3, 3, 3/4, or 4 (Additional file 5). Most of such cases were among the Indian trials.

Quantification of problem over time: In Fig. 2b and Additional files 5 and 6, we quantify the problem of the *Type of Trial* being BA/BE, but the *Phase* being 1, 1/2, 2, 2/3, 3, 3/4, or 4. This was done for a redefined set of Indian trials (wherein we started with the original set of 12,673 trials, used all the filters to generate the unambiguously Indian trials, and used the filter BA/BE for *Type of Trial*) as detailed in Additional file 5. The percentages of trials with errors, over four time periods, were 83.3, 19.6, 6.5, and 13.7, respectively. As such, for the Indian trials, the error rates decreased 13-fold over three time periods, but then increased again to almost 20% of the initial value.

In the Multinational set, there was just one trial each in Phases 1 and 2, so we could not investigate the error rates over time.

For ClinicalTrials.gov, there were three options in *Study type*: Interventional, Observational (subsection: Patient registries), and Expanded access. BA/BE was not an option, and therefore we could not compare this field in the two databases.

### Sites of study: incorrect listing of cities

In investigating the cities in which trials took place, we found some cases with incomplete or non-standard information.

Quantification of problem over time: In Fig. 2b and Additional files 5 and 6, we quantify the problem of the incorrect listing of cities in the Indian and the Multinational trials. The percentages of trials with errors, over four time periods, were 0, 2.5, 2.8, and 5.3, respectively for the Indian trials and 0.3, 1.3, 3.3, and 1.3, respectively for the Multinational trials. As such, for the Indian trials, the error rates increased from 0 to 5% from time period one to four, and for the Multinational trials, they increased 10-fold from time period one to three, but then decreased to 40% of the peak value in time period four.

It is not known how well the cities were classified in ClinicalTrials.gov.

### Missing data

Above, we noted that there was missing data in the section on *Countries of Recruitment*. We identified four additional fields for which there was missing data. These were (1) Name of Principal Investigator (PI), (2) Type of Study, (3) Name of Primary Sponsor, and (4) the state hosting a trial. We quantify these errors in the following sections.

#### Name of PI not listed

In examining the *Details of Principal Investigator or overall Trial Coordinator (multi-center study)* we found that for the Indian and Multinational cases, 5% and 40%, respectively, did not have any details in this field (Additional file 5).

Quantification of problem over time: In Fig. 2c and Additional files 5 and 6, we quantify the problem of the PI name not being listed for the Indian and Multinational trials. The percentages of trials with errors, over four time periods, were 10.3, 9, 4.4, and 3.5, respectively for the Indian trials and 54.1, 42.1, 32, and 33.1, respectively, for the Multinational trials. As such, for the Indian trials, the error rates decreased threefold from time period one to four, and for the Multinational trials, they decreased twofold from time period one to three, but then plateaued.

In earlier work, we found that in ClinicalTrials.gov, too, PI names were missing in many records, since it was a non-compulsory field [17].

#### Type of Study not listed

We identified trials that had no information for *Type of Study* but that listed Phases 1, 1/2, 2, 2/3, 3, 3/4, or 4. We identified such cases only among the Indian trials.

Quantification of problem over time: In Fig. 2c and Additional files 5 and 6, we quantify the problem of the *Type of Study* not being listed, but the *Phase* being 1, 1/2, 2, 2/3, 3, 3/4, or 4. This was done for a redefined set of Indian trials (wherein we started with the original set of 12,673 trials, used all the filters to generate the unambiguously Indian trials, and used the filter “Not available” for *Type of Study*) as detailed in Additional file 5. The percentages of trials with errors, over four time periods, were 87.5, 20.7, 6.2, and 13.4, respectively. As such, the error rates decreased 14-fold, but then doubled in time period four.

#### Name of Primary Sponsor not listed

We identified trials that did not mention the name of the *Primary Sponsor*. We identified such cases only among the Indian trials.

Quantification of problem over time: In Fig. 2c and Additional files 5 and 6, we quantify the problem of the *Primary Sponsor* not being named. The percentages of trials with errors, over four time periods, were 1.5, 2.7, 1.8, and 1, respectively. As such, the error rates almost doubled from time period one to two, but then dropped in the next two time periods to end up at 40% of the peak value.

#### The state hosting a trial not listed

We identified trials that did not list the state in which the trial took place.

Quantification of problem over time: In Fig. 2c and Additional files 5 and 6, we quantify the problem of the state hosting the trial not being listed, for the Indian and Multinational trials. The percentages of trials with error rates, over four time periods, were 5.9, 2.1, 0.2, and 0.1, respectively for the Indian trials and 12.5, 3.3, 1.3, and 0.1, respectively, for the Multinational trial. As such, for both the Indian and the Multinational trials, the low initial error rates dropped to almost nothing over time.

Many fields in ClinicalTrials.gov were compulsory, and it is therefore likely that the record of each trial was much more complete.

### Variations in a PI’s name

There were several possible variants of the name of a PI, which made it difficult to unambiguously establish that two names represented the same person, for instance. This became a particular challenge if automated methods were being used to process large numbers of names.

Examples of categories of these variations are listed below, where we have substituted the actual letters in names by the letters a, b, or c to protect the identity of the PI. CTRI records that illustrate these examples are listed in Additional file 5:

1. The presence or absence of the middle name (example Dr Aaaaa Bbbbb Ccccc and Dr Aaaaa Ccccc)
2. Parts of the name abbreviated (Dr Aaaaaaaa B Cccc and Dr A B Cccc)
3. Spelling mistakes (Dr Aaaaaa Bbbbbbb Cccccc and Dr Aaaaaa BbbbbbbbbCccccc)
4. Different ordering of parts of the name (DrAaaaaaaaaB and Dr B Aaaaaaaaa)
5. Different spacings in the name (Aaaaaa B C and Aaaaaa BC)
6. Variable use of capitals (Dr Aaaaaa Bbbbbbb and Dr AAAAAA BBBBBBB)
7. Extraneous information with the name (Aaaa Bbbbbb and Aaaa Bbbbbb MD).

In earlier work, we identified many such problems with the names of PIs in ClinicalTrials.gov as well [17].

### The name and classification of the Primary Sponsor

There were many cases of variations in the name of a given *Primary Sponsor*. Examples included the following variations for a given company: (1) Bristol Myers Squibb, BRISTOL MYERS SQUIBB, Bristol Myers Squibb India Pvt. Ltd., BristolMyers Squibb India pvt Ltd., and BristolMyers Squibb Research and Development; (2) Merck Sharp Dohme, Merck Sharp Dohme Corp, and Merck Sharp Dohme Corp a subsidiary of Merck Co Inc.; (3) Novo Nordisk India Private Limited, Novo Nordisk India Private Limited AS, and Novo Nordisk India Private Ltd.; and (4) Sanofi Synthelabo India Limited, SanofiSynthelabo IndiaLtd, and SanofiSynthelabo India Limited.

In ClinicalTrials.gov, the sponsor name seemed to have been chosen through a drop-down menu, since each organization appeared to be represented by just one version of a name. By way of examples, each of the following organizations was listed multiple times in the database in exactly the same manner: Acotec Scientific Co., Ltd.; Merck Sharp & Dohme Corp.; National Institute of Allergy and Infectious Diseases (NIAID); Albert Einstein College of Medicine; Baxter Healthcare Corporation; and Bausch & Lomb Incorporated.

Aside from variations in a given company’s name, we also noted variations in a given organization’s classification. For example, (1) each of the following companies was variably classified as Pharmaceutical industry-Global or Pharmaceutical industry-Indian in different trials: AstraZeneca, Boehringer Ingelheim, BristolMyers Squibb India Pvt. Ltd., and Eisai Limited; (2) Biogen Idec was classified as Other [Biotech Company], whereas Biogen Idec MA Inc. and Biogen Idec United Kingdom were classified as Pharmaceutical industry-Global; (3) Forest Research Institute Inc. was classified either as Research institution or as Pharmaceutical industry-Global; and (4) The National Institute of Allergy and Infectious Diseases of the National Institutes of Health, USA was classified either as a Government funding agency or as a Research institution and hospital.

For the classification of the *Primary Sponsor,* CTRI had quite a large number of categories, as follows: (1) Pharmaceutical industry-Global, (2) Pharmaceutical industry-Indian, (3) Contract research organization, (4) Government funding agency, (5) Research institution, (6) Research institution and hospital, and (7) Others. The following are examples of Others: Other [Healthcare industry], Other [international non-governmental and not-for-profit organization], Other [National public health institute of the United States], Other [Non profit organization works to improve health focused on Neglected Tropical Diseases], Other [Not for Profit Organisation], and so on.

In contrast to CTRI, the six organizations listed above as test cases appeared to be classified in ClinicalTrials.gov in one category each.

### Details of Ethics Committee

Next, we investigated *Details of Ethics Committee* and made several observations. These were (1) the lack of enough information to identify each ethics committee (EC) unambiguously; (2) lack of clarity on which site sought approval from which committee; (3) the listing of more ECs than sites of a given trial; and (4) the listing of foreign ECs along with Indian ones, for certain Multinational trials. For (1) and (2) we just identified a few examples, whereas for (3) and (4) we identified all the cases, and quantified the problem. Further details are provided in Additional file 5.

#### Lack of enough information to identify each EC unambiguously

All ECs did not have an address, or clear hospital affiliation, and may have been listed only by their names. As such, the affiliations and locations of such ECs could not always be established unambiguously. Examples of committee names were (1) Human welfare Ethics Committee for Human Sciences and Research; (2) Institutional Ethics Committee For Human Research; (3) Integrity Ethics Committee; (4) Regional Ethics Committee; and (5) LPR Ethics Committee.

#### Lack of clarity on which site sought approval from which committee

It was unclear which site sought approval from which EC. Multiple ECs may have approved a given trial, and if, for each site in *Sites of Study*, we looked for the corresponding institution or address in *Details of Ethics Committee*, we could not always infer which committee it was linked to.

#### The listing of more ECs than sites of a given trial

There were trials for which there were more ECs than sites. An example was one which had seven trial sites but 28 committees.

Quantification of problem over time: In Fig. 2a and Additional files 5 and 6, we quantify the problem of there being more ECs than trial sites in the Indian and Multinational trials. The percentages of trials with errors, over four time periods, were 9.4, 9.9, 4.6, and 9.5, respectively, for the Indian trials and 7.4, 6.6, 7.1, and 3.4, respectively, for the Multinational trials. As such, for the Indian trials, the error rate was close to 10% in all time periods except the third, when it halved. For the Multinational trials, it was around 7% in all time periods except the last, when it halved.

#### The listing of foreign ECs along with Indian ones

In the Multinational dataset there were two trial records in which foreign committees were included in the list of ECs. Examples of such committees included (1) Comite National D’Ethique pour la Recherche en Sante, Senegal; (2) Comite National d’Ethique et de Recherche (CNER) de Côte dIvoire; and (3) Convite nacional De Bioetica Para A Saude, Mozambique.

ClinicalTrials.gov did not have a field for EC approval, and therefore we could not compare this field in the two databases.

Finally, and aside from the findings listed above, we noted two challenges related to accessing data in the CTRI database, one concerned with the search function and the other with the download options. These are described in the following sections.

##### The search function of the database

The search function of the database did not work well, as illustrated by the following examples. (1) If, for *Type of Trial* we chose “Interventional”, 16 records were pulled up instead of thousands. (2) Likewise, if, for *Phase of Trial* we chose Phase 3, five records were pulled up instead of thousands. (3) Another example, concerning the search for trials run by one particular hospital, is detailed in Additional file 5. (4) If one wanted the list of all the trials hosted by the database, unless one entered the term “CTRI” as a keyword, no records were pulled up.

We did not carry out a systematic exploration of the search function of ClinicalTrials.gov.

##### Download options for trial data

It was not a straightforward task to download data related to a large number of trials at a time. The obvious option was to select individual trial records, open each in the browser, and download one HTML record at a time. Users with programming skills could use Python both as a web-scrapping bot as well as a parser to reformat the data from an unstructured, hard-to-query HTML format to a structured SQLite database.

At ClinicalTrials.gov, for up to 10,000 trials, up to 25 fields of information could be downloaded into a single file at the click of a button. This file could be in any of the following formats: comma-separated values, tab-separated values, plain text, PDF, or XML.

## Discussion

We first separately discuss each section whose results are presented above, before making more general comments. A summary of the problems identified as well as recommendations to improve CTRI records in the future are provided in Table 1.

**Table 1:** Various categories of problems with the data in CTRI, comparison with the relevant fields of ClinicalTrials.gov where possible, and suggestions to improve CTRI in future

No.	Category in paper	CTRI	ClinicalTrials.gov	Suggestion to improve CTRI in future
A. Lack of clarity in the classification of *Type of Study*				
1	1. *Type of Study*	We needed to consider two fields: 1. Under *Type of Trial*, there were four options, of which the registrant had to choose one from a drop-down menu: interventional, observational, BA/BE or postmarketing surveillance (PMS) 2. Under *Type of Study*, there were 18 fields, and any number could be chosen. In April 2018 we downloaded the data of all 12,673 trials. These trials fell into 1331 categories, which were rather confusing. Examples included: DrugSurgical/Anesthesia, DrugAyurveda,^a^ DrugSiddha, Drug Preventive, DrugBiological, DrugMedical Device, and DrugOther	There were two equivalent fields: 1. *Study type* had 3 options, Interventional studies, Observational studies (including Patient Registries), and Expanded Access studies 2. *Intervention* had 11 categories.^b^ One category could be chosen more than once, and more than one category could also be chosen. However the listing of multiple interventions was discrete and unambiguous, as follows: *Example 1*: Drug: 1.5 mg estradiol and 2.5 mg nomegestrol acetate|Drug: 15 μg ethinylestradiol and 60 μg gestodene *Example 2*: Radiation: [C-11]|Drug: NOP-1A	In order for registry users to rapidly understand the nature of a trial, registrants should classify each trial using a multi-layered menu of options, along the following lines: The first layer should be the system of medicine to which the trial belongs, and the options should be (1) allopathy (mainstream medicine) and (2) AYUSH^a^ (alternative medicine). Within each system, the choices for *Type of Trial* should remain as they are. Within interventional trials the choices should be prevention, screening, treatment, education, and others. Under treatment, a distinction should be made between small molecules, biologics, herbal remedies, or a combination thereof. Within biologics, a distinction should be made between vaccine, stem cell therapy, biological, or a combination thereof. Further details would need to be worked out to enable each trial to be optimally and comprehensively classified The output format must be unambiguous. A researcher should be able to select trials at any of these levels or those involving particular categories of interventions, for analysis
B. Internal inconsistencies				
2	2. *Countries of Recruitment*	There were four fields (or pairs of fields) which provided clues as to whether a trial was Indian, Multinational, or Foreign: (1) *Countries of Recruitment*, (2) *Recruitment Status of Trial (Global)* and *Recruitment Status of Trial (India)*, (3) *Date of First Enrollment (Global)* and *Date of First Enrollment (India)*, and (4) *Target SampleSize* and *Sample Size from India*. Data in these fields was sometimes inconsistent Illustratively, even if the *Country of Recruitment* was only India, there were sometimes inconsistent values in the other 3 pairs of fields, such as non-zero values in the two *Global* fields	Of these four fields (or pairs of fields) ClinicalTrials.gov only had *Country* (of recruitment). Therefore, for a global trial, there was no way to check the status of a trial in the USA versus in other countries	Logic rules must be implemented to prevent contradictory information being entered in the database. *Example 1*: If India is the only *Country of Recruitment*, then *Recruitment Status of Trial (Global)* and *Date of First Enrollment (Global)* must become inactive fields, and *Target Sample Size* and *Sample Size from India* must have the same values *Example 2*: If India is not a *Country of Recruitment*, then *Recruitment Status of Trial (India), Date of First Enrollment (India)*, and *Sample Size from India* must become inactive fields
3	3. Relationship of *Type of Trial* and *Phase of Trial*	For the field *Type of Trial*, there were four options: observational, interventional, PMS, and BA/BE. Although we only chose interventional studies, the Indian set had 55 (3%) cases that listed *Phase* as PMS	*Study type* had 3 options: Interventional studies, Observational studies (including Patient Registries), and Expanded Access studies. PMS was not an option, and therefore we could not compare this field in the two databases	Logic rules must be implemented so that if Interventional is chosen for *Type of Trial*, then the PMS option of *Phase of Trial* should become inactive, thereby preventing a *Phase* from being chosen
4	4. Confusion between PMS and Phase 4 trials	Trial registrants were sometimes confused about the terms PMS and Phase 4 trial. In these cases, PMS was chosen as the *Type of Trial* but the *Phase of Trial* as Phase 4	*Study type* had 3 options: Interventional studies, Observational studies (including Patient Registries), and Expanded Access studies. PMS was not an option, and therefore we could not compare this field in the two databases	Logic rules must be implemented so that if PMS is chosen for *Type of Trial*, then *Phase of Trial* should become inactive, thereby preventing a *Phase* from being chosen
5	5. *Type of Trial*: BA/BE versus Phase 1, 2, or 3	Although in *Type of Trial*, BA/BE was a separate category, such trials were sometimes classified as *Phase* 1, *Phase 2*, or *Phase 3* trials	There were 3 options in *Study type*: Interventional, Observational (subsection: Patient registries), and Expanded access. BA/BE was not an option, and therefore we could not compare this field in the two databases	Logic rules must be implemented so that if BA/BE is chosen for *Type of Trial*, then *Phase of Trial* should become inactive, thereby preventing a *Phase* from being chosen
C. Incomplete or non-standard information				
6	6. Sites of study: incorrect listing of cities	In investigating the cities in which trials took place, we found incomplete or non- standard information in some cases. It appears that this information was entered in a free text field	It is not known whether the cities were listed correctly	Instead of being entered in free text fields, cities should be selected from a drop-down menu to ensure standardization of information
D. Missing data				
7	7. Missing data	A few other fields were also found to have missing data: (1) Name of PI was nil (2) Trials in Phases 1, 1/2, 2, 2/3, 3, 3/4, or 4 that had no information for *Type of Study* (3) Name of Primary Sponsor was nil (4) The state hosting the trial was not listed	In earlier work [17], we found that PIs’ names were missing in many records, since it is a non-compulsory field. We have not done a systematic study of missing data in various fields	Most fields should be made compulsory, and unless data is entered in a particular format, sometimes from a drop-down menu, it should not be possible to register a trial. Ideally, the existing records should be updated
E. Variations in names or classification				
8	8. Variations in a PI’s name	There were variations in a given PI’s name, which sometimes made it difficult to unambiguously determine whether two names referred to the same person. Furthermore, two individuals may have shared a name	In earlier work [17], we identified 19 categories of variations in PIs’ names. Some examples of the types of variations are listed below^c^	Each PI’s name should be pre-registered, and thereafter, while registering the trial, the PI’s name should be chosen from a list through a drop-down menu. Since a person’s name may change over time, there should be a possibility to list the current name as well. Further, there should be a permanent and unique ID, such as an Open Researcher and Contributor ID (ORCID) number, linked to the name, with the system only accepting valid numbers
9	9. The name and classification of the Primary Sponsor	There were variations in a given organization’s name and classification *Name of sponsor:* For example, one company had the following name variants: BoehringerIngelheim India Pvt Ltd Boehringer Ingelheim India PvtLtd Boehringer Ingelheim India Private Limited *Classification of sponsor:* For example, one company may have been classified as “Pharmaceutical industry-Global” and as “Pharmaceutical industry-Indian” in different trials	*Name of sponsor:* The *Sponsor* name seemed to be chosen through a drop-down menu, since each organization appeared to be represented by just one version of a name. By way of examples, each of the following organizations were listed multiple times in exactly the same format: Acotec Scientific Co., Ltd; Merck Sharp & Dohme Corp.; National Institute of Allergy and Infectious Diseases (NIAID); Albert Einstein College of Medicine, Inc.; Bausch & Lomb Incorporated *Classification of sponsor:* There was no field for sponsor classification. The closest field was “Funder type”, for which there were four categories: (1) NIH, (2) Other US Federal agency, (3) Industry, and (4) All others (individuals, universities, organizations)	An organization’s name and its classification should be pre-registered, and thereafter, while registering the trial, the organization’s name should be chosen from a list through a drop-down menu Here, too, registrants should classify each trial using a multi-layered menu of options, along the following lines: The first layer should be a choice between Indian and foreign. The next should be between for-profit, not-for-profit, and government. The third layer should distinguish industry, hospital, research institution, university, charity, individual, and other, with these options being suitably available to the for-profit and not-for-profit categories, as applicable
F. Incomplete or incorrect details of Ethics Committees				
10	10. Details of Ethics Committees	Every ethics committee did not have an address or clear institutional affiliation and may have been listed only by its name. The identity of such committees could not always be established unambiguously	There was no field regarding ethics committee approval, and therefore we could not compare this field in the two databases	The following details of each ethics committee connected to a trial must be spelled out clearly, regardless of whether it is an institutional or an independent committee: Name, affiliation (if applicable), and address. Each of these subfields must be pre-registered, and subsequently chosen from a drop-down menu while registering the trial
11	10. Details of Ethics Committees	It was not always clear which site sought approval from which ethics committee	There was no field regarding ethics committee approval, and therefore we could not compare this field in the two databases	It must be possible to unambiguously identify which site sought approval from which ethics committee. Possibly the table listing the number of sites and the table listing the ethics committees could be merged
12	10. Details of Ethics Committees	There were examples of trials for which there were significantly more ethics committees than sites, such as 28 committees for 7 sites	There was no field regarding ethics committee approval, and therefore we could not compare this field in the two databases	If the table listing the number of sites and the table listing the ethics committees were merged, it would prevent such irrational entries
13	10. Details of Ethics Committees	There were examples of trial records in which foreign committees were included in the list of ethics committees, such as one in which 8 out of the 13 committees listed were foreign	There was no field regarding ethics committee approval, and therefore we could not compare this field in the two databases	Although the current guidelines for completing this field imply that only local ethics committees should be listed, this should be explicitly stated. Perhaps an information box should also reiterate this point while the trial is being registered. Also, if the table listing the number of sites and the table listing the ethics committees were merged, it would address this problem

^a^Ayurveda is a form of alternative medicine. AYUSH is an acronym for Ayurveda, Yoga, Unani, Siddha, and Homeopathy

^b^11 categories of interventions in ClinicalTrials.gov: Behavioral, Biological, Combination Product, Device, Diagnostic Test, Dietary Supplement, Drug, Genetic, Other, Procedure, and Radiation

^c^ (i)Extraneous information with the name, such as prefixes, suffixes, or punctuation marks

(ii) Variations in the name in the form of spelling mistakes, different ordering of parts of the name, abbreviations of parts of the name, parts of the name missing, etc.

(iii) Other variations such as a name represented by just one word, or two people sharing a name

### Type of Study

Under *Type of Study*, registrants had to choose from among 18 clearly defined categories, including “Others (please specify)”. However, the fact that the registrant could select multiple options led to the list of 1331 categories. This was confusing. It appears that the registry staff have not yet prioritized the simplification of the number of categories. In contrast, ClinicalTrials.gov had a much cleaner system.

In future versions of CTRI, the classification of *Type of Study* should be done using a multi-layered menu of options, along the following lines. The first layer should be the system of medicine to which the trial belongs, and the options should be allopathy and AYUSH, the acronym used in India to describe the alternate systems of medicine Ayurveda, Yoga, Unani, Siddha, and Homeopathy, with a possibility to choose either one or both of these systems of medicine for a given trial. The next layer should be *Type of Trial*, where the choices should remain as they currently are, that is (1) Observational, (2) Interventional, (3) PMS, and (4) BA/BE. Within Interventional trials, the choices should be prevention, screening, treatment, education, and others. Under treatment, a distinction should be made between small molecules, biologics, and herbal remedies, again with the possibility of choosing one or more of these options. Within biologics, the choices could be between vaccine, stem cell therapy, biological, or a combination thereof. Further details would need to be worked out to enable each trial to be optimally and comprehensively classified. Also, the output format must be unambiguous. This kind of hierarchy would ensure that the classification of a study would be more immediately informative than the current categories.

### Countries of Recruitment

Due to errors in filling fields, we concluded that, based on *Country of Recruitment*, of the 22 cases that we classified as Foreign only three were truly so. It was clear that for a correct assessment of whether or not a trial ran solely in India, for instance, it was insufficient to examine the *Country of Recruitment*. One also needed to examine (1) *Recruitment Status of Trial*, globally versus in India; (2) *Date of First Enrollment*, globally versus in India, and (3) *Total Sample Size* versus *Sample Size from India*.

In examining error rates over time, it was clear that either registrants have become more careful in providing this data, or registry staff have checked this field more carefully before accepting a trial.

In future versions of CTRI, logic rules must be implemented to prevent contradictory information being entered in the database. Examples of such rules are as follows. If India is the only *Country of Recruitment*, then (1) *Recruitment Status of Trial (Global)* and *Date of First Enrollment (Global)* must become inactive, and (2) *Total Sample Size* and *Sample Size from India* should only accept the same values. If India is not a *Country of Recruitment*, then *Recruitment Status of Trial (India), Date of First Enrollment (India)*, and *Sample Size from India* must all become inactive.

### Relationship of Type of Trial and Phase of Trial

Since we had rejected the PMS cases of *Type of Trial* and selected the 1655 Indian and 606 Multinational Interventional cases, no trials should have had PMS as Phase. However, we did find such cases among the Indian trials. In recent years, registrants have been less careful in providing this data while registering their trials, and registry staff appear not to have checked this field before accepting a trial.

In future versions of CTRI, if Interventional is chosen in *Type of Trial*, then the PMS option of *Phase of Trial* should become inactive.

### Confusion between PMS and Phase 4 trials

CTRI defined PMS and Phase 4 trials, respectively, as “Routine surveillance trials after marketing approval” and “Studies (other than routine surveillance) performed after drug is marketed and is related to the approved indication...”. As such, PMS and Phase 4 trials were mutually exclusive.

That is, if under *Type of Trial*, a study was selected as PMS, then it was not an Interventional trial and the Phase could not have been listed as 1, 1/2, 2, 2/3, 3, 3/4, or 4. Nevertheless, the registrants appear to have used the two terms interchangeably. Overall, it appears that in recent years, registrants have been increasingly careful in providing this data, or registry staff have checked this field more carefully before accepting a trial, although the recent increase in the error rate is concerning.

In future versions of CTRI, if PMS is chosen in *Type of Trial*, then *Phase of Trial* should become inactive.

### Type of Trial: BA/BE versus Phases 1–4

The *Type of Trial* field distinguished BA/BE trials from Interventional trials. Nevertheless, we identified BA/BE trials that listed a Phase.

Overall, it appears that registrants have been increasingly careful in providing this data, or registry staff have checked this field more carefully before accepting a trial, although the recent increase in the error rate is concerning.

In future versions of CTRI, logic rules must be implemented so that (1) if BA/BE is chosen as *Type of Trial*, then the *Phase* field should become inactive, and (2) if *Interventional* is chosen under *Type of Trial* and Phase 1 under *Phase of Trial*, there should be a reminder that BA/BE is a separate category under *Type of Trial*. In such cases, there should also be a separate box to tick, confirming that the study is not a BA/BE study. Further, if, in an Interventional trial, BA or BE is mentioned in *Public Title of Study* or *Scientific Title of Study*, then there should be an alert recommending correction of the *Type of Trial* to a BA/BE study, in case it holds true and is not already so.

### Sites of study: incorrect listing of cities

It has been a matter of concern as to whether trials in India adequately sampled the various ethnicities of the country [18]. City information is important to address this issue. Over time, registrants have generally been less careful in providing data on which cities were hosting trials while registering their trials, and registry staff appear not to have checked this field before accepting a trial.

Sometimes data was available upon inspection of a particular record but was incorrectly formatted and therefore was incorrectly captured in downloaded data. It appeared that city information was entered in a free text field. In future versions of CTRI, instead of being entered in free text fields, cities should be selected from a drop-down menu to ensure standardization of information.

### Missing data

Aside from the section on *Countries of Recruitment, w*e identified four other fields for which data was missing. These are discussed below.

#### Name of PI not listed

Regarding the problem of missing PI names, either recent registrants have been somewhat more careful in providing this data, or registry staff have checked this field more carefully before accepting a trial.

#### Type of Study not listed

Regarding the problem of the Type of Study not being listed, but the Phase being listed, it appears that recent registrants have been significantly more careful in providing this data, or registry staff have checked this field more carefully before accepting a trial. Nevertheless, the recent increase is concerning.

#### Name of Primary Sponsor not listed

Regarding the problem of the Primary Sponsor not being listed, either registrants have been more careful in providing this data, or registry staff have checked this field more thoroughly before accepting a trial.

#### The state hosting a trial not listed

Regarding the problem of the state hosting the trial not being listed, either registrants have been more careful in providing this data, or registry staff have checked this field more thoroughly before accepting a trial. Given the current low rates, it is possible that an automated method of collecting this data in a uniform format has been implemented.

Missing information is concerning. Data in clinical trial registries is often repurposed. In the past, registry data has been used to answer questions such as which organizations sponsor trials globally and where those sponsors are based [19], how the type of sponsor has changed over the years [20], why trials have been terminated prematurely [21], and so on. A PI plays a crucial role in the conduct of a trial, and questions such as (1) how many unique PIs there are, or (2) whether a given PI has been involved in too many trials at a given time, could be investigated if the PI names were correctly and comprehensively recorded. Likewise, data in the other fields would be useful for answering other questions. In future versions of CTRI, most fields should be made compulsory, and unless data is entered in a particular format, from a drop-down menu wherever feasible, it should not be possible to register the trial.

### Variations in a PI’s name

In future versions of CTRI, each PI’s name should be pre-registered and subsequently chosen from a drop-down menu while the trial is being registered. The name should also be linked to an Open Researcher and Contributor ID (ORCID) number or some other permanent ID which does not change even if the PI’s name changes. It should be possible to list the changed name of the PI as well. Further, only valid ID numbers and formats should be accepted by the system. These changes would enable automated methods to correctly count the number of unique PIs and the occurrence of each, for instance.

In earlier work we identified the same problem with data in ClinicalTrials.gov and made a similar recommendation for that database [17].

### The name and classification of the Primary Sponsor

In relation to the variations in a given Primary Sponsor’s name, we recommend that each name be pre-registered and subsequently chosen from a drop-down menu while a trial is being registered.

In relation to the variations in a given sponsor’s classification, it appears that the large number of categories contributed to the problem. ClinicalTrials.gov only had four categories of sponsors: the National Institutes of Health (NIH), Other US Federal agency, Industry, and All others (individuals, universities, organizations). This reduced the potential for confusion in classifying the sponsor. However, we do not argue for reducing the number of categories in CTRI, since more categories are more informative. In future versions of the database it would be better if registrants could classify the sponsor using a multi-layered menu of options along the following lines. The first layer should be a choice between Indian and foreign. The next should be between for-profit, not-for-profit, and government. The third layer should distinguish industry, hospital, research institution, university, charity, individual, and other, with these options being suitably available to the for-profit and not-for profit categories as applicable. Finally, an organization’s classification should also be pre-registered, and thereafter, while the trial is being registered, the classification should be chosen from a drop-down menu to ensure the standardization of information across trials.

### Details of Ethics Committee

We noted four problems with information in the *Details of Ethics Committee* field, two of which we quantified. Our recommendations to address the four issues are as follows.

#### Lack of enough information to identify each EC unambiguously

In future versions of CTRI, the name, affiliation (if any), and address of each EC connected to a trial must be spelled out clearly, regardless of whether it is an institutional or an independent committee. Each of these subfields must be pre-registered and subsequently chosen from a drop-down menu while the trial is being registered. As for the names of PIs, discussed previously, this would enable automated methods to correctly count the number of occurrences of each EC.

#### Lack of clarity on which site sought approval from which committee

In future versions of CTRI, it must be possible to unambiguously identify which site sought approval from which EC. Possibly the table listing the number of sites and the table listing the ethics committees could be merged.

#### The listing of more ECs than sites of a given trial

Regarding the problem of more ECs than trial sites, although overall it appears that registrants have been careful in providing this data, or registry staff have checked this field more carefully before accepting a trial, the recent increase in the error rate of the Indian trials is concerning.

It is highly unlikely that, in practice, several ECs were concerned with running a given trial at one site. In future versions of CTRI, there should be an automated mechanism to prevent such irrational entries. As suggested above, possibly the table listing the number of sites and the table listing the ethics committees could be merged.

#### The listing of foreign ECs along with Indian ones, for certain Multinational trials

Although the current guidelines for completing this field imply that only local ECs should be listed, this should be explicitly stated. Perhaps an information box should also reiterate this point while the trial is being registered. As discussed, possibly the table listing the number of sites and the table listing the ethics committees could be merged.

Other researchers have pointed out that not all ECs that approved studies were registered with the Central Drugs Standard Control Organization [15]. We did not examine this issue.

Although there have been clear transgressions of ethics in the West (such as the syphilis study involving African-American men in Tuskegee [22]), it was felt that, in general, Western nations had a good track record regarding the ethical conduct of trials. Therefore, for a long time, there was no requirement to list details of the ECs connected with a trial. However, the situation in India has been different. Ten years ago, it was reported that there was poor knowledge of where trials were taking place in the country and poor ethical oversight of the trials [2]. In response to this situation, an additional field, not then mandated by WHO, was included in the CTRI records, to ensure that the ECs associated with a given trial were listed. The ECs are required to approve a trial and also to monitor the research and assess serious adverse events. Although merely listing an EC does not guarantee proper ethical review of the proposed trial before approving it or the ethical conduct of the trial, such a listing is a small step to improve the ethical quality of trials. Thus, a major function of such a listing is to improve the accountability of an EC. Also, such a listing would enable the government or researchers to analyze how many trials each EC has approved. Should an audit of ECs be conducted, those that have approved a large number of trials should perhaps be prioritized for review. More recently, however, WHO has included a similar field in the WHO Trial Registration Data Set (Version 1.3.1) [23]. Entitled Ethics Review, it requires the following information to be listed: (1) “Status (possible values: Not approved, Approved, Not Available)”, (2) “Date of approval”, and (3) “Name and contact details of Ethics committee(s)”. We believe that “Details of Ethics committees” is an important field, and it should be retained in the CTRI records.

We now come to the two challenges related to accessing data in the CTRI database: the search function and the download options. (1) The search function needs to be thoroughly tested and its capabilities improved. (2) In future versions of CTRI, for as many records as the user wishes, all the fields and subfields of each trial should be downloadable into a single file (in different possible formats) at the click of a button. For the purpose of large-scale analysis, a CSV format must be an option. This would be similar to the option that was provided in an older version of ClinicalTrials.gov. Although the latter database currently restricts the bulk download of data to 10,000 trials at a time, prior to December 2017 there was no such limit, and this process could be applied to all the registered trials if required. For anyone wishing to do large-scale analyses of records in the database, this was a very convenient way to download information on all the trials registered with ClinicalTrials.gov.

## Summary

To summarize, CTRI is a much-needed database. It has helped improve the quality of reporting of trials, and trial methods have been better reported in CTRI than in Indian journal publications [24]. Nevertheless, we have discovered various categories of problems with the CTRI data, including:

1. Lack of clarity in the classification of *Types of Study*
2. Internal inconsistencies
3. Incomplete or non-standard information
4. Missing data
5. Variations in names or classification
6. Incomplete or incorrect details of ECs.

For the majority of problems that we have quantified, the error rates are in single digits. This is creditable. Nevertheless, there are fields which have significantly higher error rates. We have suggested the following ways in which these various categories of problems could be prevented in the future:

1. A more elaborate and structured way of classifying the *Type of Study*
2. The use of logic rules to prevent internal inconsistencies, as by the Australian New Zealand Clinical Trial Registry (ANZCTR) and ClinicalTrials.gov [25, 26]
3. Less use of free text fields and greater use of drop-down menus
4. More fields to be made compulsory, with data entry in a particular format
5. The pre-registration of individuals’ and organizations’ names, and their subsequent selection from drop-down menus while registering a trial
6. The pre-registration of an organization’s classification, and its subsequent selection from a drop-down menu while a trial is being registered
7. More information about each EC, including its affiliation and address, which should be pre-registered before registering a trial; and linking the name of the trial site to the relevant EC.

We wish to specifically highlight the issue of non-standard information (section Sites of study: Incorrect listing of cities). In such cases, the data is available upon inspection of a particular record, but is incorrectly formatted and therefore is incorrectly captured in downloaded data. Such data is not amenable to automated analysis when many records are analyzed at a time. Many such cases arise because data is entered in free text fields. As the number of registered trials increases, there will be heightened interest in performing landscape analyses of all the data in CTRI, as there has been for the data in ANZCTR [25, 27]. For landscape analyses, data entered via drop-down menus would be the most useful. The CTRI database was overhauled in 2011, a few years after its creation [10], and it may be overhauled in the future. Several of our suggestions, which supplement those made by others [13], are geared to such an event.

The administrators of ClinicalTrials.gov carried out a similar analysis of data integrity issues in that database some years ago [28] and found the following kinds of errors: (1) data appears invalid (where a value may be impossible), (2) non-meaningful information is provided (the information provided is too vague to make sense of), (3) a mismatch of the data (such as incorrect units), and (4) internal inconsistencies (such as an observational study with a trial design that includes randomization). Other researchers have also identified other shortcomings in ClinicalTrials.gov data: (1) observational trials labeled interventional [29], (2) trial sites not listed when the study starts or even after its completion [30], (3) discrepancies between the status of a trial in ClinicalTrials.gov and in the relevant publication [31], and (4) for a given trial, a discrepancy in registry information in ClinicalTrials.gov and the European Union Clinical Trials Register [32]. Although CTRI does not as yet host perfect records, the database is not unique in this regard.

For those who run registries, there is a huge effort involved in ensuring the quality of the database and of keeping it up to date [33]. Nevertheless, to truly fulfill the original purpose of establishing these registries, the managers of each one should aim for the data to be comprehensive and absolutely accurate, as has been reported for many fields of data of trials running in Australia [25] or New Zealand [27], and as has been proposed for the reporting of trial results [34].

On a separate note, we list some of the limitations of this study. First, we have analyzed only a subset of the trials registered with CTRI, and it is unclear whether the types of errors or their frequencies are similar in the rest of the trials. Second, we have not analyzed every field of this subset. As such, there are likely other problems with the hosted data that we have not identified. Third, we had no way to compare the information in the registry with the actual trial reports. Fourth, we were unable to (1) quantify some of the problems identified and (2) undertake a thorough comparison with ClinicalTrials.gov for every problem identified.

## Conclusions

We have discovered various categories of problems with the data in the CTRI database, including (1) a lack of clarity in the classification of *Types of Study*, (2) internal inconsistencies, (3) incomplete or non-standard information, (4) missing data, (5) variations in names or classification, and (6) incomplete or incorrect details of ECs. Where we have quantified these problems, the majority have error rates in single digits. This is creditable. Nevertheless, there are fields which have significantly higher error rates. We have suggested the following ways in which these various categories of problems could be prevented in the future: (1) use of a more elaborate and structured way of classifying the *Type of Study*, (2) the use of logic rules to prevent internal inconsistencies, (3) less use of free text fields and greater use of drop-down menus, (4) more fields to be made compulsory, with data entry in a particular format, (5) the pre-registration of individuals’ and organizations’ names, and their subsequent selection from drop-down menus while a trial is being registered, (6) the pre-registration of an organization’s classification, and its subsequent selection from a drop-down menu while a trial is registered, and (7) more information about each EC, including its affiliation and address, the pre-registration of its name and other details, and linking the name of the trial site to the relevant EC.

Clinical trial databases are prone to problems with the data. There are also commonalities in the types of problems found in different databases. CTRI is a valuable database, and the suggestions made herein would improve it further. Until that time, researchers using CTRI should be aware of some of the problems with the data.

## Additional files

Additional file 1: A sample CTRI record. (PDF 65 kb)

Additional file 2: The Python script used to extract data from CTRI to create the SQLite database. (DOC 80 kb)

Additional file 3: The SQLite database with details of 12,673 trial records from CTRI. (ZIP 20 mb)

Additional file 4: The schema of the SQLite database. (XLS 13 kb)

Additional file 5: Expanded methods, used to generate the data in the following sections. Sections 7–16 provide details on (7) The 1331 categories of Type of Study in 12,673 trials, year-wise from 2007–2018; (8) Determining the truly foreign trials; (9) Determining the unambiguously Indian trials, and error rates over time; (10) Determining the unambiguously Multinational trials, and error rates over time; (11) Identifying the actual trials in the categories Foreign, Indian and Multinational: A summary. (12) 55 Interventional Indian cases with Phase listed as PMS, and error rates over time; (13) For the redefined Indian and Multinational sets (i) cases of confusion between PMS and Phase 4 trials; (ii) cases where Type of Trial is BA/BE but Phase is 1–4; and (iii) Sites of study: Incorrect listing of cities. Error rates over time for some of these; (14) Missing data in terms of (i) Name of the PI was missing; (ii) for the redefined Indian set, and the Multinational set, cases where Type of Study was not available, but Phase of Trial was Phase 1, 1/2, 2, 2/3, 3, 3/4 or 4; (iii) Name of Primary Sponsor was missing; and (iv) the state hosting a trial was not listed. Error rates over time for some of these; (15) Examples of types of variations in PIs’ names; and examples, or the entire listing, of problems with ethics committees; and (16) A brief on the 47 trials conducted at the Malpani Multispeciality Hospital, Jaipur, Rajasthan. (ZIP 596 kb)

Additional file 6: The percentage of trials with errors, in 3-year time periods, for the several categories of errors. **a** The percentage of trials with errors, in 3-year time periods, for the following categories of errors: (i) *Type of Study*: Large number of categories, (ii) Errors in determining the unambiguously Indian trials, (iii) Errors in determining the unambiguously Multinational trials, (iv) Indian trials: Interventional cases with Phase listed as PMS, (v) Redefined Indian trials: *Type of Trial* was PMS, but *Phase of Trial* was Phase 4, and (vi) Redefined Indian trials: *Type of Trial* was BA/BE, but Phase was 1, 1/2, 2, 2/3, 3, 3/4, or 4. Columns of data using redefined datasets are *shaded*. **b** The percentage of trials with errors, in 3-year time periods, for the following categories of errors: (i) Indian trials: Incorrect listing of cities, (ii) Multinational trials: Incorrect listing of cities, (iii) Indian trials: PI names listed as null, (iv) Multinational trials: PI names listed as null, (v) Redefined Indian trials: *Type of Study* was NA, but Phase was 1, 1/2, 2, 2/3, 3, 3/4, or 4, and (vi) Indian trials: Primary sponsor name “nil”. Columns of data using redefined datasets are *shaded*. **c** The percentage of trials with errors, in 3-year time periods, for the following categories of errors: (i) Indian trials: State of trial site missing, (ii) Multinational trials: State of trial site missing, (iii) Indian trials: More ethics committees than trial sites, and (iv) Multinational trials: More ethics committees than trial sites. (XLS 16 kb)

## Data Availability

Additional files 2 and 3 are available from the Open Science Framework (OSF) database repository at https://osf.io/uh7j4/. The rest of the data generated or analyzed during this study are included in this published article and the other additional files.

## Abbreviations

ANZCTR: Australian New Zealand Clinical Trials Registry
BA/BE: Bioavailability/Bioequivalence
CTRI: Clinical Trials Registry - India
EC: Ethics committee
Foreign trials: Trials that took place only outside India
Indian trials: Trials that took place only in India
Multinational trials: Trials that took place in India and also in one or more other countries
PI: Principal Investigator
PMS: Postmarketing surveillance
USA: United States of America
WHO: World Health Organization
