# Supplementary material for: An analysis of deficiencies in the data of interventional drug trials registered with Clinical Trials Registry - India
Source: Trials. 2019 Aug 28;20:535. doi: 10.1186/s13063-019-3592-0 (PMC6712861; doi:10.1186/s13063-019-3592-0)
Supplement: Supplementary file 1 — A sample CTRI record. (PDF 65 kb) [file 13063_2019_3592_MOESM1_ESM.pdf]

Additional file 1. A sample CTRI record

Clinical Trial Details (PDF Generation Date :- Wed, 26 Jun 2019 04:18:33 GMT)

**CTRI Number**	CTRI/2018/02/011983 [Registered on: 19/02/2018] - **Trial Registered Prospectively**
**Last Modified On**	14/01/2019
**Post Graduate Thesis**	No
**Type of Trial**	Interventional
**Type of Study**	Drug
**Study Design**	Randomized, Parallel Group, Placebo Controlled Trial
**Public Title of Study**	Study of Baricitinib in adult patients with Atopic Dermatitis
**Scientific Title of Study**	Protocol I4V-MC-JAHL:A Multicenter, Randomized, Double-Blind,Placebo-Controlled, Phase 3 Study to Evaluate the Efficacy and Safety of Baricitinib in Adult Patients with Moderate to Severe Atopic Dermatitis

Secondary IDs if Any

Secondary ID	Identifier
Protocol I4V-MC-JAHL dated 29-Jun-2017	DCGI

Details of Principal Investigator or overall Trial Coordinator (multi-center study)

Details of Principal Investigator	
**Name**	Dr Tarun Puri
**Designation**	Medical Director
**Affiliation**	Eli Lilly and Company (India) Pvt. Ltd.
**Address**	Eli Lilly and Company (India) Pvt. Ltd. Plot No - 92, Sec - 32,Institutional Area, Gurgaon - 122001 HARYANA, India<br>Gurgaon<br>HARYANA<br>122001<br>India
**Phone**	
**Fax**	
**Email**	puri_tarun@lilly.com

Details Contact Person (Scientific Query)

Details Contact Person (Scientific Query)	
**Name**	Dr Tarun Puri
**Designation**	Medical Director
**Affiliation**	Eli Lilly and Company (India) Pvt. Ltd.
**Address**	Eli Lilly and Company (India) Pvt. Ltd. Plot No - 92, Sec - 32,Institutional Area, Gurgaon - 122001 HARYANA, India<br>Gurgaon<br>HARYANA<br>122001<br>India
**Phone**	
**Fax**	
**Email**	puri_tarun@lilly.com

Details Contact Person (Public Query)

Details Contact Person (Public Query)	
**Name**	Dr Rajeev Sharan Shrivastava
**Designation**	Associate Director - Clinical Research
**Affiliation**	Eli Lilly and Company (India) Pvt. Ltd.
**Address**	Eli Lilly and Company (India) Pvt. Ltd. Plot No - 92, Sec - 32,Institutional Area, Gurgaon - 122001 HARYANA, India<br>Gurgaon<br>HARYANA<br>122001<br>India

**Phone**	
**Fax**	
**Email**	shrivastavara@lilly.com

Source of Monetary or Material Support

**Source of Monetary or Material Support**
**>** Eli Lilly and Company (India) PVT LTD, Plot No. 92, Sector-32, Gurgaon, Haryana - 122001

Primary Sponsor

**Primary Sponsor Details**	
**Name**	Eli Lilly and Company India Pvt Ltd
**Address**	Eli Lilly and Company (India) Pvt Ltd, Plot No. 92, Sector-32, Gurgaon, Haryana - 122001
**Type of Sponsor**	Pharmaceutical industry-Global

Details of Secondary Sponsor

**Name**	**Address**
NIL	NIL

Countries of Recruitment

**List of Countries**
Canada
Czech Republic
France
Germany
India
Italy
Japan
Mexico
Russian Federation
Taiwan
United States of America

Sites of Study

**Name of Principal Investigator**	**Name of Site**	**Site Address**	**Phone/Fax/Email**
Dr Vinod Kumar Sharma	All India Institute of Medical Sciences (AIIMS)	Department of Dermatology and Venereology, All India Institute of Medical Sciences (AIIMS), Ansari Nagar East, New Delhi – 110029, India<br>New Delhi<br>DELHI	9871213543<br><br>vksiadvl@gmail.com
Dr Bela Jaswantlal Shah	B.J. Medical College and Civil Hospital	Department of Medicine, D4, Civil Hospital Campus, Asarwa Ahmedabad, Gujarat - 380 016 India<br>Ahmadabad<br>GUJARAT	9898059289<br><br>shah.drbela@gmail.com
Dr Jayaraaman Ammani Murugaiya	Chennai Meenakshi Multispecialty Hospital	New No. 72, Old No. 149 , Luz Church Road, Myla pore, Chennai - 600 004. Tamil Nadu, INDIA<br>Chennai<br>TAMIL NADU	9444119274<br><br>jayaraamana@gmail.com
Dr Kiran Vasant Godse	D .Y. Patil Hospital & Research Centre	Sectore – 5, Nerul, Navi Mumbai, Maharashtra - 400706 India	9322266687<br><br>drgodse@gmail.com

		Mumbai<br>MAHARASHTRA	
Dr Gurram Narsimha Rao Netha	Gandhi Hospital	Dept of Dermatology, 3rd floor, 6-1-34, Musheerabad Secunderabad, Andhra Pradesh 500048, India<br>Hyderabad<br>ANDHRA PRADESH	9390032196<br><br>gnrnetha@gmail.com
Dr Yogesh Shashikant Marfatia	Government Medical College & Sir Sayajirao General Hospital (SSGH)	Jail road, Indira Avenue, Sayajiganj, Vadodara Vadodara, Gujarat 390001 India<br>Vadodara<br>GUJARAT	9825917442<br><br>ym11256@gmail.com
Dr Pradyumna Prakash Vaidya	Jehangir Clinical Development Centre	Jehangir Hospital Premises, 32, Sasson Road, Near Pune Railway Station, Bund Garden Road, Sangamvadi, Pune, Maharashtra – 411001, India<br>Pune<br>MAHARASHTRA	9822400964<br><br>drpvaidya@gmail.com
Dr Venkatachalam Konakanchi	King George Hospital and Andhra Medical College	Dept of Dermatology, Maharani Peta, Vizag, Andhra Pradesh – 530002, India<br>Visakhapatnam<br>ANDHRA PRADESH	9848398923<br><br>drkvchalam99@gmail.com
Dr T K Sumathy	M S Ramaiah Medical College & Hospital	MSRIT Post, New BEL Road Bangalore, Karnataka 560054 India<br>Bangalore<br>KARNATAKA	9845163009<br><br>tksumathy@gmail.com
Dr Rajeev Agarwal	M.V. Hospital & Research Centre	314/30, Mirza Mandi, Chowk, Lucknow – 226003, Uttar Pradesh, India<br>Lucknow<br>UTTAR PRADESH	9415080604<br><br>drrajeeva1@rediffmail.com
Dr Hemangi Rajiv Jerajani	Mahatma Gandhi Mission Medical College	MGM Campus, Plot No. 1 & 2, Sector-18, At NH-4 Junction, Mumbai-Pune Express Way, Kamothe, Navi Mumbai, Maharashtra 410209 India<br>Mumbai<br>MAHARASHTRA	9820031483<br><br>jerajani@rediffmail.com
Dr Kirti Prakash Shah	Panchshil Hospital	2nd Floor, Ramnagar, Near Sabarmati Police Station Highway, Ahmedabad, Gujarat - 380005 India<br>Ahmadabad<br>GUJARAT	9825651751<br><br>drkirtipshah@yahoo.in
Dr Parag Gopal Kalyani	Ruby Hall Clinic	40, Sassoon Road	266455495

		Pune, Maharashtra - 411001 India<br>Pune<br>MAHARASHTRA	parag.kalyani@yahoo.com
Dr Uday Sharadchandra Khopkar	Seth G. S. Medical College & K. E. M Hospital	Department of Dermatology, 2nd floor, Old hospital building, Acharya Donde Marg, Parel Mumbai, Maharashtra 400012 India<br>Mumbai<br>MAHARASHTRA	9322671959<br><br>drkhopkar@gmail.com
Dr Rohit Batra	Sir Gangaram Hospital	Department of Dermatology,Rajinder Nagar. New Delhi-110060, India<br>New Delhi<br>DELHI	9911300050<br><br>drrohitbatra@gmail.com

Details of Ethics Committee

Name of Committee	Approval Status	Date of Approval	Is Independent Ethics Committee?
Chennai Meenakshi Multispeciality Hospital Ethics Committee	Submittted/Under Review	No Date Specified	No
Ethics Committee , Jehangir Clinical Development Centre Pvt. Ltd	Submittted/Under Review	No Date Specified	No
Ethics Committee, M.S. Ramaiah Medical College and Hospitals	Submittted/Under Review	No Date Specified	No
Ethics Committee, MGM Institute of Health Sciences	Submittted/Under Review	No Date Specified	No
Ethics Committee, Sir Ganga Ram Hospital	Submittted/Under Review	No Date Specified	No
Institute Ethics Committee, All India Institute of Medical Sciences	Approved	10/12/2018	No
Institutional Ethics Committee for Human Research, Medical College	Submittted/Under Review	No Date Specified	No
Institutional Ethics Committee, Dr D Y Patil Medical College	Approved	05/11/2018	No
Institutional Ethics Committee, King George Hospital	Approved	22/01/2018	No
Institutional Ethics Committee, M.V. Hospital	Approved	28/10/2018	No
Institutional Ethics Committee, Poona Medical Research Foundation	Submittted/Under Review	No Date Specified	No

Institutional Ethics Committee, Principle office, Gandhi Medical College	Submittted/Under Review	No Date Specified	No
Institutional Ethics Committee-I, Seth G. S. Medical College & K. E. M. Hospital	Submittted/Under Review	No Date Specified	No
Panchshil Institutional Ethics Committee	Approved	06/02/2018	No
The Institutional Ethics Committee, B J Medical College & Civil Hospital	Submittted/Under Review	No Date Specified	No

Regulatory Clearance Status from DCGI

Status	Date
Approved/Obtained	12/02/2018

Health Condition / Problems Studied

Health Type	Condition
Patients	Adult Patients with Moderate to Severe Atopic Dermatitis
Patients	Atopic dermatitis, unspecified

Intervention / Comparator Agent

Type	Name	Details
Intervention	Baricitinib 1-mg once daily (QD), 2-mg QD, and 4-mg QD	Study I4V-MC-JAHL (JAHL) is a Phase 3, multicenter, randomized, double-blind, placebo-controlled, parallel-group, outpatient study evaluating the efficacy and safety of baricitinib 1-mg once daily (QD), 2-mg QD, and 4-mg QD as compared to placebo.Approximately 600 patients ?18 years of age who have responded inadequately to or who are intolerant to topical therapy will be randomized at a 2:1:1:1 ratio to receive placebo QD, baricitinib 1-mg QD, baricitinib 2-mg QD, or baricitinib 4-mg QD (240 patients in the placebo group; 120 patients in each baricitinib treatment group).
Comparator Agent	Placebo	Patients who meet all criteria for enrollment will be randomized in a 2:1:1:1 ratio (placebo, baricitinib 1-mg; baricitinib 2-mg; baricitinib 4-mg) to double-blind treatment at Visit 2 (Week 0). Randomization will be stratified by geographic region (North America [NA], Europe [EU], Japan [JPN], rest-of-world [ROW]) and disease severity at baseline (IGA 3 vs. 4).

Inclusion Criteria

Inclusion Criteria	
**Age From**	18.00 Year(s)
**Age To**	60.00 Year(s)

**Gender**	Both
**Details**	[1] are at least 18 years of age at the time of informed consent.<br>[2] are able to read, understand, and give documented (electronic or paper signature) informed consent.<br>[3] have a diagnosis of AD at least 12 months prior to screening, as defined by the<br>American Academy of Dermatology: Guidelines of care for the management<br>of AD; Section 1. Diagnosis and assessment of atopic dermatitis<br>[4] have moderate to severe AD, including all of the following:<br>a. Eczema Area and Severity Index (EASI) score ?16 at screening (Visit 1)<br>and at randomization (Visit 2)<br>b. IGA score of ?3 at screening (Visit 1) and at randomization (Visit 2)<br>c. ?10% of BSA involvement at screening (Visit 1) and at randomization(Visit 2).<br>[5] have a documented history by a physician and/or investigator of inadequate response to existing topical medications within 6 months preceding screening,or history of intolerance to topical therapy as defined by at least 1 of the<br>following:<br>a. inability to achieve good disease control defined as mild disease or better(e.g., IGA ?2) after use of at least a medium potency TCS for at least 4 weeks, or for the maximum duration recommended by the product prescribing information (e.g., 14 days for super-potent TCS), whichever is shorter. Topical corticosteroids may be used with or without TCNIs.<br>b. Patients who failed systemic therapies intended to treat AD within 6 months preceding screening, such as cyclosporine, methotrexate, azathioprine, mycophenolate mofetil will also be considered as a surrogate for having inadequate response to topical therapy.<br>c. documented history of clinically significant adverse reactions with the use of TCS such as skin atrophy, allergic reactions, systemic effects that in the opinion of the investigator outweigh the benefits of retreatment.<br>[6] agree to discontinue use of the following excluded medications/treatments for<br>at least 4 weeks prior to randomization (Visit 2) and throughout the study:<br>a. systemic corticosteroids and leukotriene inhibitors<br>b. systemic immunomodulators, including, but not limited to, cyclosporine,methotrexate, mycophenolate mofetil, and azathioprine<br>c. sedating antihistamines, including, but not limited to, alimemazine, chlorphenamine, clemastine, cyproheptadine, diphenhydramine, hydroxyzine, ketotifen, and promethazine<br>Note: Patients may use newer, less sedating antihistamines (e.g.,fexofenadine, loratadine, cetirizine).<br>d. any other systemic therapy used to treat AD or symptoms of AD(approved or off-label use)<br>e. phototherapy, includes therapeutic phototherapy (psoralen plus ultraviolet-A, ultraviolet-B), excimer laser as well as self-treatment with tanning beds.<br>[7] agree to discontinue use of the following excluded medications for at least 2 weeks prior to randomization (Visit 2) and throughout the study:<br>a. TCS or topical immune modulators (e.g., tacrolimus or pimecrolimus)<br>b. Topical phosphodiesterase type 4 (PDE-4) inhibitor (crisaborole)<br>c. Topical JAK inhibitor (e.g., tofacitinib or ruxolitinib) and/or any other

	investigative topical treatments.<br>[8] have applied emollients daily for at least 14 days prior to randomization and agree to use emollient daily throughout the treatment period.<br>[9] Patients who are receiving chronic treatments to improve sleep should be on a stable dose for at least 2 weeks prior to screening as determined by the investigator. Sedating antihistamines (see above) are not permitted.<br>[10] are male or nonpregnant, nonbreastfeeding female patients,except:<br>a. Male patients must agree to use 2 forms of birth control (1 must be highly<br>effective, see below) while engaging in sexual intercourse with female<br>partners of childbearing potential while enrolled in the study and for at<br>least 4 weeks following the last dose of investigational product.<br>b. Female patients of childbearing potential must agree to use 2 forms of birth control, when engaging in sexual intercourse with a male partner<br>while enrolled in the study and for at least 4 weeks following the last dose of investigational product.

Exclusion Criteria

Exclusion Criteria	
**Details**	[1] are currently experiencing or have a history of other concomitant skin conditions (e.g., psoriasis or lupus erythematosus) that would interfere with evaluations of the effect of study medication on AD.<br>[2] patients who, in the opinion of the investigator, are currently experiencing or<br>have a history of erythrodermic, refractory, or unstable skin disease that requires frequent hospitalizations and/or intravenous treatment for skin infections that may interfere with participation in the study.<br>[3] a history of eczema herpeticum within 12 months prior to screening.<br>[4] a history of 2 or more episodes of eczema herpeticum in the past.<br>[5] patients who are currently experiencing a skin infection that requires treatment, or is currently being treated, with topical or systemic antibiotics.<br>[6] have any serious concomitant illness that is anticipated to require the use of systemic corticosteroids or otherwise interfere with study participation or require active frequent monitoring (e.g., unstable chronic asthma).<br>[7] have been treated with the following therapies:<br>a. monoclonal antibody (e.g., ustekinumab, omalizumab, dupilumab) for less than 5 half-lives prior to randomization.<br>b. received prior treatment with any oral JAK inhibitor (e.g., tofacitinib,ruxolitinib)<br>c. received any parenteral corticosteroid administered by intramuscular or<br>intravenous injection within 2 weeks prior to study entry (Visit 1) or within 6 weeks prior to planned randomization (Visit 2) or are anticipated<br>to require parenteral injection of corticosteroids during the study.<br>d. have had an intra-articular corticosteroid injection within 2 weeks prior to<br>study entry (Visit 1) or within 6 weeks prior to planned randomization (Visit 2).<br>[8] are largely or wholly incapacitated permitting little or no self-care, such as

being bedridden.
[9] have uncontrolled arterial hypertension characterized by a repeated systolic
blood pressure >160 mm Hg or diastolic blood pressure >100 mm Hg in a seated position.
[10] have had any major surgery within 8 weeks prior to screening or will require
major surgery during the study that, in the opinion of the investigator in
consultation with Lilly or its designee, would pose an unacceptable risk to the
patient.
[11] are immunocompromised and, in the opinion of the investigator, at an unacceptable risk for participating in the study.
[12] have experienced any of the following within 12 weeks of screening: venous
thromboembolic event (VTE), myocardial infarction (MI), unstable ischemic heart disease, stroke, or New York Heart Association Stage III/IV heart
failure.
[13] have a history of recurrent (? 2) VTE or are considered at high risk of VTE as deemed by the investigator.
[14] have a history or presence of cardiovascular, respiratory, hepatic,
gastrointestinal, endocrine, hematological, neurological, or neuropsychiatric
disorders or any other serious and/or unstable illness that, in the opinion of the
investigator, could constitute an unacceptable risk when taking investigational product or interfere with the interpretation of data.
[15] have a history of lymphoproliferative disease; or have signs or symptoms
suggestive of possible lymphoproliferative disease, including lymphadenopathy or splenomegaly; or have active primary or recurrent
malignant disease; or have been in remission from clinically significant malignancy for less than 5 years.
a. Patients with cervical carcinoma in situ that has been resected with no
evidence of recurrence or metastatic disease for at least 3 years may participate in the study.
b. Patients with basal cell or squamous epithelial skin cancers that have been
completely resected with no evidence of recurrence for at least 3 years
may participate in the study.
[16] have a current or recent clinically serious viral, bacterial, fungal, or parasitic
infection, including but not limited to the following:

a. symptomatic herpes zoster infection within 12 weeks prior to screening.
b. a history of disseminated/complicated herpes zoster (e.g., multidermatomal involvement, ophthalmic zoster, CNS involvement, or
post-herpetic neuralgia).
c. symptomatic herpes simplex at the time of randomization.
d. active or chronic viral infection from hepatitis B virus (HBV), hepatitis C
virus (HCV), or human immunodeficiency virus (HIV).
e. household contact with a person with active tuberculosis (TB) and

	did not<br>receive appropriate and documented prophylaxis for TB.<br>f. evidence of active TB or have previously had evidence of active TB and<br>did not receive appropriate and documented treatment.<br>g. clinically serious infection or received intravenous antibiotics for an infection, within the past 4 weeks of randomization.<br>h. any other active or recent infection within 4 weeks of randomization that,<br>in the opinion of the investigator, would pose an unacceptable risk to the<br>patient if participating in the study.<br>[17] have been exposed to a live vaccine within 12 weeks prior to planned randomization or are expected to need/receive a live vaccine during the course of the study (with the exception of herpes zoster vaccination).<br>[18] have a history of chronic alcohol abuse, IV drug abuse, or other illicit drug<br>abuse within the 2 years prior to screening.<br>[19] presence of significant uncontrolled neuropsychiatric disorder, are clinically<br>judged by the investigator to be at risk for suicide, or have a “yes” answer to<br>any of the following:<br>a. Question 4 (Active Suicidal Ideation with Some Intent to Act, Without<br>Specific Plan) on the “Suicidal Ideation” portion of the Columbia Suicide<br>Severity Rating Scale (C-SSRS) or<br>b. Question 5 (Active Suicidal Ideation with Specific Plan and Intent) on the<br>“Suicidal Ideation” portion of the C-SSRS or<br>c. Any of the suicide-related behaviors (actual attempt, interrupted attempt,<br>aborted attempt, preparatory act or behavior) on the “Suicidal Behavior”<br>portion of the C-SSRS;and the ideation or behavior occurred within 2 months prior to Visit 1.<br>[20] have donated more than a single unit of blood within 4 weeks prior to screening or intend to donate blood during the course of the study.<br>[21] are unable or unwilling to make themselves available for the duration of the<br>study and/or are unwilling to follow study restrictions/procedures, including use of data collection devices.<br>[22] are currently enrolled in any other clinical trial involving an investigational product or any other type of medical research judged not to be scientifically or medically compatible with this study.<br>[23] have participated within the last 30 days in a clinical study involving an investigational product. If the previous investigational product has a long half-life (2 weeks or longer), at least 3 months or 5 half-lives (whichever is<br>longer) should have passed.<br>[24] have previously been randomized in this study or any other study investigating baricitinib.<br>[25] are investigator site personnel directly affiliated with this study and/or their<br>immediate families. Immediate family is defined as a spouse, parent, child, or<br>sibling, whether biological or legally adopted.<br>[26] are Lilly or Incyte employees or their designee.

**Method of Generating Random Sequence**	Stratified randomization
**Method of Concealment**	Centralized
**Blinding/Masking**	Participant and Investigator Blinded

Primary Outcome

Outcome	Timepoints
To test the hypothesis that baricitinib 4-mg QD is superior to placebo in the treatment of patients with<br>moderate to severe AD.	Proportion of patients achieving IGA of 0 or 1 with<br>a ?2-point improvement at Week 16.

Secondary Outcome

Outcome	Timepoints
To test the hypothesis that baricitinib 2-mg QD or baricitinib 1-mg QD is superior to placebo in the treatment of patients with moderate to severe AD	Proportion of patients achieving IGA of 0 or 1 with<br>a ?2-point improvement at Week 16.
To compare the efficacy of baricitinib 1-mg QD, 2-mg QD, or 4-mg QD to placebo in AD during the<br>16-week double-blind placebo-controlled treatment<br>period as measured by improvement in signs and<br>symptoms of AD.	1. Poportion of patients achieving EASI75 at<br>16 weeks<br>2. Proportion of patients achieving EASI90 at<br>16 weeks<br>3. Percent change from baseline in EASI score at<br>16 weeks.<br>4. Proportion of patients achieving SCORAD75 at<br>16 weeks.
To compare the efficacy of baricitinib 1-mg QD, 2-mg QD, or 4-mg QD to placebo in AD during the<br>16-week double-blind placebo-controlled treatment<br>period as assessed by patient-reported outcome measures.	1.Proportions of patients achieving a 4-point improvement in Itch NRS at 1 week, 2 weeks,4 weeks, and 16 weeks<br>2.Mean change from baseline in the score of Item 2<br>of the ADSS at 1 week and 16 weeks<br>3.Mean change from baseline in Skin Pain NRS at 16 weeks.

**Target Sample Size**	**Total Sample Size=**600<br>**Sample Size from India=**60<br>**Final Enrollment numbers achieved (Total)=**Applicable only for Completed/Terminated trials<br>**Final Enrollment numbers achieved (India)=**Applicable only for Completed/Terminated trials
**Phase of Trial**	Phase 3
**Date of First Enrollment (India)**	15/03/2018
**Date of First Enrollment (Global)**	23/11/2017
**Estimated Duration of Trial**	**Years=**1<br>**Months=**6<br>**Days=**0
**Recruitment Status of Trial (Global)**	Open to Recruitment
**Recruitment Status of Trial (India)**	Open to Recruitment
**Publication Details**	Nomura and Kabashima 2015 Genovese et al. 2016; Dougados et al. 2017; Fleischmann et al. 2017; Taylor et al. 2017 Papp et al. 2016);
**Brief Summary**	Atopic dermatitis (AD) is a pruritic, chronic or chronically relapsing, highly symptomatic inflammatory skin disease characterized by excessive T cell activation leading to significant skin infiltration by T cells and dendritic cells (Bieber 2010). Presentation is varied, but includes skin manifestations and pruritus, with associated sleep disturbances and subsequent skin infections. The course of disease includes relapses of varying duration and severity.<br>Baricitinib is an orally available, selective Janus kinase (JAK) inhibitor with potency and selectivity for JAK1 and JAK2 and less potency for JAK3 or tyrosine kinase 2 (TYK2) (Fridman et al. 2010). The pathogenesis of AD is thought to be modulated through thymic stromal lymphopoietin (TSLP), interleukin (IL)-13, IL-5, IL-22, and IL-31, many of which activate receptors with downstream signaling through intracellular JAK1/JAK2/TYK2 (Nomura and Kabashima 2015). This activity profile suggests that baricitinib would inhibit cytokines involved in AD pathogenesis.<br>Clinical studies have established that baricitinib is effective in autoimmune/autoinflammatory diseases involving the joints, kidneys, and skin. Baricitinib was effective at reducing swollen and tender joints in patients with rheumatoid

arthritis (Genovese et al. 2016; Dougados et al. 2017; Fleischmann et al. 2017; Taylor et al. 2017); was effective at reducing disease severity in patients with moderate to severe plaque psoriasis (Papp et al. 2016); was effective at reducing the urinary albumin-to-creatinine ratio in patients with diabetic kidney disease (Tuttle et al. 2015); and in a recently completed Phase 2 study (I4V-MC-JAHG) was effective at reducing disease severity in patients with moderate to severe AD. The mechanism of action, combined with demonstration of clinical benefit in inflammatory diseases involving joints, kidneys, and skin, provides the rationale for evaluating baricitinib in moderate to severe AD.
