# Supplementary material for: An analysis of deficiencies in the data of interventional drug trials registered with Clinical Trials Registry - India
Source: Trials. 2019 Aug 28;20:535. doi: 10.1186/s13063-019-3592-0 (PMC6712861; doi:10.1186/s13063-019-3592-0)
Supplement: Supplementary file 2 — The Python script used to extract data from CTRI to create the SQLite database. (DOC 80 kb) [file 13063_2019_3592_MOESM2_ESM.doc]

import requests

import pandas as pd

from bs4 import BeautifulSoup

from tabulate import tabulate

import sys

reload(sys)

sys.setdefaultencoding("ISO-8859-1")

from tabulate import tabulate

import sqlite3

conn = sqlite3.connect('ctri.db')

conn.text_factory = str

c=conn.cursor()

f2 = open("success.txt",'w')

f3 = open("failure.txt",'w')

#Create table

try:

c.execute('''CREATE TABLE BasicDetails(ctri_number text, primarykey text, status_of_reg text, year_of_reg text, last_modified_on text,\

post_graduate_thesis text, type_of_trial text, type_of_study text, study_design text, public_title_of_study text,\

scientific_title_of_study text,secondary_id text, identifier text, source_of_monetary text, countries_of_recruitment text,\

regulatory_clearance_status text, phase_of_trial text, date_of_first_enrollment_india text, date_of_study_completion_india text,\

date_of_first_enrollment_global text,date_of_study_completion_global text, \

recruitment_status_of_trial_global text,recruitment_status_of_trial_india text, noofsites int, noofethicalcommittees text,\

final_enrollment_numbers_total text, final_enrollment_numbers_india text, total_sample_size text, sample_size_from_india text,\

method_of_generating_random_sequence text, method_of_concealment text, blinding_masking text, primary_outcome_details text, primary_outcome_timeline text,\

secondary_outcome_details text, secondary_outcome_timeline text, brief_summary text,\

PRIMARY KEY (primarykey,ctri_number))''')

except:

pass

try:

c.execute('''CREATE TABLE PrincipalInvestigatorDetails(ctri_number text, primarykey text,\

name text, designation text, email text, fax text, phone text, address text,\

city text, state text, pin text, country text, affiliation text, PRIMARY KEY (primarykey,ctri_number))''')

except:

pass

try:

c.execute('''CREATE TABLE ContactScientificQuery(ctri_number text, primarykey text,\

name text, designation text, email text, fax text, phone text, address text,\

city text, state text, pin text, country text, affiliation text, PRIMARY KEY (primarykey,ctri_number))''')

except:

pass

try:

c.execute('''CREATE TABLE ContactPublicQuery(ctri_number text, primarykey text,\

name text, designation text, email text, fax text, phone text, address text,\

city text, state text, pin text, country text, affiliation text, PRIMARY KEY (primarykey,ctri_number))''')

except:

pass

try:

c.execute('''CREATE TABLE PrimarySponsor(ctri_number text, primarykey text,\

name text, typeofsponsor text, address text, PRIMARY KEY (primarykey,ctri_number))''')

except:

pass

try:

c.execute('''CREATE TABLE SitesOfStudy(ctri_number text, primarykey text,\

site text, address text, \

state text, Name text, city text)''')

except:

pass

try:

c.execute('''CREATE TABLE EthicalCommittee(ctri_number text, primarykey text,\

status text, Name text)''')

except:

pass

try:

c.execute('''CREATE TABLE HealthCondition(ctri_number text, primarykey text,\

health_type text, condition text)''')

except:

pass

try:

c.execute('''CREATE TABLE InclusionExclusion(ctri_number text, primarykey text,\

inclusion_age_from text, inclusion_age_to text, inclusion_gender text,\

inclusion_details text, exclusion text, PRIMARY KEY (primarykey,ctri_number))''')

except:

pass

FinalDictionary={}

htmlpage = "page1.html"

f1=open(htmlpage,'r')

lines = f1.readlines()

entries=[]

for i in range(len(lines)):

if lines[i].find("pmaindet2.php?trialid=")!=-1:

sp = lines[i].split("(")

sp1 = sp[1].split("&")

entry = "http://ctri.nic.in/Clinicaltrials/%s"%sp1[0][1:]

entries.append(entry)

for z in range(len(entries)):

singleentrytest = entries[z]

singleentrytest = 'http://ctri.nic.in/Clinicaltrials/pmaindet2.php?trialid=386'

print "%s. %s"%(str(z+1),singleentrytest)

try:

res= requests.get("%s"%singleentrytest)

soup = BeautifulSoup(res.content,'lxml')

#find all tables in the HTML file

interflag = 1

tds = soup.find_all("td")

#certain things can be stored directly since the table seems standard

#could be wrong though will have to see for the time being thoug

forctrinumber = tds[3].text.decode('utf-8','ignore').encode("utf-8").strip().split('[')

#print tds[3].text.decode('utf-8','ignore').encode("utf-8").strip()

try:

statusofreg = forctrinumber[1].split(']').strip()[1]

originalctri = forctrinumber[0]

except:

statusofreg = 'NA'

originalctri = forctrinumber[0]

try:

yearofregisteration = forctrinumber[0].split('/')[1]

#registeredon = forctrinumber[1].strip().split('Registered on:')

except:

yearofregisteration = forctrinumber.split('/')[1]

try:

typestudy = forstudytype[1].strip()

except:

typestudy = 'NA'

primaryid = originalctri.replace('/','')

FinalDictionary

FinalDictionary['ctri_number'] = originalctri

FinalDictionary['primarykey'] = primaryid

FinalDictionary['status_of_reg'] = typestudy

FinalDictionary['year_of_reg'] = yearofregisteration

if tds[4].text.strip().find("Last Modified On:")!=-1:

if len(tds[5].text.strip())>0:

FinalDictionary["last_modified_on"] = tds[5].text.strip()

else:

FinalDictionary["last_modified_on"] = "NA"

if tds[6].text.strip().find("Post Graduate Thesis")!=-1:

if len(tds[7].text.strip())>0:

FinalDictionary["post_graduate_thesis"] = tds[7].text.strip()

else:

FinalDictionary["post_graduate_thesis"] = "NA"

if tds[8].text.strip().find("Type of Trial")!=-1:

if len(tds[9].text.strip())>0:

FinalDictionary["type_of_trial"] = tds[9].text.strip()

else:

FinalDictionary["type_of_trial"] = "NA"

if tds[10].text.strip().find("Type of Study")!=-1:

if len(tds[11].text.strip())>0:

FinalDictionary["type_of_study"] = tds[11].text.strip()

else:

FinalDictionary["type_of_study"] = "NA"

if tds[12].text.strip().find("Study Design")!=-1:

if len(tds[13].text.decode('utf-8','ignore').encode("utf-8").strip())>0:

FinalDictionary["study_design"] = tds[13].text.strip()

else:

FinalDictionary["study_design"] = "NA"

if tds[14].text.strip().find("Public Title of Study")!=-1:

if len(tds[15].text.strip())>0:

FinalDictionary["public_title_of_study"] = tds[15].text.strip()

else:

FinalDictionary["public_title_of_study"] = "NA"

if tds[16].text.strip().find("Scientific Title of Study")!=-1:

if len(tds[17].text.strip())>0:

FinalDictionary["scientific_title_of_study"] = tds[17].text.strip()

else:

FinalDictionary["scientific_title_of_study"] = "NA"

for i in range(18,len(tds)):

if tds[i].text.strip()=="Secondary ID":

FinalDictionary['secondary_id'] = tds[i+2].text.decode('utf-8','ignore').encode("utf-8").strip()

FinalDictionary['identifier'] = tds[i+3].text.decode('utf-8','ignore').encode("utf-8").strip()

if tds[i].text.strip().find("Details of Principal Investigator or overall Trial Coordinator") !=-1:

togetpi = tds[i+1]

pidict = {}

pi = togetpi.findAll('td')

address = str(pi[7]).replace('<br/>','|').replace('<td>','').replace('</td>','')

foradd = address.split('||')

try:

completeaddress = foradd[0]

specifics = foradd[1].split('|')

except:

completeaddress = 'NA'

pass

try:

city = specifics[0].decode('utf-8','ignore').encode("utf-8").strip()

except:

city = 'NA'

try:

state = specifics[1].decode('utf-8','ignore').encode("utf-8").strip()

except:

state = 'NA'

try:

pin = specifics[2].decode('utf-8','ignore').encode("utf-8").strip()

except:

pin = 'NA'

try:

country = specifics[3].replace('\xc2\xa0','').strip()

except:

country = 'NA'

pidict['address'] = completeaddress.replace('\r\n','')

pidict['city'] = city

pidict['state'] = state

pidict['pin'] = pin

pidict['country'] = country

pidict[pi[0].text.decode('utf-8','ignore').encode("utf-8").strip()] = pi[1].text.decode('utf-8','ignore').encode("utf-8").strip()

pidict[pi[2].text.decode('utf-8','ignore').encode("utf-8")] = pi[3].text.decode('utf-8','ignore').encode("utf-8").strip()

pidict[pi[4].text.decode('utf-8','ignore').encode("utf-8")] = pi[5].text.decode('utf-8','ignore').encode("utf-8").strip()

pidict[pi[8].text.decode('utf-8','ignore').encode("utf-8")] = pi[9].text.decode('utf-8','ignore').encode("utf-8").strip()

pidict[pi[10].text.decode('utf-8','ignore').encode("utf-8")] = pi[11].text.decode('utf-8','ignore').encode("utf-8").strip()

pidict[pi[12].text.decode('utf-8','ignore').encode("utf-8")] = pi[13].text.decode('utf-8','ignore').encode("utf-8").strip()

FinalDictionary['principal_investigator_details'] = pidict

if tds[i].text.strip().find("Details of Contact")!=-1:

flag = 1

if flag == 1:

togetContact = tds[i+1]

contactdict ={}

contact = togetContact.findAll('td')

address = str(contact[7]).replace('<br/>','|').replace('<td>','').replace('</td>','')

foradd = address.split('||')

try:

completeaddress = foradd[0]

specifics = foradd[1].split('|')

except:

completeaddress = 'NA'

pass

try:

city = specifics[0].replace('\xc2\xa0','').strip()

except:

city = 'NA'

try:

state = specifics[1].replace('\xc2\xa0','').strip()

except:

state = 'NA'

try:

pin = specifics[2].replace('\xc2\xa0','').strip()

except:

pin = 'NA'

try:

country = specifics[3].replace('\xc2\xa0','').strip()

except:

country = 'NA'

contactdict['address'] = completeaddress.replace('\r\n','')

contactdict['city'] = city

contactdict['state'] = state

contactdict['pin'] = pin

contactdict['country'] = country

try:

contactdict[contact[0].text.decode('utf-8','ignore').encode("utf-8").strip()] = contact[1].text.replace('\xc2\xa0','').strip()

except:

contactdict[contact[0].text.decode('utf-8','ignore').encode("utf-8").strip()] = 'NA'

try:

contactdict[contact[2].text.decode('utf-8','ignore').encode("utf-8").strip()] = contact[3].text.replace('\xc2\xa0','').strip()

except:

contactdict[contact[2].text.decode('utf-8','ignore').encode("utf-8").strip()] = 'NA'

try:

contactdict[contact[4].text.decode('utf-8','ignore').encode("utf-8").strip()] = contact[5].text.replace('\xc2\xa0','').strip()

except:

contactdict[contact[4].text.decode('utf-8','ignore').encode("utf-8").strip()] = 'NA'

#contactdict[contact[6].text.decode('utf-8','ignore').encode("utf-8").strip()] = contact[7].text.decode('utf-8','ignore').encode("utf-8").strip()

try:

contactdict[contact[8].text.decode('utf-8','ignore').encode("utf-8").strip()] = contact[9].text.replace('\xc2\xa0','').strip()

except:

contactdict[contact[8].text.decode('utf-8','ignore').encode("utf-8").strip()] = 'NA'

try:

contactdict[contact[10].text.decode('utf-8','ignore').encode("utf-8").strip()] = contact[11].text.replace('\xc2\xa0','').strip()

except:

contactdict[contact[10].text.decode('utf-8','ignore').encode("utf-8").strip()] = 'NA'

try:

contactdict[contact[12].text.decode('utf-8','ignore').encode("utf-8").strip()] = contact[13].text.replace('\xc2\xa0','').strip()

except:

contactdict[contact[12].text.decode('utf-8','ignore').encode("utf-8").strip()] = 'NA'

FinalDictionary['contact_scientific_query'] = contactdict

flag = flag+1

if flag == 2:

togetContact = tds[i+1]

contactdict ={}

contact = togetContact.findAll('td')

address = str(contact[7]).replace('<br/>','|').replace('<td>','').replace('</td>','')

foradd = address.split('||')

try:

completeaddress = foradd[0]

specifics = foradd[1].split('|')

except:

completeaddress = 'NA'

pass

try:

city = specifics[0].decode('utf-8','ignore').encode("utf-8").strip()

except:

city = 'NA'

try:

state = specifics[1].decode('utf-8','ignore').encode("utf-8").strip()

except:

state = 'NA'

try:

pin = specifics[2].decode('utf-8','ignore').encode("utf-8").strip()

except:

pin = 'NA'

try:

country = specifics[3].replace('\xc2\xa0','').strip()

except:

country = 'NA'

contactdict['address'] = completeaddress.replace('\r\n','')

contactdict['city'] = city

contactdict['state'] = state

contactdict['pin'] = pin

contactdict['country'] = country

try:

contactdict[contact[0].text.decode('utf-8','ignore').encode("utf-8").strip()] = contact[1].text.replace('\xc2\xa0','').strip()

except:

contactdict[contact[0].text.decode('utf-8','ignore').encode("utf-8").strip()] = 'NA'

try:

contactdict[contact[2].text.decode('utf-8','ignore').encode("utf-8").strip()] = contact[3].text.replace('\xc2\xa0','').strip()

except:

contactdict[contact[2].text.decode('utf-8','ignore').encode("utf-8").strip()] = 'NA'

try:

contactdict[contact[4].text.decode('utf-8','ignore').encode("utf-8").strip()] = contact[5].text.replace('\xc2\xa0','').strip()

except:

contactdict[contact[4].text.decode('utf-8','ignore').encode("utf-8").strip()] = 'NA'

#contactdict[contact[6].text.decode('utf-8','ignore').encode("utf-8").strip()] = contact[7].text.decode('utf-8','ignore').encode("utf-8").strip()

try:

contactdict[contact[8].text.decode('utf-8','ignore').encode("utf-8").strip()] = contact[9].text.replace('\xc2\xa0','').strip()

except:

contactdict[contact[8].text.decode('utf-8','ignore').encode("utf-8").strip()] = 'NA'

try:

contactdict[contact[10].text.decode('utf-8','ignore').encode("utf-8").strip()] = contact[11].text.replace('\xc2\xa0','').strip()

except:

contactdict[contact[10].text.decode('utf-8','ignore').encode("utf-8").strip()] = 'NA'

try:

contactdict[contact[12].text.decode('utf-8','ignore').encode("utf-8").strip()] = contact[13].text.replace('\xc2\xa0','').strip()

except:

contactdict[contact[12].text.decode('utf-8','ignore').encode("utf-8").strip()] = 'NA'

FinalDictionary['contact_public_query'] = contactdict

flag = 1

if tds[i].text.strip().find("Source of Monetary")!=-1:

try:

FinalDictionary['source_of_monetary'] = tds[i+1].text.strip()

except:

FinalDictionary['source_of_monetary'] = "NA"

if tds[i].text.strip().find("Primary Sponsor")!=-1:

sponsor = tds[i+1].findAll('td')

sponsordict ={}

try:

sponsordict['name'] = sponsor[1].text.strip()

except:

sponsordict['name'] = 'NA'

try:

sponsordict['address'] = sponsor[3].text.strip()

except:

sponsordict['address'] = 'NA'

try:

sponsordict['type_of_sponsor'] = sponsor[5].text.strip()

except:

sponsordict['type_of_sponsor'] = 'NA'

FinalDictionary['primary_sponsor'] = sponsordict

if tds[i].text.strip().find("Details of Secondary")!=-1:

secsponsor = tds[i+1].findAll('td')

secsponsordict = {}

try:

secsponsordict['name']=secsponsor[1].text.strip()

except:

secsponsordict['name']='NA'

try:

secsponsordict['address']=secsponsor[3].text.strip()

except:

secsponsordict['address']='NA'

FinalDictionary['secondary_sponsor'] = secsponsordict

if tds[i].text.strip().find("Countries of Recruitment")!=-1:

countries = str(tds[i+1]).strip().replace('<br/>','|').replace('<td>','').replace('</td>','').replace(' ','').replace('\xc2\xa0','').replace('\n','')

spcountries = countries.strip().split('|')

try:

if len(spcountries)>1:

places = ','.join([str(o) for o in spcountries])

else:

places = countries

except:

places = "NA"

#print tds[i+1].text.decode('utf-8','ignore').encode("utf-8").strip()

FinalDictionary['countries_of_recruitment']=places.strip()

if tds[i].text.strip().find("Sites of Study")!=-1:

siteofstudydict={}

sss = tds[i+1].findAll('td')

noofsites = sss[0].text.strip().split('=')[1].strip()

siteofstudydict['no_of_sites'] = noofsites

#print noofsites

#print len(sss)

lols = lambda lst, sz: [lst[i:i+sz] for i in range(0, len(lst), sz)] #make a list of lists

newsss = sss[5:]

sss1 = lols(newsss,4)

for m in range(len(sss1)):

tempdict={}

name = sss1[m][0].text.replace('\xa0','')

site = sss1[m][1].text.replace('\xa0','')

siteaddress = str(sss1[m][2]).replace('<br/>','|').replace('<td>','').replace('</td>','')

contact = sss1[m][3].text

foraddress = siteaddress.split('|')

try:

genaddress = foraddress[0]

except:

genaddress = "NA"

try:

city = foraddress[1]

except:

city = 'NA'

try:

state = foraddress[2]

except:

state = "NA"

tempdict['Name'] = name.decode('utf-8','ignore').encode("utf-8").strip()

tempdict['site'] = site

tempdict['address'] = genaddress.decode('utf-8','ignore').encode("utf-8").strip().replace('\r\n','')

tempdict['city'] = city.replace('\xc2\xa0','').strip()

tempdict['state'] = state.replace('\xc2\xa0','').strip()

siteofstudydict[str(m)] = tempdict

FinalDictionary['sites_of_study'] = siteofstudydict

if tds[i].text.strip().find("Details of Ethics Committee")!=-1:

ethicalcommittee = tds[i+1].findAll('td')

ethicalcommitteedict = {}

noofcommittees = ethicalcommittee[0].text.strip().split('=')[1].strip()

ethicalcommitteedict['no_of_committees'] = noofcommittees

lols = lambda lst, sz: [lst[i:i+sz] for i in range(0, len(lst), sz)] #make a list of lists

newethicalcommittee = ethicalcommittee[3:]

ethc = lols(newethicalcommittee,2)

for l in range(len(ethc)):

tempethc={}

tempethc["Name"] = ethc[l][0].text.replace('\xc2\xa0','').strip()

tempethc["status"] = ethc[l][1].text.replace('\xc2\xa0','').strip()

ethicalcommitteedict[str(l)] = tempethc

FinalDictionary['ethical_committee'] = ethicalcommitteedict

if tds[i].text.strip().find("Regulatory Clearance")!=-1:

FinalDictionary['regulatory_clearance_status'] = tds[i+3].text.strip()

if tds[i].text.strip().find("Health Condition")!=-1:

FinalDictionary['health_condition']={'health_type':tds[i+4].text.replace('\xc2\xa0','').strip(),'condition':tds[i+5].text.replace('\xc2\xa0','').strip()}

if tds[i].text.strip().find("Intervention")!=-1:

if interflag == 1:

interventioncomp = {}

inter = tds[i+1].findAll('td')

try:

interventioncomp['comparator_agent'] = inter[4].text.strip()

except:

interventioncomp['comparator_agent'] = 'NA'

try:

interventioncomp['details_comparator'] = inter[5].text.strip()

except:

interventioncomp['details_comparator'] = 'NA'

try:

interventioncomp['intervention'] = inter[7].text.strip()

except:

interventioncomp['intervention'] = 'NA'

try:

interventioncomp['details_intervention'] = inter[8].text.strip()

except:

interventioncomp['details_intervention'] = 'NA'

FinalDictionary['intervention_comparator_agent'] = interventioncomp

interflag = 0

if tds[i].text.strip().find("Inclusion")!=-1:

inclusion={}

incl = tds[i+1].findAll('td')

try:

agefrom = incl[1].text.replace('\xa0',' ').strip()

except:

agefrom = 'NA'

try:

ageto = incl[3].text.replace('\xa0',' ').strip()

except:

ageto = 'NA'

try:

gender = incl[5].text.replace('\xa0',' ').strip()

except:

gender = 'NA'

try:

details = incl[7].text.replace('\xa0',' ').strip()

except:

details = 'NA'

inclusion['age_from'] = agefrom

inclusion['age_to'] = ageto

inclusion['gender'] = gender

inclusion['details'] = details

FinalDictionary['inclusion_criterion'] = inclusion

if tds[i].text.strip().find("ExclusionCriteria")!=-1:

FinalDictionary['exclusion_criterion'] = tds[i+3].text.strip()

if tds[i].text.strip().find("Method of Generating")!=-1:

FinalDictionary['method_of_generating_random_sequence'] = tds[i+1].text.decode('utf-8','ignore').encode("utf-8").strip()

FinalDictionary['method_of_concealment'] = tds[i+3].text.decode('utf-8','ignore').encode("utf-8").strip()

FinalDictionary['blinding_masking'] = tds[i+5].text.decode('utf-8','ignore').encode("utf-8").strip()

try:

FinalDictionary['primary_outcome'] = {'outcome': tds[i+10].text.replace('\xc2\xa0','').strip(), 'timepoints': tds[i+11].text.replace('\xc2\xa0','').strip()}

except:

FinalDictionary['primary_outcome'] = {'outcome':'NA', 'timepoints':'NA'}

try:

FinalDictionary['secondary_outcome'] = {'outcome': tds[i+16].text.replace('\xc2\xa0','').strip(), 'timepoints': tds[i+17].text.replace('\xc2\xa0','').strip()}

except:

FinalDictionary['secondary_outcome'] = {'outcome':'NA', 'timepoints':'NA'}

if tds[i].text.strip().find("Brief Summary")!=-1:

try:

FinalDictionary['brief_summary'] = tds[i+1].text.replace('\xc2\xa0','').strip()

except:

FinalDictionary['brief_summary'] = "NA"

if tds[i].text.strip().find("Target Sample")!=-1:

ts = str(tds[i+1]).strip().replace('<br/>','|').replace('<td>','').replace('</td>','').replace('<b>','').replace('</b>','')

ts1 = ts.split('|')

tssa = ts1[0].split("=")[1].strip().replace('\xc2\xa0','')

tssb = ts1[1].split("=")[1].strip().replace('\xc2\xa0','')

tssc = ts1[2].split("=")[1].strip().replace('\xc2\xa0','')

tssd = ts1[3].split("=")[1].strip().replace('\xc2\xa0','')

FinalDictionary['target_sample_size'] = {'total_sample_size':tssa,'sample_size_from_india':tssb,'final_enrollment_numbers_total':tssc, 'final_enrollment_numbers_india':tssd}

FinalDictionary['phase_of_trial'] = tds[i+3].text.decode('utf-8','ignore').encode("utf-8").strip()

FinalDictionary['date_of_first_enrollment_india'] = tds[i+5].text.strip()

FinalDictionary['date_of_study_completion_india'] = tds[i+7].text.strip()

FinalDictionary['date_of_first_enrollment_global'] = tds[i+9].text.strip()

FinalDictionary['date_of_study_completion_global'] = str(tds[i+11].text.decode('utf-8','ignore').encode("utf-8").strip())

dur = str(tds[i+13]).replace('<td>','').replace('</td>','').replace('</b>','').replace('<b>','').replace('<br/>','|').strip().split('|')

#time = (((int(dur[0].split("=")[1][1:-1])*12)+(int(dur[1].split("=")[1][1:-1])))*30)+(int(dur[2].split("=")[1][1:-1]))

year = dur[0].split("=")[1][1:-1]

month = dur[1].split("=")[1][1:-1]

days = dur[2].split("=")[1][1:-3]

try:

durationoftrialindays = (int(year)*12 + int(month))*30+int(days)

except:

durationoftrialindays = 0

FinalDictionary['estimated_duration_of_trial'] = {'years':year, 'months':month, 'days':days}

FinalDictionary['estimated_duration_of_trial_indays'] = durationoftrialindays

FinalDictionary['recruitment_status_of_trial_global'] = tds[i+15].text.decode('utf-8','ignore').encode("utf-8").strip()

FinalDictionary['recruitment_status_of_trial_india'] = tds[i+17].text.decode('utf-8','ignore').encode("utf-8").strip()

c.execute('INSERT INTO BasicDetails VALUES(?,?,?,?,?,?,?,?,?,?,?,?,?,?,?,?,?,?,?,?,?,?,?,?,?,?,?,?,?,?,?,?,?,?,?,?,?)',\

(FinalDictionary['ctri_number'],FinalDictionary['primarykey'],FinalDictionary['status_of_reg'],\

FinalDictionary['year_of_reg'],FinalDictionary['last_modified_on'],FinalDictionary['post_graduate_thesis'],\

FinalDictionary['type_of_trial'],FinalDictionary['type_of_study'],FinalDictionary['study_design'],\

FinalDictionary['public_title_of_study'],FinalDictionary['scientific_title_of_study'],\

FinalDictionary['identifier'],FinalDictionary['secondary_id'],FinalDictionary['source_of_monetary'],\

FinalDictionary['countries_of_recruitment'],FinalDictionary['regulatory_clearance_status'],FinalDictionary['phase_of_trial'],\

FinalDictionary['date_of_first_enrollment_india'],FinalDictionary['date_of_study_completion_india'],\

FinalDictionary['date_of_first_enrollment_global'],FinalDictionary['date_of_study_completion_global'],\

FinalDictionary['recruitment_status_of_trial_global'],\

FinalDictionary['recruitment_status_of_trial_india'],\

FinalDictionary['sites_of_study']['no_of_sites'], FinalDictionary['ethical_committee']['no_of_committees'],\

FinalDictionary['target_sample_size']['final_enrollment_numbers_total'], FinalDictionary['target_sample_size']['final_enrollment_numbers_india'],\

FinalDictionary['target_sample_size']['total_sample_size'], FinalDictionary['target_sample_size']['sample_size_from_india'],\

FinalDictionary['method_of_generating_random_sequence'], FinalDictionary['method_of_concealment'], FinalDictionary['blinding_masking'],\

FinalDictionary['primary_outcome']['outcome'],FinalDictionary['primary_outcome']['timepoints'],\

FinalDictionary['secondary_outcome']['outcome'], FinalDictionary['secondary_outcome']['timepoints'], FinalDictionary['brief_summary']))

conn.commit()

c.execute('INSERT INTO PrincipalInvestigatorDetails VALUES(?,?,?,?,?,?,?,?,?,?,?,?,?)',\

(FinalDictionary['ctri_number'],FinalDictionary['primarykey'], FinalDictionary['principal_investigator_details']['Name'], FinalDictionary['principal_investigator_details']['Designation'],\

FinalDictionary['principal_investigator_details']['Email'], FinalDictionary['principal_investigator_details']['Fax'],\

FinalDictionary['principal_investigator_details']['Phone'],FinalDictionary['principal_investigator_details']['address'],\

FinalDictionary['principal_investigator_details']['city'],FinalDictionary['principal_investigator_details']['state'],\

FinalDictionary['principal_investigator_details']['pin'],FinalDictionary['principal_investigator_details']['country'],\

FinalDictionary['principal_investigator_details']['Affiliation']))

conn.commit()

c.execute('INSERT INTO ContactScientificQuery VALUES(?,?,?,?,?,?,?,?,?,?,?,?,?)',\

(FinalDictionary['ctri_number'],FinalDictionary['primarykey'], FinalDictionary['contact_scientific_query']['Name'], FinalDictionary['contact_scientific_query']['Designation'],\

FinalDictionary['contact_scientific_query']['Email'], FinalDictionary['contact_scientific_query']['Fax'],\

FinalDictionary['contact_scientific_query']['Phone'],FinalDictionary['contact_scientific_query']['address'],\

FinalDictionary['contact_scientific_query']['city'],FinalDictionary['contact_scientific_query']['state'],\

FinalDictionary['contact_scientific_query']['pin'],FinalDictionary['contact_scientific_query']['country'],\

FinalDictionary['contact_scientific_query']['Affiliation']))

conn.commit()

c.execute('INSERT INTO ContactPublicQuery VALUES(?,?,?,?,?,?,?,?,?,?,?,?,?)',\

(FinalDictionary['ctri_number'],FinalDictionary['primarykey'], FinalDictionary['contact_public_query']['Name'], FinalDictionary['contact_public_query']['Designation'],\

FinalDictionary['contact_public_query']['Email'], FinalDictionary['contact_public_query']['Fax'],\

FinalDictionary['contact_public_query']['Phone'],FinalDictionary['contact_public_query']['address'],\

FinalDictionary['contact_public_query']['city'],FinalDictionary['contact_public_query']['state'],\

FinalDictionary['contact_public_query']['pin'],FinalDictionary['contact_public_query']['country'],\

FinalDictionary['contact_public_query']['Affiliation']))

conn.commit()

c.execute('INSERT INTO PrimarySponsor VALUES(?,?,?,?,?)',\

(FinalDictionary['ctri_number'],FinalDictionary['primarykey'],\

FinalDictionary['primary_sponsor']['Name'], FinalDictionary['primary_sponsor']['Type of Sponsor'],\

FinalDictionary['primary_sponsor']['Address']))

conn.commit()

subdict1 = FinalDictionary['sites_of_study']

subdict1.pop('no_of_sites')

subdict1keys = subdict1.keys()

for s1 in range(len(subdict1keys)):

subdict2 = subdict1[subdict1keys[s1]]

c.execute('INSERT INTO SitesOfStudy VALUES(?,?,?,?,?,?,?)',\

(FinalDictionary['ctri_number'],FinalDictionary['primarykey'],\

subdict2['site'],subdict2['address'],subdict2['state'], subdict2['Name'],\

subdict2['city']))

conn.commit()

subdictE = FinalDictionary['ethical_committee']

subdictE.pop('no_of_committees')

subdictEkeys = subdictE.keys()

for s2 in range(len(subdictEkeys)):

subdictE2 = subdictE[subdictEkeys[s2]]

c.execute('INSERT INTO EthicalCommittee VALUES(?,?,?,?)',\

(FinalDictionary['ctri_number'],FinalDictionary['primarykey'],\

subdictE2['status'],subdictE2['Name']))

conn.commit()

c.execute('INSERT INTO HealthCondition VALUES(?,?,?,?)',\

(FinalDictionary['ctri_number'],FinalDictionary['primarykey'],\

FinalDictionary['health_condition']['health_type'], FinalDictionary['health_condition']['condition']))

conn.commit()

c.execute('INSERT INTO InclusionExclusion VALUES(?,?,?,?,?,?,?)',\

(FinalDictionary['ctri_number'],FinalDictionary['primarykey'],\

FinalDictionary['inclusion_criterion']['age_from'], FinalDictionary['inclusion_criterion']['age_to'],\

FinalDictionary['inclusion_criterion']['gender'], FinalDictionary['inclusion_criterion']['details'],\

FinalDictionary['exclusion_criterion']))

conn.commit()

f2.write(singleentrytest+"\n")

print FinalDictionary

exit(0)

except Exception as e:

print e

exit(0)

f3.write(singleentrytest+"\n")

conn.close()

f1.close()

f2.close()

f3.close()
