# Supplementary material for: An analysis of deficiencies in the data of interventional drug trials registered with Clinical Trials Registry - India
Source: Trials. 2019 Aug 28;20:535. doi: 10.1186/s13063-019-3592-0 (PMC6712861; doi:10.1186/s13063-019-3592-0)
Supplement: Supplementary file 5 — Expanded methods, used to generate the data in the following sections. Sections 7–16 provide details on (7) The 1331 categories of Type of Study in 12,673 trials, year-wise from 2007–2018; (8) Determining the truly foreign trials; (9) Determining the unambiguously Indian trials, and error rates over time; (10) Determining the unambiguously Multinational trials, and error rates over time; (11) Identifying the actual trials in the categories Foreign, Indian and Multinational: A summary. (12) 55 Interventional Indian cases with Phase listed as PMS, and error rates over time; (13) For the redefined Indian and Multinational sets (i) cases of confusion between PMS and Phase 4 trials; (ii) cases where Type of Trial is BA/BE but Phase is 1–4; and (iii) Sites of study: Incorrect listing of cities. Error rates over time for some of these; (14) Missing data in terms of (i) Name of the PI was missing; (ii) for the redefined Indian set, and the Multinational set, cases where Type of Study was not available, but Phase of Trial was Phase 1, 1/2, 2, 2/3, 3, 3/4 or 4; (iii) Name of Primary Sponsor was missing; and (iv) the state hosting a trial was not listed. Error rates over time for some of these; (15) Examples of types of variations in PIs’ names; and examples, or the entire listing, of problems with ethics committees; and (16) A brief on the 47 trials conducted at the Malpani Multispeciality Hospital, Jaipur, Rajasthan. (ZIP 596 kb) [file 13063_2019_3592_MOESM5_ESM.zip › Additional file 5R1.doc]

**Additional file 5. Expanded methods.** The SQLite queries, and Excel commands where specified, used to generate the data in Additional file 7– Additional file 10 and Additional file 12–Additional file 16.

---------------------------------------------------------------------------------------------------------------

**Additional file 7**

**Sheet name: S7.1_1331 categories of Type of Study**

select BasicDetails.type_of_study , count(*) as 'type' from BasicDetails group by BasicDetails.type_of_study;

**Sheet name: S7.2_Number of categories per year**

select BasicDetails.year_of_reg, count(*) as 'type' from BasicDetails group by BasicDetails.year_of_reg;

**Sheet names: S7.3a_2007, S7.3b_2008, S7.3c_2009, S7.3d_2010, S7.3e_2011, S7.3f_2012, S7.3g_2013, S7.3h_2014, S7.3i_2015, S7.3j_2016, S7.3k_2017, S7.3l_2018**

**2007**

select BasicDetails.year_of_reg, BasicDetails.type_of_study , count(*) as 'type' from BasicDetails WHERE (BasicDetails.year_of_reg == '2007') group by BasicDetails.type_of_study;

**2008**

select BasicDetails.year_of_reg, BasicDetails.type_of_study , count(*) as 'type' from BasicDetails WHERE (BasicDetails.year_of_reg == '2008') group by BasicDetails.type_of_study;

**2009**

select BasicDetails.year_of_reg, BasicDetails.type_of_study , count(*) as 'type' from BasicDetails WHERE (BasicDetails.year_of_reg == '2009') group by BasicDetails.type_of_study;

**2010**

select BasicDetails.year_of_reg, BasicDetails.type_of_study , count(*) as 'type' from BasicDetails WHERE (BasicDetails.year_of_reg == '2010') group by BasicDetails.type_of_study;

**2011**

select BasicDetails.year_of_reg, BasicDetails.type_of_study , count(*) as 'type' from BasicDetails WHERE (BasicDetails.year_of_reg == '2011') group by BasicDetails.type_of_study;

**2012**

select BasicDetails.year_of_reg, BasicDetails.type_of_study , count(*) as 'type' from BasicDetails WHERE (BasicDetails.year_of_reg == '2012') group by BasicDetails.type_of_study;

**2013**

select BasicDetails.year_of_reg, BasicDetails.type_of_study , count(*) as 'type' from BasicDetails WHERE (BasicDetails.year_of_reg == '2013') group by BasicDetails.type_of_study;

**2014**

select BasicDetails.year_of_reg, BasicDetails.type_of_study , count(*) as 'type' from BasicDetails WHERE (BasicDetails.year_of_reg == '2014') group by BasicDetails.type_of_study;

**2015**

select BasicDetails.year_of_reg, BasicDetails.type_of_study , count(*) as 'type' from BasicDetails WHERE (BasicDetails.year_of_reg == '2015') group by BasicDetails.type_of_study;

**2016**

select BasicDetails.year_of_reg, BasicDetails.type_of_study , count(*) as 'type' from BasicDetails WHERE (BasicDetails.year_of_reg == '2016') group by BasicDetails.type_of_study;

**2017**

select BasicDetails.year_of_reg, BasicDetails.type_of_study , count(*) as 'type' from BasicDetails WHERE (BasicDetails.year_of_reg == '2017') group by BasicDetails.type_of_study;

**2018**

select BasicDetails.year_of_reg, BasicDetails.type_of_study , count(*) as 'type' from BasicDetails WHERE (BasicDetails.year_of_reg == '2018') group by BasicDetails.type_of_study;

---------------------------------------------------------------------------------------------------------------

**Additional file 8. Determining the truly foreign trials.**

SQL command used:

select BasicDetails.ctri_number, BasicDetails.countries_of_recruitment, BasicDetails.total_sample_size, BasicDetails.sample_size_from_india, BasicDetails.recruitment_status_of_trial_global, BasicDetails.recruitment_status_of_trial_india, BasicDetails.date_of_first_enrollment_global, BasicDetails.date_of_first_enrollment_india from BasicDetails where ( type_of_study =='Drug' and countries_of_recruitment not like '%India%');

---------------------------------------------------------------------------------------------------------------

**Additional file 9**

**Additional file 9.1. Determining the unambiguously Indian trials.**

***Sheet name: 2070_raw data***

SQL command used:

SELECT BasicDetails.ctri_number,

BasicDetails.countries_of_recruitment,

BasicDetails.recruitment_status_of_trial_global,

BasicDetails.recruitment_status_of_trial_india,

BasicDetails.sample_size_from_india,

BasicDetails.total_sample_size,

BasicDetails.date_of_first_enrollment_global,

BasicDetails.date_of_first_enrollment_india

FROM BasicDetails

WHERE (type_of_study == 'Drug' AND

BasicDetails.countries_of_recruitment == 'India' );

***Sheet name: 2034_accepted from 2070***

SQL command used:

SELECT BasicDetails.ctri_number,

BasicDetails.countries_of_recruitment,

BasicDetails.recruitment_status_of_trial_global,

BasicDetails.recruitment_status_of_trial_india,

BasicDetails.sample_size_from_india,

BasicDetails.total_sample_size,

BasicDetails.date_of_first_enrollment_global,

BasicDetails.date_of_first_enrollment_india

FROM BasicDetails

WHERE (type_of_study == 'Drug' AND

BasicDetails.countries_of_recruitment == 'India' and ( BasicDetails.recruitment_status_of_trial_india != '' and BasicDetails.recruitment_status_of_trial_india != 'Not Applicable') );

***Sheet name: 36_rejected from 2070***

SQL command used:

SELECT BasicDetails.ctri_number,

BasicDetails.countries_of_recruitment,

BasicDetails.recruitment_status_of_trial_global,

BasicDetails.recruitment_status_of_trial_india,

BasicDetails.sample_size_from_india,

BasicDetails.total_sample_size,

BasicDetails.date_of_first_enrollment_global,

BasicDetails.date_of_first_enrollment_india

FROM BasicDetails

WHERE (type_of_study == 'Drug' AND

BasicDetails.countries_of_recruitment == 'India' and ( BasicDetails.recruitment_status_of_trial_india == '' or BasicDetails.recruitment_status_of_trial_india == 'Not Applicable') )

***Sheet name: 1831_accepted from 2034***

SQL command used:

SELECT BasicDetails.ctri_number,

BasicDetails.countries_of_recruitment,

BasicDetails.recruitment_status_of_trial_global,

BasicDetails.recruitment_status_of_trial_india,

BasicDetails.sample_size_from_india,

BasicDetails.total_sample_size,

BasicDetails.date_of_first_enrollment_global,

BasicDetails.date_of_first_enrollment_india

FROM BasicDetails

WHERE (type_of_study == 'Drug' AND

BasicDetails.countries_of_recruitment == 'India' and ( BasicDetails.recruitment_status_of_trial_india != '' and BasicDetails.recruitment_status_of_trial_india != 'Not Applicable') and BasicDetails.recruitment_status_of_trial_global == 'Not Applicable' );

***Sheet name: 203_rejected from 2034***

SQL command used:

SELECT BasicDetails.ctri_number,

BasicDetails.countries_of_recruitment,

BasicDetails.recruitment_status_of_trial_global,

BasicDetails.recruitment_status_of_trial_india,

BasicDetails.sample_size_from_india,

BasicDetails.total_sample_size,

BasicDetails.date_of_first_enrollment_global,

BasicDetails.date_of_first_enrollment_india

FROM BasicDetails

WHERE (type_of_study == 'Drug' AND

BasicDetails.countries_of_recruitment == 'India' and ( BasicDetails.recruitment_status_of_trial_india != '' and BasicDetails.recruitment_status_of_trial_india != 'Not Applicable') and BasicDetails.recruitment_status_of_trial_global != 'Not Applicable' );

***Sheet name: 1823_accepted from 1831***

SQL command used:

SELECT BasicDetails.ctri_number,

BasicDetails.countries_of_recruitment,

BasicDetails.recruitment_status_of_trial_global,

BasicDetails.recruitment_status_of_trial_india,

BasicDetails.sample_size_from_india,

BasicDetails.total_sample_size,

BasicDetails.date_of_first_enrollment_global,

BasicDetails.date_of_first_enrollment_india

FROM BasicDetails

WHERE (type_of_study == 'Drug' AND

BasicDetails.countries_of_recruitment == 'India' and ( BasicDetails.recruitment_status_of_trial_india != '' and BasicDetails.recruitment_status_of_trial_india != 'Not Applicable') and BasicDetails.recruitment_status_of_trial_global == 'Not Applicable' and BasicDetails.sample_size_from_india == BasicDetails.total_sample_size );

***Sheet name: 8_rejected from 1831***

SQL command used:

SELECT BasicDetails.ctri_number,

BasicDetails.countries_of_recruitment,

BasicDetails.recruitment_status_of_trial_global,

BasicDetails.recruitment_status_of_trial_india,

BasicDetails.sample_size_from_india,

BasicDetails.total_sample_size,

BasicDetails.date_of_first_enrollment_global,

BasicDetails.date_of_first_enrollment_india

FROM BasicDetails

WHERE (type_of_study == 'Drug' AND

BasicDetails.countries_of_recruitment == 'India' and ( BasicDetails.recruitment_status_of_trial_india != '' and BasicDetails.recruitment_status_of_trial_india != 'Not Applicable') and BasicDetails.recruitment_status_of_trial_global == 'Not Applicable' and BasicDetails.sample_size_from_india != BasicDetails.total_sample_size );

***Sheet name: 1764_accepted from 1823***

SQL command used

SELECT BasicDetails.ctri_number,

BasicDetails.countries_of_recruitment,

BasicDetails.recruitment_status_of_trial_global,

BasicDetails.recruitment_status_of_trial_india,

BasicDetails.sample_size_from_india,

BasicDetails.total_sample_size,

BasicDetails.date_of_first_enrollment_global,

BasicDetails.date_of_first_enrollment_india

FROM BasicDetails

WHERE (type_of_study == 'Drug' AND

BasicDetails.countries_of_recruitment == 'India' and (BasicDetails.recruitment_status_of_trial_india != '' and BasicDetails.recruitment_status_of_trial_india != 'Not Applicable') and BasicDetails.recruitment_status_of_trial_global == 'Not Applicable' and BasicDetails.sample_size_from_india == BasicDetails.total_sample_size and BasicDetails.date_of_first_enrollment_global == 'Date Missing' );

***Sheet name: 59_rejected from 1823***

SQL command used:

SELECT BasicDetails.ctri_number,

BasicDetails.countries_of_recruitment,

BasicDetails.recruitment_status_of_trial_global,

BasicDetails.recruitment_status_of_trial_india,

BasicDetails.sample_size_from_india,

BasicDetails.total_sample_size,

BasicDetails.date_of_first_enrollment_global,

BasicDetails.date_of_first_enrollment_india

FROM BasicDetails

WHERE (type_of_study == 'Drug' AND

BasicDetails.countries_of_recruitment == 'India' and ( BasicDetails.recruitment_status_of_trial_india != '' and BasicDetails.recruitment_status_of_trial_india != 'Not Applicable') and BasicDetails.recruitment_status_of_trial_global == 'Not Applicable' and BasicDetails.sample_size_from_india == BasicDetails.total_sample_size and BasicDetails.date_of_first_enrollment_global != 'Date Missing' );

***Sheet name: 36_both dates matching***

SQL command used:

SELECT BasicDetails.ctri_number,

BasicDetails.countries_of_recruitment,

BasicDetails.recruitment_status_of_trial_global,

BasicDetails.recruitment_status_of_trial_india,

BasicDetails.sample_size_from_india,

BasicDetails.total_sample_size,

BasicDetails.date_of_first_enrollment_global,

BasicDetails.date_of_first_enrollment_india

FROM BasicDetails

WHERE (type_of_study == 'Drug' AND

BasicDetails.countries_of_recruitment == 'India' and ( BasicDetails.recruitment_status_of_trial_india != '' and BasicDetails.recruitment_status_of_trial_india != 'Not Applicable') and BasicDetails.recruitment_status_of_trial_global == 'Not Applicable' and BasicDetails.sample_size_from_india == BasicDetails.total_sample_size and BasicDetails.date_of_first_enrollment_global != 'Date Missing' and BasicDetails.date_of_first_enrollment_global == BasicDetails.date_of_first_enrollment_india);

***Sheet name: Breakup of 59_13_different dates in col E and col F***

SQL command used:

SELECT BasicDetails.ctri_number,

BasicDetails.countries_of_recruitment,

BasicDetails.recruitment_status_of_trial_global,

BasicDetails.recruitment_status_of_trial_india,

BasicDetails.sample_size_from_india,

BasicDetails.total_sample_size,

BasicDetails.date_of_first_enrollment_global,

BasicDetails.date_of_first_enrollment_india

FROM BasicDetails

WHERE (type_of_study == 'Drug' AND

BasicDetails.countries_of_recruitment == 'India' and ( BasicDetails.recruitment_status_of_trial_india != '' and BasicDetails.recruitment_status_of_trial_india != 'Not Applicable') and BasicDetails.recruitment_status_of_trial_global == 'Not Applicable' and BasicDetails.sample_size_from_india == BasicDetails.total_sample_size and BasicDetails.date_of_first_enrollment_global != 'Date Missing' and BasicDetails.date_of_first_enrollment_global != BasicDetails.date_of_first_enrollment_india );

***Sheet name: Breakup of 59_10_col F date missing***

SQL command used:

SELECT BasicDetails.ctri_number,

BasicDetails.countries_of_recruitment,

BasicDetails.recruitment_status_of_trial_global,

BasicDetails.recruitment_status_of_trial_india,

BasicDetails.sample_size_from_india,

BasicDetails.total_sample_size,

BasicDetails.date_of_first_enrollment_global,

BasicDetails.date_of_first_enrollment_india

FROM BasicDetails

WHERE (type_of_study == 'Drug' AND

BasicDetails.countries_of_recruitment == 'India' and ( BasicDetails.recruitment_status_of_trial_india != '' and BasicDetails.recruitment_status_of_trial_india != 'Not Applicable') and BasicDetails.recruitment_status_of_trial_global == 'Not Applicable' and BasicDetails.sample_size_from_india == BasicDetails.total_sample_size and BasicDetails.date_of_first_enrollment_global != 'Date Missing' and BasicDetails.date_of_first_enrollment_global != BasicDetails.date_of_first_enrollment_india );

**Sheet name: S9.2_The errors in the S9.1 File, over time**

**Command used to reject 36 cases**

SELECT

BasicDetails.year_of_reg , count(*) as 'Year'

FROM BasicDetails

WHERE (type_of_study == 'Drug' AND

BasicDetails.countries_of_recruitment == 'India' and ( BasicDetails.recruitment_status_of_trial_india == '' or BasicDetails.recruitment_status_of_trial_india == 'Not Applicable') ) group by BasicDetails.year_of_reg;

**Command used to reject 203 cases**

SELECT BasicDetails.year_of_reg , count(*) as 'Year'

FROM BasicDetails

WHERE (type_of_study == 'Drug' AND

BasicDetails.countries_of_recruitment == 'India' and ( BasicDetails.recruitment_status_of_trial_india != '' and BasicDetails.recruitment_status_of_trial_india != 'Not Applicable') and BasicDetails.recruitment_status_of_trial_global != 'Not Applicable' ) group by BasicDetails.year_of_reg;

**Command used to reject 8 cases**

SELECT BasicDetails.year_of_reg , count(*) as 'Year'

FROM BasicDetails

WHERE (type_of_study == 'Drug' AND

BasicDetails.countries_of_recruitment == 'India' and ( BasicDetails.recruitment_status_of_trial_india != '' and BasicDetails.recruitment_status_of_trial_india != 'Not Applicable') and BasicDetails.recruitment_status_of_trial_global == 'Not Applicable' and BasicDetails.sample_size_from_india != BasicDetails.total_sample_size )group by BasicDetails.year_of_reg;

**Command used to reject 59 cases**

SELECT BasicDetails.year_of_reg , count(*) as 'Year'

FROM BasicDetails

WHERE (type_of_study == 'Drug' AND

BasicDetails.countries_of_recruitment == 'India' and ( BasicDetails.recruitment_status_of_trial_india != '' and BasicDetails.recruitment_status_of_trial_india != 'Not Applicable') and BasicDetails.recruitment_status_of_trial_global == 'Not Applicable' and BasicDetails.sample_size_from_india == BasicDetails.total_sample_size and BasicDetails.date_of_first_enrollment_global != 'Date Missing' )group by BasicDetails.year_of_reg;

Later, we manually grouped all the records with errors. Then, we used the pivot table option in Excel to generate the data in column D, '**Number of errors**', of Additional file 9.2.

---------------------------------------------------------------------------------------------------------------

**Additional file 10**

**Additional file 10.1. Determining the unambiguously Multinational trials.**

***Sheet name: 640_raw_data_india_etc***

SQL command used:

SELECT BasicDetails.ctri_number,

BasicDetails.countries_of_recruitment,

BasicDetails.recruitment_status_of_trial_global,

BasicDetails.recruitment_status_of_trial_india,

BasicDetails.sample_size_from_india,

BasicDetails.total_sample_size,

BasicDetails.date_of_first_enrollment_global,

BasicDetails.date_of_first_enrollment_india

FROM BasicDetails

WHERE (type_of_study == 'Drug' AND

(BasicDetails.countries_of_recruitment like '%,India,%' or BasicDetails.countries_of_recruitment like 'India,%' or BasicDetails.countries_of_recruitment like '%,India' and BasicDetails.countries_of_recruitment !='India'));

***Sheet name: 631_accepted from 640***

SQL command used:

SELECT BasicDetails.ctri_number,

BasicDetails.countries_of_recruitment,

BasicDetails.recruitment_status_of_trial_global,

BasicDetails.recruitment_status_of_trial_india,

BasicDetails.sample_size_from_india,

BasicDetails.total_sample_size,

BasicDetails.date_of_first_enrollment_global,

BasicDetails.date_of_first_enrollment_india

FROM BasicDetails

WHERE (type_of_study == 'Drug' AND

(BasicDetails.countries_of_recruitment like '%,India,%' or BasicDetails.countries_of_recruitment like 'India,%' or BasicDetails.countries_of_recruitment like '%,India' and BasicDetails.countries_of_recruitment !='India') and BasicDetails.recruitment_status_of_trial_india != 'Not Applicable' and BasicDetails.recruitment_status_of_trial_global != 'Not Applicable' );

***Sheet name: 9_rejected from 640***

SQL command used:

SELECT BasicDetails.ctri_number,

BasicDetails.countries_of_recruitment,

BasicDetails.recruitment_status_of_trial_global,

BasicDetails.recruitment_status_of_trial_india,

BasicDetails.sample_size_from_india,

BasicDetails.total_sample_size,

BasicDetails.date_of_first_enrollment_global,

BasicDetails.date_of_first_enrollment_india

FROM BasicDetails

WHERE (type_of_study == 'Drug' AND

(BasicDetails.countries_of_recruitment like '%,India,%' or BasicDetails.countries_of_recruitment like 'India,%' or BasicDetails.countries_of_recruitment like '%,India' and BasicDetails.countries_of_recruitment !='India') and BasicDetails.recruitment_status_of_trial_india != 'Not Applicable' and BasicDetails.recruitment_status_of_trial_global == 'Not Applicable' );

***Sheet name: 628_accepted from 631***

SQL command used:

SELECT BasicDetails.ctri_number,

BasicDetails.countries_of_recruitment,

BasicDetails.recruitment_status_of_trial_global,

BasicDetails.recruitment_status_of_trial_india,

BasicDetails.sample_size_from_india,

BasicDetails.total_sample_size,

BasicDetails.date_of_first_enrollment_global,

BasicDetails.date_of_first_enrollment_india

FROM BasicDetails

WHERE (type_of_study == 'Drug' AND

(BasicDetails.countries_of_recruitment like '%,India,%' or BasicDetails.countries_of_recruitment like 'India,%' or BasicDetails.countries_of_recruitment like '%,India' and BasicDetails.countries_of_recruitment !='India') and BasicDetails.recruitment_status_of_trial_india != 'Not Applicable' and BasicDetails.recruitment_status_of_trial_global != 'Not Applicable' and BasicDetails.sample_size_from_india != BasicDetails.total_sample_size );

***Sheet name: 3_rejected from 631***

SQL command used:

SELECT BasicDetails.ctri_number,

BasicDetails.countries_of_recruitment,

BasicDetails.recruitment_status_of_trial_global,

BasicDetails.recruitment_status_of_trial_india,

BasicDetails.sample_size_from_india,

BasicDetails.total_sample_size,

BasicDetails.date_of_first_enrollment_global,

BasicDetails.date_of_first_enrollment_india

FROM BasicDetails

WHERE (type_of_study == 'Drug' AND

(BasicDetails.countries_of_recruitment like '%,India,%' or BasicDetails.countries_of_recruitment like 'India,%' or BasicDetails.countries_of_recruitment like '%,India' and BasicDetails.countries_of_recruitment !='India') and BasicDetails.recruitment_status_of_trial_india != 'Not Applicable' and BasicDetails.recruitment_status_of_trial_global == 'Not Applicable' and BasicDetails.sample_size_from_india == BasicDetails.total_sample_size );

***Sheet name: 621_accepted from 628***

SQL command used:

SELECT BasicDetails.ctri_number,

BasicDetails.countries_of_recruitment,

BasicDetails.recruitment_status_of_trial_global,

BasicDetails.recruitment_status_of_trial_india,

BasicDetails.sample_size_from_india,

BasicDetails.total_sample_size,

BasicDetails.date_of_first_enrollment_global,

BasicDetails.date_of_first_enrollment_india

FROM BasicDetails

WHERE (type_of_study == 'Drug' AND

(BasicDetails.countries_of_recruitment like '%,India,%' or BasicDetails.countries_of_recruitment like 'India,%' or BasicDetails.countries_of_recruitment like '%,India' and BasicDetails.countries_of_recruitment !='India') and BasicDetails.recruitment_status_of_trial_india != 'Not Applicable' and BasicDetails.recruitment_status_of_trial_global != 'Not Applicable' and BasicDetails.sample_size_from_india != BasicDetails.total_sample_size and BasicDetails.sample_size_from_india != '"0"' and BasicDetails.sample_size_from_india != '""' );

***Sheet name: 7_rejected from 628***

2 SQL commands used:

SELECT BasicDetails.ctri_number,

BasicDetails.countries_of_recruitment,

BasicDetails.recruitment_status_of_trial_global,

BasicDetails.recruitment_status_of_trial_india,

BasicDetails.sample_size_from_india,

BasicDetails.total_sample_size,

BasicDetails.date_of_first_enrollment_global,

BasicDetails.date_of_first_enrollment_india

FROM BasicDetails

WHERE (type_of_study == 'Drug' AND

(BasicDetails.countries_of_recruitment like '%,India,%' or BasicDetails.countries_of_recruitment like 'India,%' or BasicDetails.countries_of_recruitment like '%,India' and BasicDetails.countries_of_recruitment !='India') and BasicDetails.recruitment_status_of_trial_india != 'Not Applicable' and BasicDetails.recruitment_status_of_trial_global != 'Not Applicable' and BasicDetails.sample_size_from_india != BasicDetails.total_sample_size and BasicDetails.sample_size_from_india == '"0"');

Output count: 3

SELECT BasicDetails.ctri_number,

BasicDetails.countries_of_recruitment,

BasicDetails.recruitment_status_of_trial_global,

BasicDetails.recruitment_status_of_trial_india,

BasicDetails.sample_size_from_india,

BasicDetails.total_sample_size,

BasicDetails.date_of_first_enrollment_global,

BasicDetails.date_of_first_enrollment_india

FROM BasicDetails

WHERE (type_of_study == 'Drug' AND

(BasicDetails.countries_of_recruitment like '%,India,%' or BasicDetails.countries_of_recruitment like 'India,%' or BasicDetails.countries_of_recruitment like '%,India' and BasicDetails.countries_of_recruitment !='India') and BasicDetails.recruitment_status_of_trial_india != 'Not Applicable' and BasicDetails.recruitment_status_of_trial_global != 'Not Applicable' and BasicDetails.sample_size_from_india != BasicDetails.total_sample_size and BasicDetails.sample_size_from_india == '""' );

Output count: 4

Total = 7 (3+4)

***Sheet name: 609_accepted from 621***

SQL command used:

SELECT BasicDetails.ctri_number,

BasicDetails.countries_of_recruitment,

BasicDetails.recruitment_status_of_trial_global,

BasicDetails.recruitment_status_of_trial_india,

BasicDetails.sample_size_from_india,

BasicDetails.total_sample_size,

BasicDetails.date_of_first_enrollment_global,

BasicDetails.date_of_first_enrollment_india

FROM BasicDetails

WHERE (type_of_study == 'Drug' AND

(BasicDetails.countries_of_recruitment like '%,India,%' or BasicDetails.countries_of_recruitment like 'India,%' or BasicDetails.countries_of_recruitment like '%,India' and BasicDetails.countries_of_recruitment !='India') and BasicDetails.recruitment_status_of_trial_india != 'Not Applicable' and BasicDetails.recruitment_status_of_trial_global != 'Not Applicable' and BasicDetails.sample_size_from_india != BasicDetails.total_sample_size and BasicDetails.sample_size_from_india != '"0"' and BasicDetails.sample_size_from_india != '""' and BasicDetails.date_of_first_enrollment_india != 'Date Missing' );

***Sheet name: 12_rejected from 621***

SQL command used:

SELECT BasicDetails.ctri_number,

BasicDetails.countries_of_recruitment,

BasicDetails.recruitment_status_of_trial_global,

BasicDetails.recruitment_status_of_trial_india,

BasicDetails.sample_size_from_india,

BasicDetails.total_sample_size,

BasicDetails.date_of_first_enrollment_global,

BasicDetails.date_of_first_enrollment_india

FROM BasicDetails

WHERE (type_of_study == 'Drug' AND

(BasicDetails.countries_of_recruitment like '%,India,%' or BasicDetails.countries_of_recruitment like 'India,%' or BasicDetails.countries_of_recruitment like '%,India' and BasicDetails.countries_of_recruitment !='India') and BasicDetails.recruitment_status_of_trial_india != 'Not Applicable' and BasicDetails.recruitment_status_of_trial_global != 'Not Applicable' and BasicDetails.sample_size_from_india != BasicDetails.total_sample_size and BasicDetails.sample_size_from_india != '"0"' and BasicDetails.sample_size_from_india != '""' and BasicDetails.date_of_first_enrollment_india == 'Date Missing' );

**Sheet name: S10.2_The errors in the S10.1 File, over time**

**Command used to reject 9 cases**

SELECT BasicDetails.year_of_reg , count(*) as 'Year'

FROM BasicDetails

WHERE (type_of_study == 'Drug' AND

(BasicDetails.countries_of_recruitment like '%,India,%' or BasicDetails.countries_of_recruitment like 'India,%' or BasicDetails.countries_of_recruitment like '%,India' and BasicDetails.countries_of_recruitment !='India') and BasicDetails.recruitment_status_of_trial_india != 'Not Applicable' and BasicDetails.recruitment_status_of_trial_global == 'Not Applicable' )group by BasicDetails.year_of_reg;

**Command used to reject 3 cases**

SELECT BasicDetails.year_of_reg , count(*) as 'Year'

FROM BasicDetails

WHERE (type_of_study == 'Drug' AND

(BasicDetails.countries_of_recruitment like '%,India,%' or BasicDetails.countries_of_recruitment like 'India,%' or BasicDetails.countries_of_recruitment like '%,India' and BasicDetails.countries_of_recruitment !='India') and BasicDetails.recruitment_status_of_trial_india != 'Not Applicable' and BasicDetails.recruitment_status_of_trial_global == 'Not Applicable' and BasicDetails.sample_size_from_india == BasicDetails.total_sample_size )group by BasicDetails.year_of_reg;

**Command used to reject 3 cases**

SELECT BasicDetails.year_of_reg , count(*) as 'Year'

FROM BasicDetails

WHERE (type_of_study == 'Drug' AND

(BasicDetails.countries_of_recruitment like '%,India,%' or BasicDetails.countries_of_recruitment like 'India,%' or BasicDetails.countries_of_recruitment like '%,India' and BasicDetails.countries_of_recruitment !='India') and BasicDetails.recruitment_status_of_trial_india != 'Not Applicable' and BasicDetails.recruitment_status_of_trial_global != 'Not Applicable' and BasicDetails.sample_size_from_india != BasicDetails.total_sample_size and BasicDetails.sample_size_from_india == '"0"')group by BasicDetails.year_of_reg;

**Command used to reject 4 cases**

SELECT BasicDetails.year_of_reg , count(*) as 'Year'

FROM BasicDetails

WHERE (type_of_study == 'Drug' AND

(BasicDetails.countries_of_recruitment like '%,India,%' or BasicDetails.countries_of_recruitment like 'India,%' or BasicDetails.countries_of_recruitment like '%,India' and BasicDetails.countries_of_recruitment !='India') and BasicDetails.recruitment_status_of_trial_india != 'Not Applicable' and BasicDetails.recruitment_status_of_trial_global != 'Not Applicable' and BasicDetails.sample_size_from_india != BasicDetails.total_sample_size and BasicDetails.sample_size_from_india == '""' )group by BasicDetails.year_of_reg;

**Command used to reject 12 cases**

SELECT BasicDetails.year_of_reg , count(*) as 'Year'

FROM BasicDetails

WHERE (type_of_study == 'Drug' AND

(BasicDetails.countries_of_recruitment like '%,India,%' or BasicDetails.countries_of_recruitment like 'India,%' or BasicDetails.countries_of_recruitment like '%,India' and BasicDetails.countries_of_recruitment !='India') and BasicDetails.recruitment_status_of_trial_india != 'Not Applicable' and BasicDetails.recruitment_status_of_trial_global != 'Not Applicable' and BasicDetails.sample_size_from_india != BasicDetails.total_sample_size and BasicDetails.sample_size_from_india != '"0"' and BasicDetails.sample_size_from_india != '""' and BasicDetails.date_of_first_enrollment_india == 'Date Missing' )group by BasicDetails.year_of_reg;

Later, we manually grouped all the records with errors. Then, we used the pivot table option in Excel to generate the data in column D, '**Number of errors**', of Additional file 10.2.

---------------------------------------------------------------------------------------------------------------

**Additional file 12**

**Sheet name: S12a_55 PMS cases**

SELECT BasicDetails.ctri_number, BasicDetails.year_of_reg

FROM BasicDetails

WHERE (type_of_study == 'Drug' AND

BasicDetails.countries_of_recruitment == 'India' and BasicDetails.recruitment_status_of_trial_india != 'Not Applicable' and BasicDetails.recruitment_status_of_trial_global == 'Not Applicable' and BasicDetails.sample_size_from_india == BasicDetails.total_sample_size and BasicDetails.date_of_first_enrollment_global == 'Date Missing' and BasicDetails.type_of_trial == 'Interventional'and BasicDetails.phase_of_trial == 'Post Marketing Surveillance' );

**Sheet name: S12b_55 PMS cases_Errors over time**

We interrogated 1655 cases. These were generated using the commands

**Grouping of 1655 cases in years**

SELECT BasicDetails.year_of_reg, count(*) as 'type'

FROM BasicDetails

WHERE (type_of_study == 'Drug' AND

BasicDetails.countries_of_recruitment == 'India' and (BasicDetails.recruitment_status_of_trial_india != '' and BasicDetails.recruitment_status_of_trial_india != 'Not Applicable') and BasicDetails.recruitment_status_of_trial_global == 'Not Applicable' and BasicDetails.sample_size_from_india == BasicDetails.total_sample_size and BasicDetails.date_of_first_enrollment_global == 'Date Missing' and BasicDetails.type_of_trial == 'Interventional' ) group by BasicDetails.year_of_reg;

**Grouping of 55 cases in years**

SELECT BasicDetails.year_of_reg , count(*) as 'year'

FROM BasicDetails

WHERE (type_of_study == 'Drug' AND

BasicDetails.countries_of_recruitment == 'India' and BasicDetails.recruitment_status_of_trial_india != 'Not Applicable' and BasicDetails.recruitment_status_of_trial_global == 'Not Applicable' and BasicDetails.sample_size_from_india == BasicDetails.total_sample_size and BasicDetails.date_of_first_enrollment_global == 'Date Missing' and BasicDetails.type_of_trial == 'Interventional'and BasicDetails.phase_of_trial == 'Post Marketing Surveillance' ) group by BasicDetails.year_of_reg ;

**---------------------------------------------------------------------------------------------------------------**

**Additional file 13**

**Sheet name: 13.1a and 13.1b_Indian_PMS and Phase 4**

**Grouping of 109 cases in years**

SELECT BasicDetails.year_of_reg , count(*) as 'Year'

FROM BasicDetails

WHERE (type_of_study == 'Drug' AND

BasicDetails.countries_of_recruitment == 'India' and (BasicDetails.recruitment_status_of_trial_india != '' and BasicDetails.recruitment_status_of_trial_india != 'Not Applicable') and BasicDetails.recruitment_status_of_trial_global == 'Not Applicable' and BasicDetails.sample_size_from_india == BasicDetails.total_sample_size and BasicDetails.date_of_first_enrollment_global == 'Date Missing' and BasicDetails.type_of_trial =='PMS' )group by BasicDetails.year_of_reg;

**Command used to extract 34 cases**

SELECT BasicDetails.ctri_number

,BasicDetails.year_of_reg, BasicDetails.type_of_trial, BasicDetails.phase_of_trial

FROM BasicDetails

WHERE (type_of_study == 'Drug' AND

BasicDetails.countries_of_recruitment == 'India' and (BasicDetails.recruitment_status_of_trial_india != '' and BasicDetails.recruitment_status_of_trial_india != 'Not Applicable') and BasicDetails.recruitment_status_of_trial_global == 'Not Applicable' and BasicDetails.sample_size_from_india == BasicDetails.total_sample_size and BasicDetails.date_of_first_enrollment_global == 'Date Missing' and BasicDetails.type_of_trial =='PMS' and BasicDetails.phase_of_trial == 'Phase 4' );

**Grouping the 34 cases in years**

SELECT BasicDetails.year_of_reg , count(*) as 'Year'

FROM BasicDetails

WHERE (type_of_study == 'Drug' AND

BasicDetails.countries_of_recruitment == 'India' and (BasicDetails.recruitment_status_of_trial_india != '' and BasicDetails.recruitment_status_of_trial_india != 'Not Applicable') and BasicDetails.recruitment_status_of_trial_global == 'Not Applicable' and BasicDetails.sample_size_from_india == BasicDetails.total_sample_size and BasicDetails.date_of_first_enrollment_global == 'Date Missing' and BasicDetails.type_of_trial =='PMS' and BasicDetails.phase_of_trial == 'Phase 4' ) group by BasicDetails.year_of_reg;

**Sheet name: 13.1c_Multinational_PMS and Phase 4**

SELECT BasicDetails.ctri_number

,BasicDetails.year_of_reg, BasicDetails.type_of_trial, BasicDetails.phase_of_trial

FROM BasicDetails

WHERE (type_of_study == 'Drug' AND

(BasicDetails.countries_of_recruitment like '%,India,%' or BasicDetails.countries_of_recruitment like 'India,%' or BasicDetails.countries_of_recruitment like '%,India' and BasicDetails.countries_of_recruitment !='India') and BasicDetails.recruitment_status_of_trial_india != 'Not Applicable' and BasicDetails.recruitment_status_of_trial_global != 'Not Applicable' and BasicDetails.sample_size_from_india != BasicDetails.total_sample_size and BasicDetails.sample_size_from_india != '"0"' and BasicDetails.sample_size_from_india != '""' and BasicDetails.date_of_first_enrollment_india != 'Date Missing' and BasicDetails.type_of_trial =='PMS' and BasicDetails.phase_of_trial == 'Phase 4');

**Sheet name: 13.2a and 13.2b_Indian_BA_BE and Phases**

**Grouping of 243 cases in years**

SELECT BasicDetails.year_of_reg , count(*) as 'Year'

FROM BasicDetails

WHERE (

BasicDetails.countries_of_recruitment == 'India' and (BasicDetails.recruitment_status_of_trial_india != '' and BasicDetails.recruitment_status_of_trial_india != 'Not Applicable') and BasicDetails.recruitment_status_of_trial_global == 'Not Applicable' and BasicDetails.sample_size_from_india == BasicDetails.total_sample_size and BasicDetails.date_of_first_enrollment_global == 'Date Missing' and BasicDetails.type_of_trial =='BA/BE' )group by BasicDetails.year_of_reg;

**Command used to extract Phase 1 cases**

SELECT BasicDetails.ctri_number

,BasicDetails.year_of_reg, BasicDetails.type_of_trial, BasicDetails.phase_of_trial

FROM BasicDetails

WHERE (

BasicDetails.countries_of_recruitment == 'India' and (BasicDetails.recruitment_status_of_trial_india != '' and BasicDetails.recruitment_status_of_trial_india != 'Not Applicable') and BasicDetails.recruitment_status_of_trial_global == 'Not Applicable' and BasicDetails.sample_size_from_india == BasicDetails.total_sample_size and BasicDetails.date_of_first_enrollment_global == 'Date Missing' and BasicDetails.type_of_trial =='BA/BE' and BasicDetails.phase_of_trial == 'Phase 1');

**Command used to extract Phase 2 cases**

SELECT BasicDetails.ctri_number

,BasicDetails.year_of_reg, BasicDetails.type_of_trial, BasicDetails.phase_of_trial

FROM BasicDetails

WHERE (

BasicDetails.countries_of_recruitment == 'India' and (BasicDetails.recruitment_status_of_trial_india != '' and BasicDetails.recruitment_status_of_trial_india != 'Not Applicable') and BasicDetails.recruitment_status_of_trial_global == 'Not Applicable' and BasicDetails.sample_size_from_india == BasicDetails.total_sample_size and BasicDetails.date_of_first_enrollment_global == 'Date Missing' and BasicDetails.type_of_trial =='BA/BE' and BasicDetails.phase_of_trial == 'Phase 2');

**Command used to extract Phase 3 cases**

SELECT BasicDetails.ctri_number

,BasicDetails.year_of_reg, BasicDetails.type_of_trial, BasicDetails.phase_of_trial

FROM BasicDetails

WHERE (

BasicDetails.countries_of_recruitment == 'India' and (BasicDetails.recruitment_status_of_trial_india != '' and BasicDetails.recruitment_status_of_trial_india != 'Not Applicable') and BasicDetails.recruitment_status_of_trial_global == 'Not Applicable' and BasicDetails.sample_size_from_india == BasicDetails.total_sample_size and BasicDetails.date_of_first_enrollment_global == 'Date Missing' and BasicDetails.type_of_trial =='BA/BE' and BasicDetails.phase_of_trial == 'Phase 3');

**Command used to extract Phase 4 cases**

SELECT BasicDetails.ctri_number

,BasicDetails.year_of_reg, BasicDetails.type_of_trial, BasicDetails.phase_of_trial

FROM BasicDetails

WHERE (

BasicDetails.countries_of_recruitment == 'India' and (BasicDetails.recruitment_status_of_trial_india != '' and BasicDetails.recruitment_status_of_trial_india != 'Not Applicable') and BasicDetails.recruitment_status_of_trial_global == 'Not Applicable' and BasicDetails.sample_size_from_india == BasicDetails.total_sample_size and BasicDetails.date_of_first_enrollment_global == 'Date Missing' and BasicDetails.type_of_trial =='BA/BE' and BasicDetails.phase_of_trial == 'Phase 4');

**Command used to extract Phase 1/ Phase 2 cases**

SELECT BasicDetails.ctri_number

,BasicDetails.year_of_reg, BasicDetails.type_of_trial, BasicDetails.phase_of_trial

FROM BasicDetails

WHERE (

BasicDetails.countries_of_recruitment == 'India' and (BasicDetails.recruitment_status_of_trial_india != '' and BasicDetails.recruitment_status_of_trial_india != 'Not Applicable') and BasicDetails.recruitment_status_of_trial_global == 'Not Applicable' and BasicDetails.sample_size_from_india == BasicDetails.total_sample_size and BasicDetails.date_of_first_enrollment_global == 'Date Missing' and BasicDetails.type_of_trial =='BA/BE' and BasicDetails.phase_of_trial == 'Phase 1/ Phase 2');

**Command used to extract Phase 3/ Phase 4 cases**

SELECT BasicDetails.ctri_number

,BasicDetails.year_of_reg, BasicDetails.type_of_trial, BasicDetails.phase_of_trial

FROM BasicDetails

WHERE (

BasicDetails.countries_of_recruitment == 'India' and (BasicDetails.recruitment_status_of_trial_india != '' and BasicDetails.recruitment_status_of_trial_india != 'Not Applicable') and BasicDetails.recruitment_status_of_trial_global == 'Not Applicable' and BasicDetails.sample_size_from_india == BasicDetails.total_sample_size and BasicDetails.date_of_first_enrollment_global == 'Date Missing' and BasicDetails.type_of_trial =='BA/BE' and BasicDetails.phase_of_trial == 'Phase 3/ Phase 4');

Later, we manually grouped all the records with errors. Then, we used the pivot table option in Excel to generate the data in column I, '**Number of errors**'.

**Sheet name: 13.2c_Multinational_BA_BE and Phases**

**Command used to extract Phase 1 cases**

SELECT BasicDetails.ctri_number

,BasicDetails.year_of_reg, BasicDetails.type_of_trial, BasicDetails.phase_of_trial

FROM BasicDetails

WHERE (

(BasicDetails.countries_of_recruitment like '%,India,%' or BasicDetails.countries_of_recruitment like 'India,%' or BasicDetails.countries_of_recruitment like '%,India' and BasicDetails.countries_of_recruitment !='India') and BasicDetails.recruitment_status_of_trial_india != 'Not Applicable' and BasicDetails.recruitment_status_of_trial_global != 'Not Applicable' and BasicDetails.sample_size_from_india != BasicDetails.total_sample_size and BasicDetails.sample_size_from_india != '"0"' and BasicDetails.sample_size_from_india != '""' and BasicDetails.date_of_first_enrollment_india != 'Date Missing' and BasicDetails.type_of_trial =='BA/BE' and BasicDetails.phase_of_trial == 'Phase 1');

**Command used to extract Phase 3 cases**

SELECT BasicDetails.ctri_number

,BasicDetails.year_of_reg, BasicDetails.type_of_trial, BasicDetails.phase_of_trial

FROM BasicDetails

WHERE (

(BasicDetails.countries_of_recruitment like '%,India,%' or BasicDetails.countries_of_recruitment like 'India,%' or BasicDetails.countries_of_recruitment like '%,India' and BasicDetails.countries_of_recruitment !='India') and BasicDetails.recruitment_status_of_trial_india != 'Not Applicable' and BasicDetails.recruitment_status_of_trial_global != 'Not Applicable' and BasicDetails.sample_size_from_india != BasicDetails.total_sample_size and BasicDetails.sample_size_from_india != '"0"' and BasicDetails.sample_size_from_india != '""' and BasicDetails.date_of_first_enrollment_india != 'Date Missing' and BasicDetails.type_of_trial =='BA/BE' and BasicDetails.phase_of_trial == 'Phase 3');

**Sheet name: 13.3a and 13.3b_Indian_Incorrect listing of cities**

**Grouping of 6242 cases in years**

SELECT BasicDetails.year_of_reg, count(*) as 'type' FROM BasicDetails, SitesOfStudy

WHERE ((SitesOfStudy.ctri_number == BasicDetails.ctri_number) and type_of_study == 'Drug' AND

BasicDetails.countries_of_recruitment == 'India' and (BasicDetails.recruitment_status_of_trial_india != '' and BasicDetails.recruitment_status_of_trial_india != 'Not Applicable') and BasicDetails.recruitment_status_of_trial_global == 'Not Applicable' and BasicDetails.sample_size_from_india == BasicDetails.total_sample_size and BasicDetails.date_of_first_enrollment_global == 'Date Missing' and BasicDetails.type_of_trial == 'Interventional' ) group by BasicDetails.year_of_reg;

**Command to extract cases with city name: Mumbai (Suburban)**

SELECT BasicDetails.ctri_number ,BasicDetails.year_of_reg, SitesOfStudy.city FROM BasicDetails, SitesOfStudy

WHERE ((SitesOfStudy.ctri_number == BasicDetails.ctri_number) and type_of_study == 'Drug' AND

BasicDetails.countries_of_recruitment == 'India' and (BasicDetails.recruitment_status_of_trial_india != '' and BasicDetails.recruitment_status_of_trial_india != 'Not Applicable') and BasicDetails.recruitment_status_of_trial_global == 'Not Applicable' and BasicDetails.sample_size_from_india == BasicDetails.total_sample_size and BasicDetails.date_of_first_enrollment_global == 'Date Missing' and BasicDetails.type_of_trial == 'Interventional' and SitesOfStudy.city == 'Mumbai (Suburban)');

**Command to extract cases with city name: Central**

SELECT BasicDetails.ctri_number ,BasicDetails.year_of_reg, SitesOfStudy.city FROM BasicDetails, SitesOfStudy

WHERE ((SitesOfStudy.ctri_number == BasicDetails.ctri_number) and type_of_study == 'Drug' AND

BasicDetails.countries_of_recruitment == 'India' and (BasicDetails.recruitment_status_of_trial_india != '' and BasicDetails.recruitment_status_of_trial_india != 'Not Applicable') and BasicDetails.recruitment_status_of_trial_global == 'Not Applicable' and BasicDetails.sample_size_from_india == BasicDetails.total_sample_size and BasicDetails.date_of_first_enrollment_global == 'Date Missing' and BasicDetails.type_of_trial == 'Interventional' and SitesOfStudy.city == 'Central');

**Command to extract cases with city name: South**

SELECT BasicDetails.ctri_number ,BasicDetails.year_of_reg, SitesOfStudy.city FROM BasicDetails, SitesOfStudy

WHERE ((SitesOfStudy.ctri_number == BasicDetails.ctri_number) and type_of_study == 'Drug' AND

BasicDetails.countries_of_recruitment == 'India' and (BasicDetails.recruitment_status_of_trial_india != '' and BasicDetails.recruitment_status_of_trial_india != 'Not Applicable') and BasicDetails.recruitment_status_of_trial_global == 'Not Applicable' and BasicDetails.sample_size_from_india == BasicDetails.total_sample_size and BasicDetails.date_of_first_enrollment_global == 'Date Missing' and BasicDetails.type_of_trial == 'Interventional' and SitesOfStudy.city == 'South');

**Command to extract cases with city name: East**

SELECT BasicDetails.ctri_number ,BasicDetails.year_of_reg, SitesOfStudy.city, SitesOfStudy.state FROM BasicDetails, SitesOfStudy

WHERE ((SitesOfStudy.ctri_number == BasicDetails.ctri_number) and type_of_study == 'Drug' AND

BasicDetails.countries_of_recruitment == 'India' and (BasicDetails.recruitment_status_of_trial_india != '' and BasicDetails.recruitment_status_of_trial_india != 'Not Applicable') and BasicDetails.recruitment_status_of_trial_global == 'Not Applicable' and BasicDetails.sample_size_from_india == BasicDetails.total_sample_size and BasicDetails.date_of_first_enrollment_global == 'Date Missing' and BasicDetails.type_of_trial == 'Interventional' and SitesOfStudy.city == 'East');

**[One case pulled out by the above command mentioned Sikkim as the state. It was manually removed and not included in any further analysis]**

**Command to extract cases with city name: South West**

SELECT BasicDetails.ctri_number ,BasicDetails.year_of_reg, SitesOfStudy.city FROM BasicDetails, SitesOfStudy

WHERE ((SitesOfStudy.ctri_number == BasicDetails.ctri_number) and type_of_study == 'Drug' AND

BasicDetails.countries_of_recruitment == 'India' and (BasicDetails.recruitment_status_of_trial_india != '' and BasicDetails.recruitment_status_of_trial_india != 'Not Applicable') and BasicDetails.recruitment_status_of_trial_global == 'Not Applicable' and BasicDetails.sample_size_from_india == BasicDetails.total_sample_size and BasicDetails.date_of_first_enrollment_global == 'Date Missing' and BasicDetails.type_of_trial == 'Interventional' and SitesOfStudy.city == 'South West');

**Command to extract cases with city name: West**

SELECT BasicDetails.ctri_number ,BasicDetails.year_of_reg, SitesOfStudy.city FROM BasicDetails, SitesOfStudy

WHERE ((SitesOfStudy.ctri_number == BasicDetails.ctri_number) and type_of_study == 'Drug' AND

BasicDetails.countries_of_recruitment == 'India' and (BasicDetails.recruitment_status_of_trial_india != '' and BasicDetails.recruitment_status_of_trial_india != 'Not Applicable') and BasicDetails.recruitment_status_of_trial_global == 'Not Applicable' and BasicDetails.sample_size_from_india == BasicDetails.total_sample_size and BasicDetails.date_of_first_enrollment_global == 'Date Missing' and BasicDetails.type_of_trial == 'Interventional' and SitesOfStudy.city == 'West');

**Command to extract cases with city name: North**

SELECT BasicDetails.ctri_number ,BasicDetails.year_of_reg, SitesOfStudy.city FROM BasicDetails, SitesOfStudy

WHERE ((SitesOfStudy.ctri_number == BasicDetails.ctri_number) and type_of_study == 'Drug' AND

BasicDetails.countries_of_recruitment == 'India' and (BasicDetails.recruitment_status_of_trial_india != '' and BasicDetails.recruitment_status_of_trial_india != 'Not Applicable') and BasicDetails.recruitment_status_of_trial_global == 'Not Applicable' and BasicDetails.sample_size_from_india == BasicDetails.total_sample_size and BasicDetails.date_of_first_enrollment_global == 'Date Missing' and BasicDetails.type_of_trial == 'Interventional' and SitesOfStudy.city == 'North');

**Command to extract cases with city name: North East**

SELECT BasicDetails.ctri_number ,BasicDetails.year_of_reg, SitesOfStudy.city FROM BasicDetails, SitesOfStudy

WHERE ((SitesOfStudy.ctri_number == BasicDetails.ctri_number) and type_of_study == 'Drug' AND

BasicDetails.countries_of_recruitment == 'India' and (BasicDetails.recruitment_status_of_trial_india != '' and BasicDetails.recruitment_status_of_trial_india != 'Not Applicable') and BasicDetails.recruitment_status_of_trial_global == 'Not Applicable' and BasicDetails.sample_size_from_india == BasicDetails.total_sample_size and BasicDetails.date_of_first_enrollment_global == 'Date Missing' and BasicDetails.type_of_trial == 'Interventional' and SitesOfStudy.city == 'North East');

**Command to extract cases with city name: North West**

SELECT BasicDetails.ctri_number ,BasicDetails.year_of_reg, SitesOfStudy.city FROM BasicDetails, SitesOfStudy

WHERE ((SitesOfStudy.ctri_number == BasicDetails.ctri_number) and type_of_study == 'Drug' AND

BasicDetails.countries_of_recruitment == 'India' and (BasicDetails.recruitment_status_of_trial_india != '' and BasicDetails.recruitment_status_of_trial_india != 'Not Applicable') and BasicDetails.recruitment_status_of_trial_global == 'Not Applicable' and BasicDetails.sample_size_from_india == BasicDetails.total_sample_size and BasicDetails.date_of_first_enrollment_global == 'Date Missing' and BasicDetails.type_of_trial == 'Interventional' and SitesOfStudy.city == 'North West');

Later, we manually grouped all the records with errors. Then, we used the pivot table option in Excel to generate the data in column H, '**Number of errors**'.

**Sheet name: 13.3c and 13.3d_Multinational_Incorrect listing of cities**

**Grouping of 6683 cases in years**

SELECT BasicDetails.year_of_reg, count(*) as 'type' FROM BasicDetails, SitesOfStudy

WHERE ((SitesOfStudy.ctri_number == BasicDetails.ctri_number) and type_of_study == 'Drug' AND

(BasicDetails.countries_of_recruitment like '%,India,%' or BasicDetails.countries_of_recruitment like 'India,%' or BasicDetails.countries_of_recruitment like '%,India' and BasicDetails.countries_of_recruitment !='India') and BasicDetails.recruitment_status_of_trial_india != 'Not Applicable' and BasicDetails.recruitment_status_of_trial_global != 'Not Applicable' and BasicDetails.sample_size_from_india != BasicDetails.total_sample_size and BasicDetails.sample_size_from_india != '"0"' and BasicDetails.sample_size_from_india != '""' and BasicDetails.date_of_first_enrollment_india != 'Date Missing' and BasicDetails.type_of_trial == 'Interventional' )group by BasicDetails.year_of_reg;

**Command to extract cases with city name: Mumbai (Suburban)**

SELECT BasicDetails.ctri_number ,BasicDetails.year_of_reg, SitesOfStudy.city FROM BasicDetails, SitesOfStudy

WHERE ((SitesOfStudy.ctri_number == BasicDetails.ctri_number) and type_of_study == 'Drug' AND

(BasicDetails.countries_of_recruitment like '%,India,%' or BasicDetails.countries_of_recruitment like 'India,%' or BasicDetails.countries_of_recruitment like '%,India' and BasicDetails.countries_of_recruitment !='India') and BasicDetails.recruitment_status_of_trial_india != 'Not Applicable' and BasicDetails.recruitment_status_of_trial_global != 'Not Applicable' and BasicDetails.sample_size_from_india != BasicDetails.total_sample_size and BasicDetails.sample_size_from_india != '"0"' and BasicDetails.sample_size_from_india != '""' and BasicDetails.date_of_first_enrollment_india != 'Date Missing' and BasicDetails.type_of_trial == 'Interventional' and SitesOfStudy.city == 'Mumbai (Suburban)');

**Command to extract cases with city name: Central**

SELECT BasicDetails.ctri_number ,BasicDetails.year_of_reg, SitesOfStudy.city FROM BasicDetails, SitesOfStudy

WHERE ((SitesOfStudy.ctri_number == BasicDetails.ctri_number) and type_of_study == 'Drug' AND

(BasicDetails.countries_of_recruitment like '%,India,%' or BasicDetails.countries_of_recruitment like 'India,%' or BasicDetails.countries_of_recruitment like '%,India' and BasicDetails.countries_of_recruitment !='India') and BasicDetails.recruitment_status_of_trial_india != 'Not Applicable' and BasicDetails.recruitment_status_of_trial_global != 'Not Applicable' and BasicDetails.sample_size_from_india != BasicDetails.total_sample_size and BasicDetails.sample_size_from_india != '"0"' and BasicDetails.sample_size_from_india != '""' and BasicDetails.date_of_first_enrollment_india != 'Date Missing' and BasicDetails.type_of_trial == 'Interventional' and SitesOfStudy.city == 'Central');

**Command to extract cases with city name: South**

SELECT BasicDetails.ctri_number ,BasicDetails.year_of_reg, SitesOfStudy.city FROM BasicDetails, SitesOfStudy

WHERE ((SitesOfStudy.ctri_number == BasicDetails.ctri_number) and type_of_study == 'Drug' AND

(BasicDetails.countries_of_recruitment like '%,India,%' or BasicDetails.countries_of_recruitment like 'India,%' or BasicDetails.countries_of_recruitment like '%,India' and BasicDetails.countries_of_recruitment !='India') and BasicDetails.recruitment_status_of_trial_india != 'Not Applicable' and BasicDetails.recruitment_status_of_trial_global != 'Not Applicable' and BasicDetails.sample_size_from_india != BasicDetails.total_sample_size and BasicDetails.sample_size_from_india != '"0"' and BasicDetails.sample_size_from_india != '""' and BasicDetails.date_of_first_enrollment_india != 'Date Missing' and BasicDetails.type_of_trial == 'Interventional' and SitesOfStudy.city == 'South');

**Command to extract cases with city name: North**

SELECT BasicDetails.ctri_number ,BasicDetails.year_of_reg, SitesOfStudy.city FROM BasicDetails, SitesOfStudy

WHERE ((SitesOfStudy.ctri_number == BasicDetails.ctri_number) and type_of_study == 'Drug' AND

(BasicDetails.countries_of_recruitment like '%,India,%' or BasicDetails.countries_of_recruitment like 'India,%' or BasicDetails.countries_of_recruitment like '%,India' and BasicDetails.countries_of_recruitment !='India') and BasicDetails.recruitment_status_of_trial_india != 'Not Applicable' and BasicDetails.recruitment_status_of_trial_global != 'Not Applicable' and BasicDetails.sample_size_from_india != BasicDetails.total_sample_size and BasicDetails.sample_size_from_india != '"0"' and BasicDetails.sample_size_from_india != '""' and BasicDetails.date_of_first_enrollment_india != 'Date Missing' and BasicDetails.type_of_trial == 'Interventional' and SitesOfStudy.city == 'North');

**Command to extract cases with city name: East**

SELECT BasicDetails.ctri_number ,BasicDetails.year_of_reg, SitesOfStudy.city FROM BasicDetails, SitesOfStudy

WHERE ((SitesOfStudy.ctri_number == BasicDetails.ctri_number) and type_of_study == 'Drug' AND

(BasicDetails.countries_of_recruitment like '%,India,%' or BasicDetails.countries_of_recruitment like 'India,%' or BasicDetails.countries_of_recruitment like '%,India' and BasicDetails.countries_of_recruitment !='India') and BasicDetails.recruitment_status_of_trial_india != 'Not Applicable' and BasicDetails.recruitment_status_of_trial_global != 'Not Applicable' and BasicDetails.sample_size_from_india != BasicDetails.total_sample_size and BasicDetails.sample_size_from_india != '"0"' and BasicDetails.sample_size_from_india != '""' and BasicDetails.date_of_first_enrollment_india != 'Date Missing' and BasicDetails.type_of_trial == 'Interventional' and SitesOfStudy.city == 'East');

**Command to extract cases with city name: South West**

SELECT BasicDetails.ctri_number ,BasicDetails.year_of_reg, SitesOfStudy.city FROM BasicDetails, SitesOfStudy

WHERE ((SitesOfStudy.ctri_number == BasicDetails.ctri_number) and type_of_study == 'Drug' AND

(BasicDetails.countries_of_recruitment like '%,India,%' or BasicDetails.countries_of_recruitment like 'India,%' or BasicDetails.countries_of_recruitment like '%,India' and BasicDetails.countries_of_recruitment !='India') and BasicDetails.recruitment_status_of_trial_india != 'Not Applicable' and BasicDetails.recruitment_status_of_trial_global != 'Not Applicable' and BasicDetails.sample_size_from_india != BasicDetails.total_sample_size and BasicDetails.sample_size_from_india != '"0"' and BasicDetails.sample_size_from_india != '""' and BasicDetails.date_of_first_enrollment_india != 'Date Missing' and BasicDetails.type_of_trial == 'Interventional' and SitesOfStudy.city == 'South West');

**Command to extract cases with city name: West**

SELECT BasicDetails.ctri_number ,BasicDetails.year_of_reg, SitesOfStudy.city FROM BasicDetails, SitesOfStudy

WHERE ((SitesOfStudy.ctri_number == BasicDetails.ctri_number) and type_of_study == 'Drug' AND

(BasicDetails.countries_of_recruitment like '%,India,%' or BasicDetails.countries_of_recruitment like 'India,%' or BasicDetails.countries_of_recruitment like '%,India' and BasicDetails.countries_of_recruitment !='India') and BasicDetails.recruitment_status_of_trial_india != 'Not Applicable' and BasicDetails.recruitment_status_of_trial_global != 'Not Applicable' and BasicDetails.sample_size_from_india != BasicDetails.total_sample_size and BasicDetails.sample_size_from_india != '"0"' and BasicDetails.sample_size_from_india != '""' and BasicDetails.date_of_first_enrollment_india != 'Date Missing' and BasicDetails.type_of_trial == 'Interventional' and SitesOfStudy.city == 'West');

**Command to extract cases with city name: North West**

SELECT BasicDetails.ctri_number ,BasicDetails.year_of_reg, SitesOfStudy.city FROM BasicDetails, SitesOfStudy

WHERE ((SitesOfStudy.ctri_number == BasicDetails.ctri_number) and type_of_study == 'Drug' AND

(BasicDetails.countries_of_recruitment like '%,India,%' or BasicDetails.countries_of_recruitment like 'India,%' or BasicDetails.countries_of_recruitment like '%,India' and BasicDetails.countries_of_recruitment !='India') and BasicDetails.recruitment_status_of_trial_india != 'Not Applicable' and BasicDetails.recruitment_status_of_trial_global != 'Not Applicable' and BasicDetails.sample_size_from_india != BasicDetails.total_sample_size and BasicDetails.sample_size_from_india != '"0"' and BasicDetails.sample_size_from_india != '""' and BasicDetails.date_of_first_enrollment_india != 'Date Missing' and BasicDetails.type_of_trial == 'Interventional' and SitesOfStudy.city == 'North West');

Later, we manually grouped all the records with errors. Then, we used the pivot table option in Excel to generate the data in column H, '**Number of errors**'.

---------------------------------------------------------------------------------------------------------------

**Additional file 14**

**Sheet name: 14.1a and 14.1b_Indian_PI name null_per year**

**1655 cases were generated for Additional file 12, ‘Sheet S12b_55 PMS cases_Errors over time’, and also used for this sheet.**

**Command used to extract 90 cases**

select PrincipalInvestigatorDetails.ctri_number, BasicDetails.year_of_reg from BasicDetails, PrincipalInvestigatorDetails

WHERE ( (PrincipalInvestigatorDetails.ctri_number == BasicDetails.ctri_number) and type_of_study == 'Drug' AND

BasicDetails.countries_of_recruitment == 'India' and BasicDetails.recruitment_status_of_trial_india != 'Not Applicable' and BasicDetails.recruitment_status_of_trial_global == 'Not Applicable' and BasicDetails.sample_size_from_india == BasicDetails.total_sample_size and BasicDetails.date_of_first_enrollment_global == 'Date Missing' and BasicDetails.type_of_trial == 'Interventional' and PrincipalInvestigatorDetails.name is null);

**Grouping of 90 cases in years**

select BasicDetails.year_of_reg , count(*) as 'Year' from BasicDetails, PrincipalInvestigatorDetails

WHERE ( (PrincipalInvestigatorDetails.ctri_number == BasicDetails.ctri_number) and type_of_study == 'Drug' AND

BasicDetails.countries_of_recruitment == 'India' and BasicDetails.recruitment_status_of_trial_india != 'Not Applicable' and BasicDetails.recruitment_status_of_trial_global == 'Not Applicable' and BasicDetails.sample_size_from_india == BasicDetails.total_sample_size and BasicDetails.date_of_first_enrollment_global == 'Date Missing' and BasicDetails.type_of_trial == 'Interventional' and PrincipalInvestigatorDetails.name is null) group by BasicDetails.year_of_reg;

**Sheet name: 14.2a and 14.2b_Multinational_PI name null_per year**

**Grouping of 606 cases in years**

SELECT BasicDetails.year_of_reg, count(*) as 'type'

FROM BasicDetails

WHERE (type_of_study == 'Drug' AND

(BasicDetails.countries_of_recruitment like '%,India,%' or BasicDetails.countries_of_recruitment like 'India,%' or BasicDetails.countries_of_recruitment like '%,India' and BasicDetails.countries_of_recruitment !='India') and BasicDetails.recruitment_status_of_trial_india != 'Not Applicable' and BasicDetails.recruitment_status_of_trial_global != 'Not Applicable' and BasicDetails.sample_size_from_india != BasicDetails.total_sample_size and BasicDetails.sample_size_from_india != '"0"' and BasicDetails.sample_size_from_india != '""' and BasicDetails.date_of_first_enrollment_india != 'Date Missing' and BasicDetails.type_of_trial == 'Interventional' ) group by BasicDetails.year_of_reg;

**Command used to extract the cases with PI names null**

select PrincipalInvestigatorDetails.ctri_number, BasicDetails.year_of_reg from BasicDetails, PrincipalInvestigatorDetails where ((PrincipalInvestigatorDetails.ctri_number == BasicDetails.ctri_number) and BasicDetails.type_of_study =='Drug' and (BasicDetails.countries_of_recruitment like '%,India,%' or BasicDetails.countries_of_recruitment like 'India,%' or BasicDetails.countries_of_recruitment like '%,India' and BasicDetails.countries_of_recruitment !='India') and BasicDetails.recruitment_status_of_trial_india != 'Not Applicable' and BasicDetails.recruitment_status_of_trial_global != 'Not Applicable' and BasicDetails.sample_size_from_india != BasicDetails.total_sample_size and BasicDetails.sample_size_from_india != '"0"' and BasicDetails.sample_size_from_india != '""' and BasicDetails.date_of_first_enrollment_india != 'Date Missing' and BasicDetails.type_of_trial == 'Interventional' and PrincipalInvestigatorDetails.name is null ) ;

**Command used to extract the cases with PI name is 'Not Applicable'**

select PrincipalInvestigatorDetails.ctri_number, BasicDetails.year_of_reg, PrincipalInvestigatorDetails.name from BasicDetails, PrincipalInvestigatorDetails where ((PrincipalInvestigatorDetails.ctri_number == BasicDetails.ctri_number) and BasicDetails.type_of_study =='Drug' and (BasicDetails.countries_of_recruitment like '%,India,%' or BasicDetails.countries_of_recruitment like 'India,%' or BasicDetails.countries_of_recruitment like '%,India' and BasicDetails.countries_of_recruitment !='India') and BasicDetails.recruitment_status_of_trial_india != 'Not Applicable' and BasicDetails.recruitment_status_of_trial_global != 'Not Applicable' and BasicDetails.sample_size_from_india != BasicDetails.total_sample_size and BasicDetails.sample_size_from_india != '"0"' and BasicDetails.sample_size_from_india != '""' and BasicDetails.date_of_first_enrollment_india != 'Date Missing' and BasicDetails.type_of_trial == 'Interventional' and PrincipalInvestigatorDetails.name == 'Not Applicable' ) ;

**Command used to extract the cases with PI name is 'Not applicable'**

select PrincipalInvestigatorDetails.ctri_number, BasicDetails.year_of_reg, PrincipalInvestigatorDetails.name from BasicDetails, PrincipalInvestigatorDetails where ((PrincipalInvestigatorDetails.ctri_number == BasicDetails.ctri_number) and BasicDetails.type_of_study =='Drug' and (BasicDetails.countries_of_recruitment like '%,India,%' or BasicDetails.countries_of_recruitment like 'India,%' or BasicDetails.countries_of_recruitment like '%,India' and BasicDetails.countries_of_recruitment !='India') and BasicDetails.recruitment_status_of_trial_india != 'Not Applicable' and BasicDetails.recruitment_status_of_trial_global != 'Not Applicable' and BasicDetails.sample_size_from_india != BasicDetails.total_sample_size and BasicDetails.sample_size_from_india != '"0"' and BasicDetails.sample_size_from_india != '""' and BasicDetails.date_of_first_enrollment_india != 'Date Missing' and BasicDetails.type_of_trial == 'Interventional' and PrincipalInvestigatorDetails.name == 'Not applicable' ) ;

**Command used to extract the cases with PI name is 'NIL'**

select PrincipalInvestigatorDetails.ctri_number, BasicDetails.year_of_reg, PrincipalInvestigatorDetails.name from BasicDetails, PrincipalInvestigatorDetails where ((PrincipalInvestigatorDetails.ctri_number == BasicDetails.ctri_number) and BasicDetails.type_of_study =='Drug' and (BasicDetails.countries_of_recruitment like '%,India,%' or BasicDetails.countries_of_recruitment like 'India,%' or BasicDetails.countries_of_recruitment like '%,India' and BasicDetails.countries_of_recruitment !='India') and BasicDetails.recruitment_status_of_trial_india != 'Not Applicable' and BasicDetails.recruitment_status_of_trial_global != 'Not Applicable' and BasicDetails.sample_size_from_india != BasicDetails.total_sample_size and BasicDetails.sample_size_from_india != '"0"' and BasicDetails.sample_size_from_india != '""' and BasicDetails.date_of_first_enrollment_india != 'Date Missing' and BasicDetails.type_of_trial == 'Interventional' and PrincipalInvestigatorDetails.name == 'NIL' ) ;

**Command used to extract the cases with PI name is 'nil'**

select PrincipalInvestigatorDetails.ctri_number, BasicDetails.year_of_reg, PrincipalInvestigatorDetails.name from BasicDetails, PrincipalInvestigatorDetails where ((PrincipalInvestigatorDetails.ctri_number == BasicDetails.ctri_number) and BasicDetails.type_of_study =='Drug' and (BasicDetails.countries_of_recruitment like '%,India,%' or BasicDetails.countries_of_recruitment like 'India,%' or BasicDetails.countries_of_recruitment like '%,India' and BasicDetails.countries_of_recruitment !='India') and BasicDetails.recruitment_status_of_trial_india != 'Not Applicable' and BasicDetails.recruitment_status_of_trial_global != 'Not Applicable' and BasicDetails.sample_size_from_india != BasicDetails.total_sample_size and BasicDetails.sample_size_from_india != '"0"' and BasicDetails.sample_size_from_india != '""' and BasicDetails.date_of_first_enrollment_india != 'Date Missing' and BasicDetails.type_of_trial == 'Interventional' and PrincipalInvestigatorDetails.name == 'nil' ) ;

Later, we manually grouped all the records with errors. Then, we used the pivot table option in Excel to generate the data in column G, '**Number of errors**'.

**Sheet name: 14.3a and 14.3b_Indian_Type of study NA_Phase available**

**Grouping of 261 in years**

SELECT BasicDetails.year_of_reg , count(*) as 'Year'

FROM BasicDetails

WHERE (BasicDetails.type_of_study == 'NA' and BasicDetails.countries_of_recruitment == 'India' and BasicDetails.recruitment_status_of_trial_india != 'Not Applicable' and BasicDetails.recruitment_status_of_trial_global == 'Not Applicable' and BasicDetails.sample_size_from_india == BasicDetails.total_sample_size and BasicDetails.date_of_first_enrollment_global == 'Date Missing' )group by BasicDetails.year_of_reg;

**Command used to extract cases with Phase 1**

SELECT BasicDetails.ctri_number,BasicDetails.year_of_reg, BasicDetails.type_of_study, BasicDetails.phase_of_trial

FROM BasicDetails

WHERE (BasicDetails.type_of_study == 'NA' and BasicDetails.countries_of_recruitment == 'India' and BasicDetails.recruitment_status_of_trial_india != 'Not Applicable' and BasicDetails.recruitment_status_of_trial_global == 'Not Applicable' and BasicDetails.sample_size_from_india == BasicDetails.total_sample_size and BasicDetails.date_of_first_enrollment_global == 'Date Missing' and BasicDetails.phase_of_trial== 'Phase 1' );

**Command used to extract cases with Phase 2**

SELECT BasicDetails.ctri_number,BasicDetails.year_of_reg, BasicDetails.type_of_study, BasicDetails.phase_of_trial

FROM BasicDetails

WHERE (BasicDetails.type_of_study == 'NA' and BasicDetails.countries_of_recruitment == 'India' and BasicDetails.recruitment_status_of_trial_india != 'Not Applicable' and BasicDetails.recruitment_status_of_trial_global == 'Not Applicable' and BasicDetails.sample_size_from_india == BasicDetails.total_sample_size and BasicDetails.date_of_first_enrollment_global == 'Date Missing' and BasicDetails.phase_of_trial== 'Phase 2' );

**Command used to extract cases with Phase 3**

SELECT BasicDetails.ctri_number,BasicDetails.year_of_reg, BasicDetails.type_of_study, BasicDetails.phase_of_trial

FROM BasicDetails

WHERE (BasicDetails.type_of_study == 'NA' and BasicDetails.countries_of_recruitment == 'India' and BasicDetails.recruitment_status_of_trial_india != 'Not Applicable' and BasicDetails.recruitment_status_of_trial_global == 'Not Applicable' and BasicDetails.sample_size_from_india == BasicDetails.total_sample_size and BasicDetails.date_of_first_enrollment_global == 'Date Missing' and BasicDetails.phase_of_trial== 'Phase 3' );

**Command used to extract cases with Phase 4**

SELECT BasicDetails.ctri_number,BasicDetails.year_of_reg, BasicDetails.type_of_study, BasicDetails.phase_of_trial

FROM BasicDetails

WHERE (BasicDetails.type_of_study == 'NA' and BasicDetails.countries_of_recruitment == 'India' and BasicDetails.recruitment_status_of_trial_india != 'Not Applicable' and BasicDetails.recruitment_status_of_trial_global == 'Not Applicable' and BasicDetails.sample_size_from_india == BasicDetails.total_sample_size and BasicDetails.date_of_first_enrollment_global == 'Date Missing' and BasicDetails.phase_of_trial== 'Phase 4' );

**Command used to extract cases with Phase 1/ Phase 2**

SELECT BasicDetails.ctri_number,BasicDetails.year_of_reg, BasicDetails.type_of_study, BasicDetails.phase_of_trial

FROM BasicDetails

WHERE (BasicDetails.type_of_study == 'NA' and BasicDetails.countries_of_recruitment == 'India' and BasicDetails.recruitment_status_of_trial_india != 'Not Applicable' and BasicDetails.recruitment_status_of_trial_global == 'Not Applicable' and BasicDetails.sample_size_from_india == BasicDetails.total_sample_size and BasicDetails.date_of_first_enrollment_global == 'Date Missing' and BasicDetails.phase_of_trial== 'Phase 1/ Phase 2' );

**Command used to extract cases with Phase 2/ Phase 3**

SELECT BasicDetails.ctri_number,BasicDetails.year_of_reg, BasicDetails.type_of_study, BasicDetails.phase_of_trial

FROM BasicDetails

WHERE (BasicDetails.type_of_study == 'NA' and BasicDetails.countries_of_recruitment == 'India' and BasicDetails.recruitment_status_of_trial_india != 'Not Applicable' and BasicDetails.recruitment_status_of_trial_global == 'Not Applicable' and BasicDetails.sample_size_from_india == BasicDetails.total_sample_size and BasicDetails.date_of_first_enrollment_global == 'Date Missing' and BasicDetails.phase_of_trial== 'Phase 2/ Phase 3' );

**Command used to extract cases with Phase 3/ Phase 4**

SELECT BasicDetails.ctri_number,BasicDetails.year_of_reg, BasicDetails.type_of_study, BasicDetails.phase_of_trial

FROM BasicDetails

WHERE (BasicDetails.type_of_study == 'NA' and BasicDetails.countries_of_recruitment == 'India' and BasicDetails.recruitment_status_of_trial_india != 'Not Applicable' and BasicDetails.recruitment_status_of_trial_global == 'Not Applicable' and BasicDetails.sample_size_from_india == BasicDetails.total_sample_size and BasicDetails.date_of_first_enrollment_global == 'Date Missing' and BasicDetails.phase_of_trial== 'Phase 3/ Phase 4' );

Later, we manually grouped all the records with errors. Then, we used the pivot table option in Excel to generate the data in column I, '**Number of errors**'.

**Sheet name: 14.4c_Multinational_Type of study NA _Phase available**

**Command used to extract cases with Phase 1**

SELECT BasicDetails.ctri_number,BasicDetails.year_of_reg, BasicDetails.type_of_study, BasicDetails.phase_of_trial

FROM BasicDetails

WHERE (BasicDetails.type_of_study == 'NA' and (BasicDetails.countries_of_recruitment like '%,India,%' or BasicDetails.countries_of_recruitment like 'India,%' or BasicDetails.countries_of_recruitment like '%,India' and BasicDetails.countries_of_recruitment !='India') and BasicDetails.recruitment_status_of_trial_india != 'Not Applicable' and BasicDetails.recruitment_status_of_trial_global != 'Not Applicable' and BasicDetails.sample_size_from_india != BasicDetails.total_sample_size and BasicDetails.sample_size_from_india != '"0"' and BasicDetails.sample_size_from_india != '""' and BasicDetails.date_of_first_enrollment_india != 'Date Missing' and BasicDetails.phase_of_trial== 'Phase 1' ) ;

**Command used to extract cases with Phase 3**

SELECT BasicDetails.ctri_number,BasicDetails.year_of_reg, BasicDetails.type_of_study, BasicDetails.phase_of_trial

FROM BasicDetails

WHERE (BasicDetails.type_of_study == 'NA' and (BasicDetails.countries_of_recruitment like '%,India,%' or BasicDetails.countries_of_recruitment like 'India,%' or BasicDetails.countries_of_recruitment like '%,India' and BasicDetails.countries_of_recruitment !='India') and BasicDetails.recruitment_status_of_trial_india != 'Not Applicable' and BasicDetails.recruitment_status_of_trial_global != 'Not Applicable' and BasicDetails.sample_size_from_india != BasicDetails.total_sample_size and BasicDetails.sample_size_from_india != '"0"' and BasicDetails.sample_size_from_india != '""' and BasicDetails.date_of_first_enrollment_india != 'Date Missing' and BasicDetails.phase_of_trial== 'Phase 3' ) ;

**Sheet name: 14.4a and 14.4b_Indian_Primary sponsor name NIL**

**1655 cases were generated for Additional file 12, ‘Sheet S12b_55 PMS cases_Errors over time’, and also used for this sheet.**

**Comamnd used to extract cases of primary sponsor name as NIL**

select PrimarySponsor.ctri_number, BasicDetails.year_of_reg, PrimarySponsor.name , BasicDetails.type_of_study

FROM BasicDetails, PrimarySponsor

WHERE ( (PrimarySponsor.ctri_number == BasicDetails.ctri_number) and BasicDetails.type_of_study == 'Drug' AND

BasicDetails.countries_of_recruitment == 'India' and BasicDetails.recruitment_status_of_trial_india != 'Not Applicable' and BasicDetails.recruitment_status_of_trial_global == 'Not Applicable' and BasicDetails.sample_size_from_india == BasicDetails.total_sample_size and BasicDetails.date_of_first_enrollment_global == 'Date Missing' and BasicDetails.type_of_trial == 'Interventional' and PrimarySponsor.name == 'NIL');

### **Comamnd used to extract cases of primary sponsor name as Nil**

select PrimarySponsor.ctri_number, BasicDetails.year_of_reg, PrimarySponsor.name , BasicDetails.type_of_study

FROM BasicDetails, PrimarySponsor

WHERE ( (PrimarySponsor.ctri_number == BasicDetails.ctri_number) and BasicDetails.type_of_study == 'Drug' AND

BasicDetails.countries_of_recruitment == 'India' and BasicDetails.recruitment_status_of_trial_india != 'Not Applicable' and BasicDetails.recruitment_status_of_trial_global == 'Not Applicable' and BasicDetails.sample_size_from_india == BasicDetails.total_sample_size and BasicDetails.date_of_first_enrollment_global == 'Date Missing' and BasicDetails.type_of_trial == 'Interventional' and PrimarySponsor.name == 'Nil');

**Comamnd used to extract cases of primary sponsor name as nil**

select PrimarySponsor.ctri_number, BasicDetails.year_of_reg, PrimarySponsor.name , BasicDetails.type_of_study

FROM BasicDetails, PrimarySponsor

WHERE ( (PrimarySponsor.ctri_number == BasicDetails.ctri_number) and BasicDetails.type_of_study == 'Drug' AND

BasicDetails.countries_of_recruitment == 'India' and BasicDetails.recruitment_status_of_trial_india != 'Not Applicable' and BasicDetails.recruitment_status_of_trial_global == 'Not Applicable' and BasicDetails.sample_size_from_india == BasicDetails.total_sample_size and BasicDetails.date_of_first_enrollment_global == 'Date Missing' and BasicDetails.type_of_trial == 'Interventional' and PrimarySponsor.name == 'nil');

Later, we manually grouped all the records with errors. Then, we used the pivot table option in Excel to generate the data in column I, '**Number of errors**'.

**Sheet name: 14.5a and 14.5b_Indian_State of trial site missing**

**Grouping of 6242 cases in years**

select BasicDetails.year_of_reg, count(*) as 'type' FROM BasicDetails, SitesOfStudy

WHERE ( (SitesOfStudy.ctri_number == BasicDetails.ctri_number) and type_of_study == 'Drug' AND

BasicDetails.countries_of_recruitment == 'India' and BasicDetails.recruitment_status_of_trial_india != 'Not Applicable' and BasicDetails.recruitment_status_of_trial_global == 'Not Applicable' and BasicDetails.sample_size_from_india == BasicDetails.total_sample_size and BasicDetails.date_of_first_enrollment_global == 'Date Missing' and BasicDetails.type_of_trial == 'Interventional') group by BasicDetails.year_of_reg;

**Command used to extract cases where the study state site is null**

select BasicDetails.ctri_number, BasicDetails.year_of_reg, SitesOfStudy.state FROM BasicDetails, SitesOfStudy

WHERE ( (SitesOfStudy.ctri_number == BasicDetails.ctri_number) and type_of_study == 'Drug' AND

BasicDetails.countries_of_recruitment == 'India' and BasicDetails.recruitment_status_of_trial_india != 'Not Applicable' and BasicDetails.recruitment_status_of_trial_global == 'Not Applicable' and BasicDetails.sample_size_from_india == BasicDetails.total_sample_size and BasicDetails.date_of_first_enrollment_global == 'Date Missing' and BasicDetails.type_of_trial == 'Interventional'and SitesOfStudy.city is null ) ;

**Grouping of cases with study state site: null in years**

select BasicDetails.year_of_reg, count(*) as 'count' FROM BasicDetails, SitesOfStudy

WHERE ( (SitesOfStudy.ctri_number == BasicDetails.ctri_number) and type_of_study == 'Drug' AND

BasicDetails.countries_of_recruitment == 'India' and BasicDetails.recruitment_status_of_trial_india != 'Not Applicable' and BasicDetails.recruitment_status_of_trial_global == 'Not Applicable' and BasicDetails.sample_size_from_india == BasicDetails.total_sample_size and BasicDetails.date_of_first_enrollment_global == 'Date Missing' and BasicDetails.type_of_trial == 'Interventional'and SitesOfStudy.city is null ) group by BasicDetails.year_of_reg ;

**Sheet name: 14.6a and 14.6b_Multinational_State of trial site missing**

**Grouping of 6683 cases in years**

select BasicDetails.year_of_reg, count(*) as 'type' FROM BasicDetails, SitesOfStudy

WHERE ( (SitesOfStudy.ctri_number == BasicDetails.ctri_number) and type_of_study == 'Drug' AND

(BasicDetails.countries_of_recruitment like '%,India,%' or BasicDetails.countries_of_recruitment like 'India,%' or BasicDetails.countries_of_recruitment like '%,India' and BasicDetails.countries_of_recruitment !='India') and BasicDetails.recruitment_status_of_trial_india != 'Not Applicable' and BasicDetails.recruitment_status_of_trial_global != 'Not Applicable' and BasicDetails.sample_size_from_india != BasicDetails.total_sample_size and BasicDetails.sample_size_from_india != '"0"' and BasicDetails.sample_size_from_india != '""' and BasicDetails.date_of_first_enrollment_india != 'Date Missing' and BasicDetails.type_of_trial == 'Interventional' ) group by BasicDetails.year_of_reg;

**Command used to extract cases where the study state site is null**

select BasicDetails.ctri_number, BasicDetails.year_of_reg, SitesOfStudy.state FROM BasicDetails, SitesOfStudy

WHERE ( (SitesOfStudy.ctri_number == BasicDetails.ctri_number) and type_of_study == 'Drug' AND

(BasicDetails.countries_of_recruitment like '%,India,%' or BasicDetails.countries_of_recruitment like 'India,%' or BasicDetails.countries_of_recruitment like '%,India' and BasicDetails.countries_of_recruitment !='India') and BasicDetails.recruitment_status_of_trial_india != 'Not Applicable' and BasicDetails.recruitment_status_of_trial_global != 'Not Applicable' and BasicDetails.sample_size_from_india != BasicDetails.total_sample_size and BasicDetails.sample_size_from_india != '"0"' and BasicDetails.sample_size_from_india != '""' and BasicDetails.date_of_first_enrollment_india != 'Date Missing' and BasicDetails.type_of_trial == 'Interventional' and SitesOfStudy.state is null ) ;

**Grouping of cases with study state site: null in years**

select BasicDetails.year_of_reg, count(*) as 'count' FROM BasicDetails, SitesOfStudy

WHERE ( (SitesOfStudy.ctri_number == BasicDetails.ctri_number) and type_of_study == 'Drug' AND

(BasicDetails.countries_of_recruitment like '%,India,%' or BasicDetails.countries_of_recruitment like 'India,%' or BasicDetails.countries_of_recruitment like '%,India' and BasicDetails.countries_of_recruitment !='India') and BasicDetails.recruitment_status_of_trial_india != 'Not Applicable' and BasicDetails.recruitment_status_of_trial_global != 'Not Applicable' and BasicDetails.sample_size_from_india != BasicDetails.total_sample_size and BasicDetails.sample_size_from_india != '"0"' and BasicDetails.sample_size_from_india != '""' and BasicDetails.date_of_first_enrollment_india != 'Date Missing' and BasicDetails.type_of_trial == 'Interventional' and SitesOfStudy.state is null ) group by BasicDetails.year_of_reg ;

---------------------------------------------------------------------------------------------------------------

**Additional file 15**

**Sheet name: 15.4a and 15.4b_Indian_# of ECs exceed # of sites**

**Grouping of 6129 cases in years**

select BasicDetails.year_of_reg, count(*) as 'type' from BasicDetails, EthicalCommittee where ((EthicalCommittee.ctri_number == BasicDetails.ctri_number) and BasicDetails.type_of_study =='Drug' AND

BasicDetails.countries_of_recruitment == 'India' and (BasicDetails.recruitment_status_of_trial_india != '' and BasicDetails.recruitment_status_of_trial_india != 'Not Applicable') and BasicDetails.recruitment_status_of_trial_global == 'Not Applicable' and BasicDetails.sample_size_from_india == BasicDetails.total_sample_size and BasicDetails.date_of_first_enrollment_global == 'Date Missing' and BasicDetails.type_of_trial == 'Interventional' )group by BasicDetails.year_of_reg;

On 6129 cases, IF condition was used in Excel to extract cases where the number of ethics committee exceeded the number of sites. Further, the 'Remove duplicates' option in Excel was used to remove the duplicate records. On this final ouput, the pivot table option in Excel was used to generate the data in column I, '**Number of errors**'.

**Sheet name: 15.5a and 15.5b_Multinational_# of ECs exceed # of sites**

**Grouping of 6623 in years**

select BasicDetails.year_of_reg, count(*) as 'type' from BasicDetails, EthicalCommittee where ((EthicalCommittee.ctri_number == BasicDetails.ctri_number) and BasicDetails.type_of_study =='Drug' AND

(BasicDetails.countries_of_recruitment like '%,India,%' or BasicDetails.countries_of_recruitment like 'India,%' or BasicDetails.countries_of_recruitment like '%,India' and BasicDetails.countries_of_recruitment !='India') and BasicDetails.recruitment_status_of_trial_india != 'Not Applicable' and BasicDetails.recruitment_status_of_trial_global != 'Not Applicable' and BasicDetails.sample_size_from_india != BasicDetails.total_sample_size and BasicDetails.sample_size_from_india != '"0"' and BasicDetails.sample_size_from_india != '""' and BasicDetails.date_of_first_enrollment_india != 'Date Missing' and BasicDetails.type_of_trial == 'Interventional') group by BasicDetails.year_of_reg;

On 6623 cases, IF condition was used in Excel to extract cases where the number of ethics committee exceeded the number of sites. Further, the 'Remove duplicates' option in Excel was used to remove the duplicate records. On this final ouput, the pivot table option in Excel was used to generate the data in column I, '**Number of errors**'.

---------------------------------------------------------------------------------------------------------------

**Additional file 16**

**Additional file 16b. The list of 47 trials conducted at the Malpani Multispeciality Hospital, Jaipur, Rajasthan**

From the SQLite database, SitesOfStudy table was saved as the SitesOfStudy.csv file and BasicDetails were saved as BasicDetails.csv.

Open office was used to extract this information.

Applied normal filter on the field named 'site' from file SitesOfStudy.csv and entered the word 'malpani' in search bar and got total 53 cases.

Out of 53, 6 non-relevant entries were manually removed, leaving 47 cases.

The CTRI IDs of 47 cases were matched with the source file 'BasicDetails.csv' using one line script - COUNTIF($A$2:$A$47, B2) to obtain the 'Recruitment status of trial global'

and 'Recruitment status of trial india'.
